# Enamel defects in *Acp4*^R110C/R110C^ mice and human *ACP4* mutations

**DOI:** 10.1038/s41598-022-20684-9

**Published:** 2022-10-01

**Authors:** Tian Liang, Shih-Kai Wang, Charles Smith, Hong Zhang, Yuanyuan Hu, Figen Seymen, Mine Koruyucu, Yelda Kasimoglu, Jung-Wook Kim, Chuhua Zhang, Thomas L. Saunders, James P. Simmer, Jan C.-C. Hu

**Affiliations:** 1 Department of Biologic and Materials Sciences, University of Michigan School of Dentistry, 1011 N University Ave, Ann Arbor, MI 48109 USA; 2 Department of Dentistry, National Taiwan University School of Dentistry, No. 1, Changde St., Zhongzheng Dist., Taipei City, 100 Taiwan; 3 Department of Pediatric Dentistry, National Taiwan University Children’s Hospital, No. 8, Zhongshan S. Rd., Zhongzheng Dist., Taipei City, 100 Taiwan; 4 Department of Anatomy & Cell Biology, Faculty of Medicine & Health Sciences, McGill University, Montreal, QC Canada; 5 Department of Pedodontics, Faculty of Dentistry, Altinbas University, 34147 Istanbul, Turkey; 6 Department of Pedodontics, Faculty of Dentistry, Istanbul University, 34116 Istanbul, Turkey; 7 Department of Molecular Genetics & Dental Research Institute, School of Dentistry, Seoul National University, Seoul, 03080 Republic of Korea; 8 Department of Pediatric Dentistry & Dental Research Institute, School of Dentistry, Seoul National University, Seoul, 03080 Republic of Korea; 9 Division of Molecular, Medicine and Genetics, Department of Internal Medicine, University of Michigan Medical School, Ann Arbor, MI 48109 USA

**Keywords:** Cell biology, Genetics

## Abstract

Human *ACP4* (OMIM*606362) encodes a transmembrane protein that belongs to histidine acid phosphatase (ACP) family. Recessive mutations in *ACP4* cause non-syndromic hypoplastic amelogenesis imperfecta (AI1J, OMIM#617297). While ACP activity has long been detected in developing teeth, its functions during tooth development and the pathogenesis of *ACP4*-associated AI remain largely unknown. Here, we characterized 2 AI1J families and identified a novel *ACP4* disease-causing mutation: c.774_775del, p.Gly260Aspfs*29. To investigate the role of ACP4 during amelogenesis, we generated and characterized *Acp4*^R110C^ mice that carry the p.(Arg110Cys) loss-of-function mutation. Mouse *Acp4* expression was the strongest at secretory stage ameloblasts, and the protein localized primarily at Tomes’ processes. While *Acp4* heterozygous (*Acp4*^+/R110C^) mice showed no phenotypes, incisors and molars of homozygous (*Acp4*^R110C/R110C^) mice exhibited a thin layer of aplastic enamel with numerous ectopic mineralized nodules. *Acp4*^R110C/R110C^ ameloblasts appeared normal initially but underwent pathology at mid-way of secretory stage. Ultrastructurally, sporadic enamel ribbons grew on mineralized dentin but failed to elongate, and aberrant needle-like crystals formed instead. Globs of organic matrix accumulated by the distal membranes of defective Tomes’ processes. These results demonstrated a critical role for ACP4 in appositional growth of dental enamel probably by processing and regulating enamel matrix proteins around mineralization front apparatus.

## Introduction

During the secretory stage of dental enamel formation, ameloblasts secrete large quantities of specialized phosphoproteins, including amelogenin, enamelin, and ameloblastin^1^. The quantity of secreted proteins increases steadily as secretory ameloblasts form the initial enamel mineral ribbons on the surface of dentin^2^ and, in mammals, reconfigure their distal membrane into a Tomes process^3–5^. The Tomes process is a specialization of the secretory ameloblast distal membrane that develops after formation of the initial enamel that includes a proximal part along the cell border with adjacent ameloblasts containing interrod grow sites and a protruding distal process containing rod growth sites^6,7^, and is responsible for the rod/interrod organization of mammalian enamel. A distal terminal web, comprised of filament bundles that run perpendicular to the long axis of the ameloblast, forms the proximal boundary of the Tomes' process^8–10^ and is connected to actin-rich terminal bar junctional complexes^9,11,12^, that may play a role in ameloblast movement^13,14^. The Tomes process is retracted near the end of the secretory stage.

Protein secretion remains high throughout the secretory stage and then decreases rapidly as ameloblasts undergo postsecretory transition and declines slowly thereafter as the shortened maturation ameloblasts modulate between ruffle-ended and smooth-ended forms along the surface of the maturing enamel^3,15^. Ameloblasts actively reabsorb enamel proteins into endosomes by endocytosis throughout the secretory and early maturation stages of amelogenesis^16–19^ and traffic the endosomes into lysosomes^20^. Lysosomes degrade reabsorbed matrix proteins and cellular constituents, including organelles and portions of cytosol that are sequestered in autophagosomes as well as cytosolic proteins targeted for lysosomal degradation by chaperone-mediated autophagy^21^. Lysosomes supply the cell with monomeric metabolites, such as amino acids, using selective transport proteins to transfer them from the lysosomal lumen and into the cytoplasm^22^.

Recessive mutations in *ACP4* (OMIM*606362) have recently been shown to cause non-syndromic amelogenesis imperfecta (AI, type IJ; OMIM#617297) in humans^23–25^ (Supplemental data Table S1) and dogs^26^. The defective enamel is typically hypoplastic (thin) and rough surfaced. Human *ACP4* encodes a single-pass type I membrane glycoprotein with a signal peptide (aa 1–26), an extracellular domain (aa 27–393; with N-glycosylation target sequences at residues N191, N269, N330, and N339, and disulfide bonds connecting C159–C378, C214–C312, and C353–C357), a transmembrane domain (aa 394–414) and a cytoplasmic tail (aa 415–426). ACP4 is most homologous to lysosomal (ACP2) and prostate acid phosphatases (ACP3) (Supplemental data Fig. S1), both of which belong to the histidine acid phosphatase family^27^ that is inhibited by tartrate^27^ and localize to the endosomal-lysosomal pathway^23,28^. It has long been known that an ACP2-like acid phosphatase is expressed by ameloblasts^29–33^. A tartrate-sensitive phosphatase localized strongest to the supranuclear region, and more weakly in the Tomes process^31^, and showed a pH optimum of 4.4^34,35^. Identification of *ACP4* mutations causing enamel malformations not only indicates a critical role for ACP4 in enamel formation but suggests that it might be the unidentified ACP2-like acid phosphatase found in ameloblasts. More recently, it has been shown that ACP4 serves an indispensable function only during tooth development, particularly enamel formation, as the gene is pseudogenized in mammals that have lost the ability to make teeth, or enamel, during evolution^36,37^.

Here we report two additional families with hypoplastic amelogenesis imperfecta (AI) caused by pathogenic variants in *ACP4*, analyze the expression of *Acp4* by mouse ameloblasts, and generate and characterize *Acp4*^R110C^ knockin mice to better understand the pathogenesis of *ACP4*-associated enamel defects and deduce the role of ACP4 during tooth development.

## Results

### Human *ACP4* mutations causing AI

**Figure 1:**
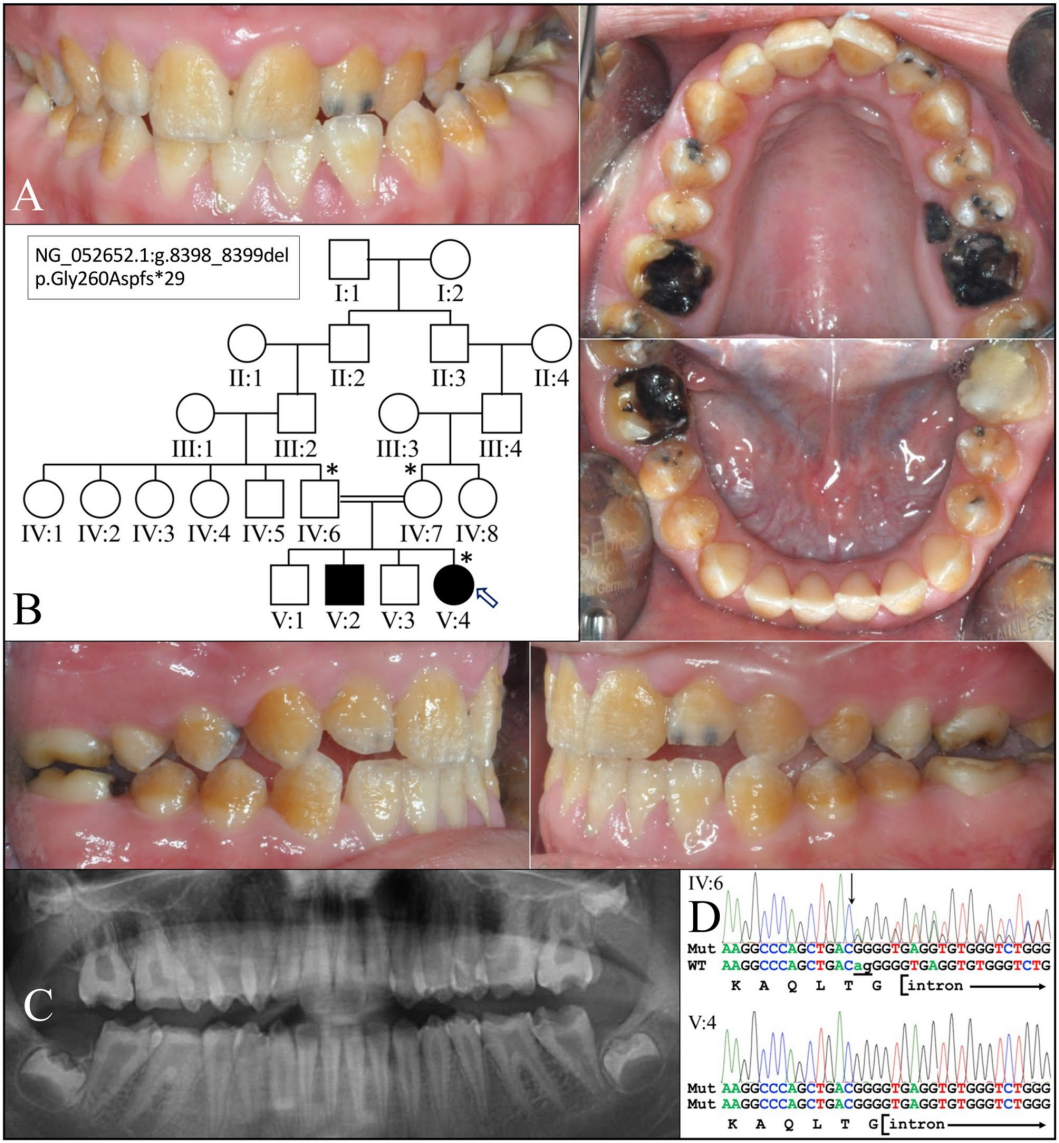
Family 1 with AI Type IJ (OMIM #617297). (**A**) Intraoral photograph of the proband (V:4) at age 14 showed a generalized severe enamel hypoplasia with rough surfaces. (**B**) The family pedigree indicated consanguinity and enamel defects inherited in an autosomal recessive pattern. (**C**) Proband’s panoramic radiograph demonstrated that the enamel in the proband was very thin. The dental pulp chambers appeared to be enlarged, and the first and second molars exhibited taurodontism. (**D**) DNA sequencing chromatograms revealed a novel *ACP4* mutation in Exon 7 (NG_052652.1:g.8398_8399del; NM_033068.3:c.774_775del; NP_149059.1: p.Gly260Aspfs*29) which was homozygous in the proband and heterozygous in both parents.

We characterized two consanguineous families from Turkey with autosomal recessive AI cosegregating with homozygous pathogenic variants in *ACP4*. The enamel of the family 1 proband (V:4; age 14) was thin with a rough surface and exhibited yellow–brown discoloration apparently from the dentin showing through the hypoplastic enamel (Fig. 1). The pulp chambers appeared to be larger than normal with taurodontism of the permanent molars. The novel *ACP4* mutation, a two-nucleotide deletion, in Exon 7 (NG_052652.1:g.8398_8399del; NM_033068.2:c.774_775del; NP_149059.1:p.Gly260Aspfs*29) was homozygous in the proband and heterozygous in both unaffected parents. The resulting mutant transcript would likely undergo nonsense mediated decay due to a premature termination codon. If the protein were generated, the frameshift would have deleted the C-terminal 167 amino acids [Gly260-Val426; including part of the extracellular (luminal) domain and all of the transmembrane and cytoplasmic domains] and replaced them with a 28-amino-acid extraneous peptide: DPAECYPCKLLPGPAPGAAPQDGHVLSS*. This *ACP4* sequence variation is rare: delAG = 0.00003 (7/251198, GnomAD_exome)^38^; delAG = 0.00004 (5/125568, TOPMED)^39^; delAG = 0.00003 (4/120806, ExAC)^40^. This brings to 11 the number of known *ACP4* pathogenic variants that cause AI (Supplemental data Table S1).

**Figure 2:**
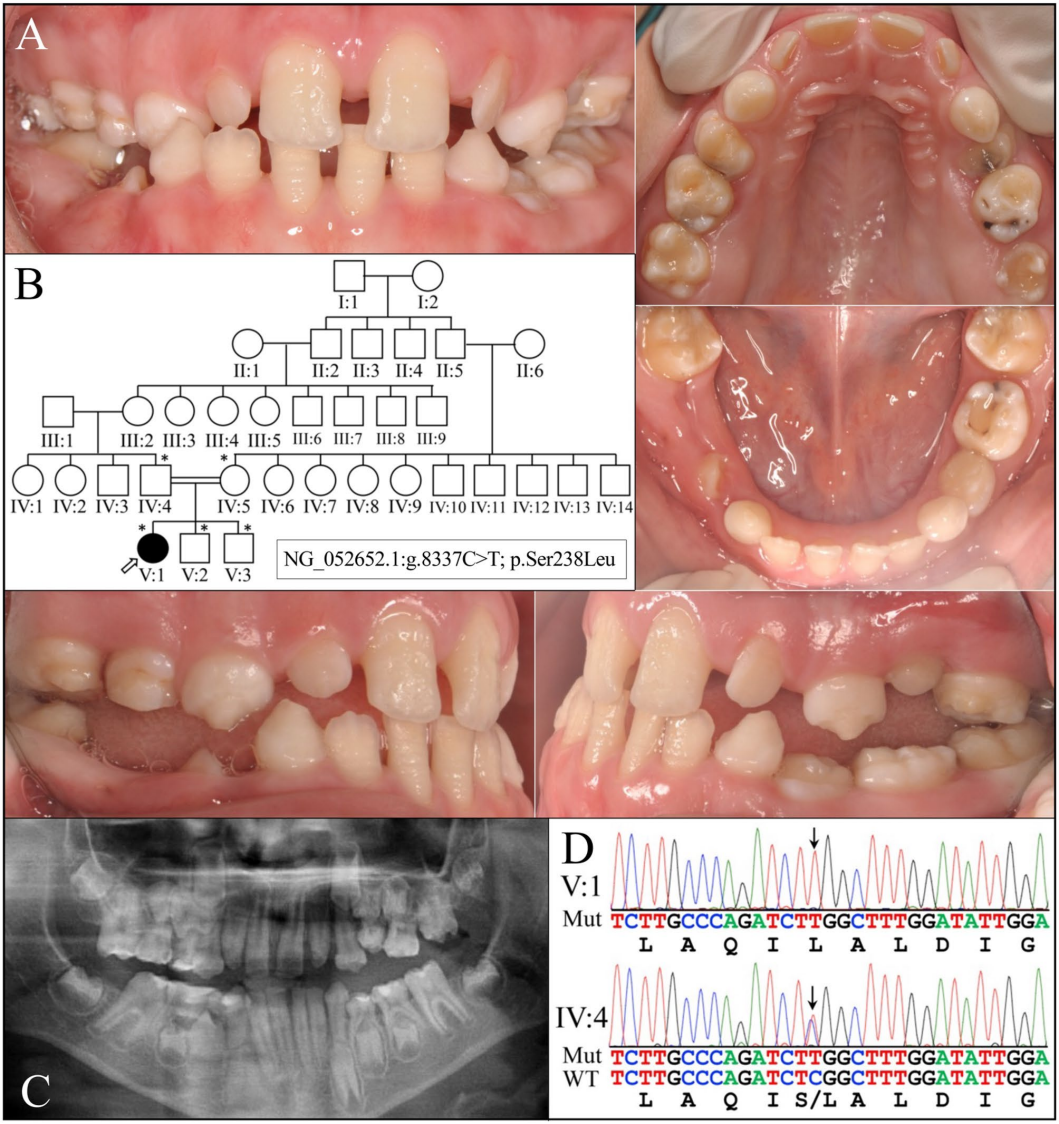
Family 2 with AI Type IJ (OMIM #617297). (**A**) Intraoral photograph of the proband (V:1) at age 9 showed a mixed dentition with enamel hypoplasia on both primary and permanent teeth. Horizonal hypoplastic bands were evident on the labial surface of lower incisors. (**B**) The pedigree indicated a 5-generation consanguineous family with the proband being the only individual with enamel malformations. (**C**) Panorex of the proband demonstrated that her unerupted teeth had extremely thin enamel, while the dentin and pulp chambers appeared normal. (**D**) DNA sequencing chromatograms revealed a reported *ACP4* mutation in Exon 7 (NG_052652.1:g.8337C>T; NM_033068.3:c.713C>T, NP_149059.1:p.Ser238Leu), which was homozygous in the proband and heterozygous in all of her immediate family members (IV:4, IV:5, V:2, and V:3).

In family 2 the enamel of the proband (V:1; age 9) was thin and rough, with horizontal hypoplastic grooves (Fig. 2). Penetration of the yellow dentin color through the enamel was less evident than in family 1. Both the primary and secondary dentitions were affected, and the permanent first molars showed mild taurodontism but no apparent enlarged pulp chambers. The pathogenic *ACP4* defect in Exon 7 (NG_052652.1:g.8337C>T; NM_033068.2:c.713C>T; NP_149059.1:p.Ser238Leu) was homozygous in the proband and heterozygous in her parents (IV:4; IV:5) and two siblings (V:2; V:3), who were all unaffected. This sequence variant was previously reported to cause AI^23^ and is listed in the dbSNP database as rs763573828. The mutation is rare: 2/251108, GnomAD_exome; 1/125568, TOPMED; 2/120550, ExAC. Sorting Intolerant from Tolerant (SIFT)^41^ gave it a score of 0.01 (deleterious), and Polymorphism Phenotyping v2 (Polyphen-2; HDIV) a score of 0.982 (probably damaging).

### *Acp4* transcript variation in mouse

Whereas only a single human *ACP4* mRNA reference sequence (NM_033068.3) was listed in the National Center for Biotechnology Information (NCBI) gene database, a cDNA reference sequence (NM_001195034.1) and seven *Acp4* transcript variant (TV) X files (TVX1, XM_006540530.5; TVX2, XM_006540531.4; TVX3, XM_017321901.2; TVX4, XM_017321902.3; TVX5, XR_003946367.1; TVX6; XM_011250772.3; TVX7: XM_030241931.2) were listed in the NCBI mouse gene database, and the list of transcripts continued up to TVX13 within the *Acp4* mouse genomic reference (NC_000073.7) file. Prior to settling upon a strategy to construct an *Acp4* knockout mouse, we analyzed the structure of mouse *Acp4* gene (Supplemental data Figs. S2–S6) with respect to its transcript variants, which varied at their 5′ ends (potentially due to variations in promoters/transcription initiation sites), and by alternative splicing that altered the 3' ends of the transcripts. The mouse *Acp4* transcript variation was potentially significant functionally, as some 5' variants eliminated the signal peptide (preventing ACP4 from targeting to the secretory pathway), while some 3' variants substituted Exon 12 with Exon 13, which replaced the C-terminal transmembrane and cytoplasmic domains, potentially causing the *Acp4* translation products to be released into the extracellular matrix rather than being retained in the membrane.

We conducted RT-PCR analyses of mRNA extracted from the enamel organ epithelia (EOE) dissected from D5 (secretory stage) and D11 (maturation stage) mouse first molars using a panel of PCR primers to determine which *Acp4* mRNA variants were expressed during enamel formation (Supplemental data Fig. S3). Only 2 *Acp4* TVs were detected: the reference sequence (Supplemental data Fig. S4) and a variant identical to the reference sequence except that Exon 12 was substituted with Exon 13 (Supplemental data Fig. S5). This transcript does not match any of the 13 variants currently listed at NCBI. Based upon protein domain prediction, the translation product of this *Acp4* variant would have a signal peptide but no transmembrane domain and probably be secreted. From these results we concluded that *Acp4* exon 1c (encoding the signal peptide) and exons 3 through 11 are included on all mouse *Acp4* mRNA transcripts expressed by the enamel organ epithelia (Supplemental data Fig. S6).

Based upon these findings we used the CRISPR/Cas9 system to knock in a TGC cysteine codon at position 110, replacing the CGG arginine codon to generate *Acp4*^R110C^ mice. The resulting p.Arg110Cys sequence variation was homologous to the human *ACP4* pathogenic mutation (p.Arg111Cys) that causes AI^23^ (Supplemental data Figs. S7 and S8). This modification occurred in exon 4, which was expressed on both transcripts we detected in EOE. The founders were back-crossed with C57BL/6N wild-type mice for seven generations to remove potential off-target effects of the CRISPER/Cas9 editing process and to maintain the mutation in the C57BL/6N genetic background.

### Spatial and temporal analyses of *Acp4* expression

**Figure 3:**
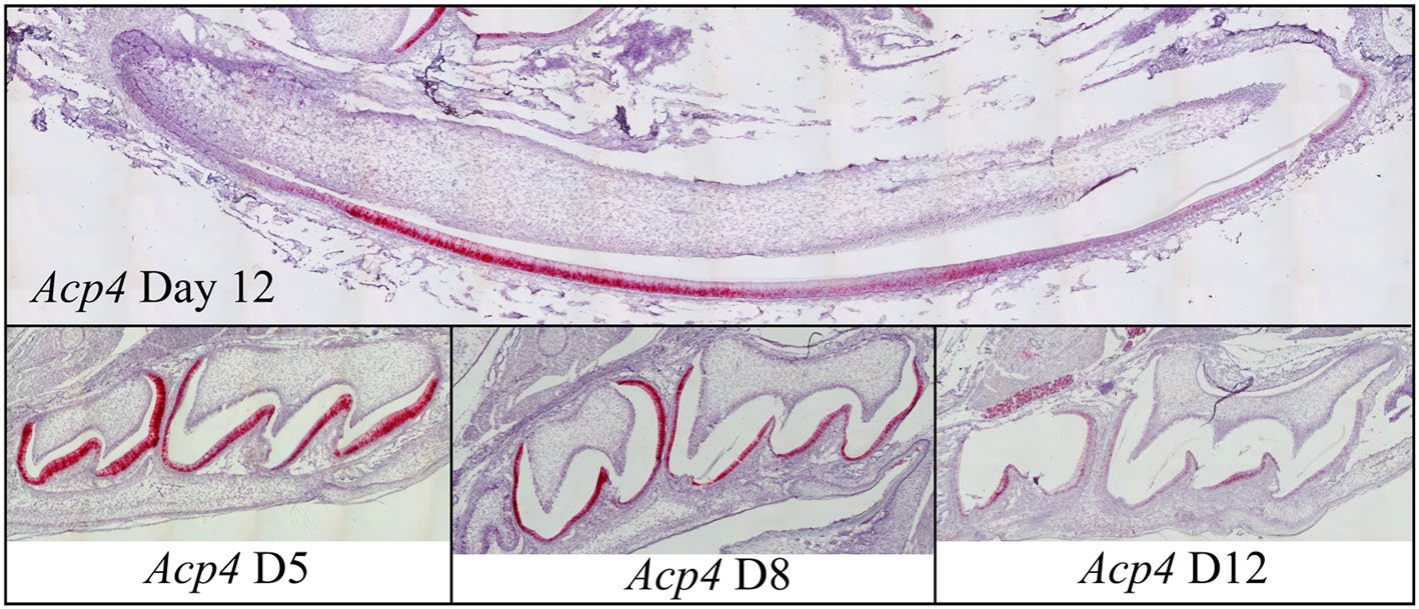
In situ Hybridization of *Acp4* in Mouse Incisor and Molars. Upper panel: sagittal section of a mouse hemimandible on postnatal day 12 (D12) showing the continuously growing mandibular incisor where enamel formation progressively advances from basal (left) to incisal (right). *Acp4* mRNA is strongly positive in incisor secretory ameloblasts, detectable at lower levels in the incisor maturation ameloblasts, and negative for odontoblasts and any other tissues on the section. Lower panel: at D5 maxillary first and second molars are in the secretory stage of amelogenesis. While most ameloblasts show strong positive signal, those at enamel-free area are negative for *Acp4*. In D8 and D12 maxillary first molars, *Acp4* mRNA shows reduced, but detectable expression in post-secretory and maturation ameloblasts.

To discern the timing of *Acp4* expression in mice, we conducted in situ hybridization studies on sagittal sections of hemimandibles from D12 wild-type mice using custom *Acp4* riboprobes. Developing mandibular incisors displayed all stages of amelogenesis in a linear array and gave a clear picture of the timing of *Acp4* expression (Fig. 3). *Acp4* mRNA was specific for ameloblasts and was strongest in secretory ameloblasts. The signal diminished, but continued in post-secretory transition and in maturation ameloblasts. Little, if any, signal was detected in odontoblasts and all other tissues. A similar pattern was observed in developing maxillary molars where *Acp4* signal was strongest during the secretory stage (D5), diminished during post-secretory transition (D8), and was still evident in maturation ameloblasts (D12). Expression of the *Acp4*^R110C^ mutant transcript was detected at levels similar to the wild-type *Acp4* until the appearance of ameloblast cell pathology during mid-secretory stage in the *Acp4*^R110C/R110C^ mice (Supplemental data Fig. S9).

**Figure 4:**
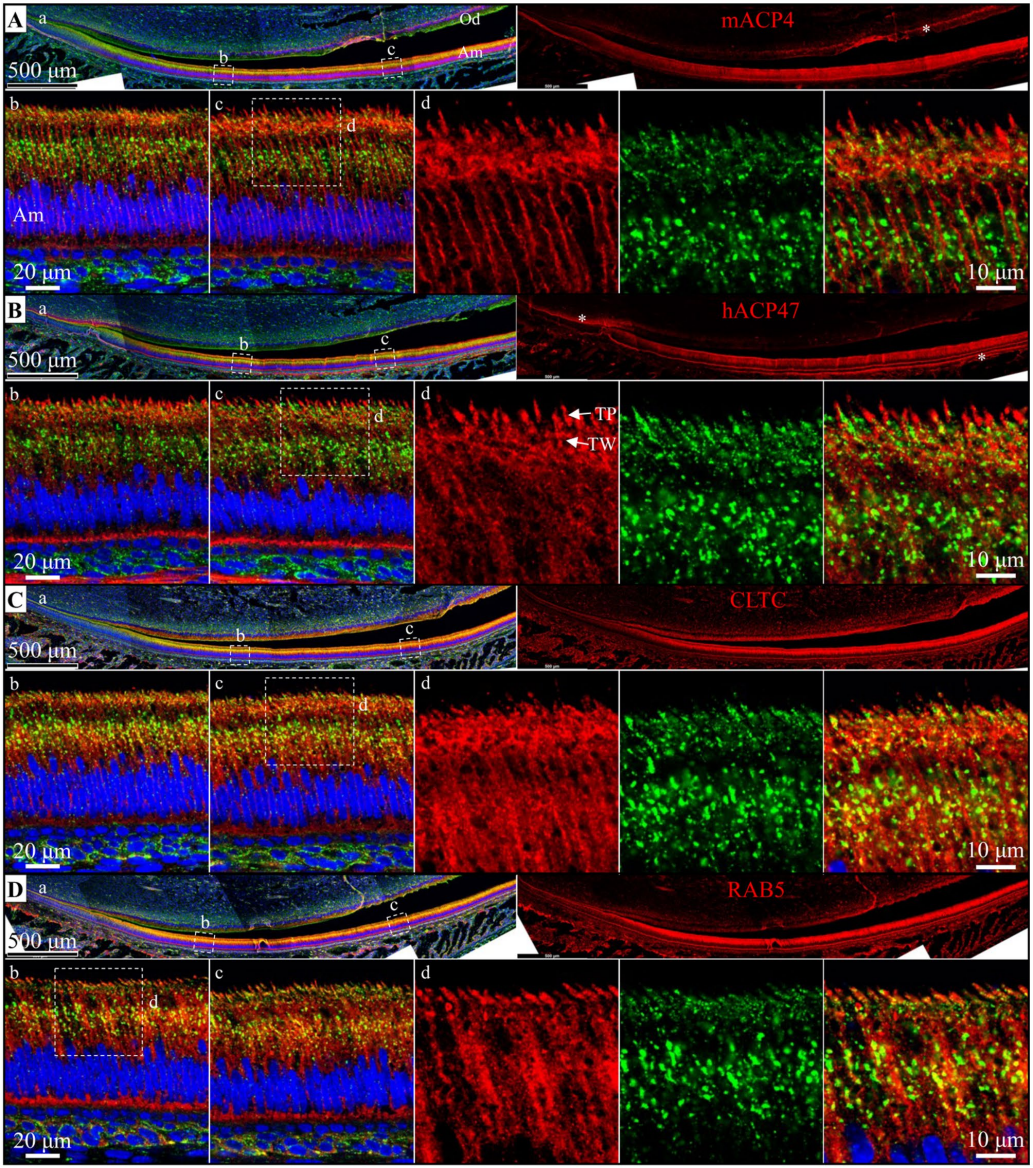
Immunohistochemistry of ACP4, LAMP1, CLTC, and RAB5 Expression in Consecutive, Longitudinally-Sectioned D10 Mouse Mandibular Incisors. Sections were immunostained red using antibodies against mACP4 (**A**), hACP4 (**B**), CLTC (clathrin-coated vesicles; **C**), and RAB5 (early endosomes; **D**) or immunostained green for LAMP1 (lysosomes and late endosomes). DAPI was used to stain cell nuclei blue. a. Tri-stained (left) and individual antibody-stained (right) apical half of mandibular incisors with box outlines of regions shown in higher magnification in b and c); b. Early and c. Mid-secretory stage ameloblasts. d. Three higher magnification images of the distal region of secretory stage ameloblasts (outlined in b or c) and immunostained for the target protein (red, left), LAMP1 (green, middle); or tri-stained (right). Am, ameloblasts; Od, odontoblasts; TP, Tomes' processes; TW, terminal web. ACP4 and LAMP1 signals were minimally overlapping suggesting ACP4 is not a lysosomal enzyme. ACP4 localizes to the Tomes' processes distally (potentially in endosomes and/or the distal membrane) as well as in the ameloblast lateral plasma membrane. These results are consistent with ACP4 dephosphorylating enamel matrix proteins in the matrix near the plasma membrane and in early endosomes. More immunostaining is shown in Supplemental Figs. S10–S14.

Expression and localization of the ACP4 protein by secretory ameloblasts were characterized in D12 mouse mandibular incisors (Fig. 4; Supplemental data Figs. S10–S13). Lysosome-Associated Membrane Protein 1 (LAMP1) was used as a lysosomal marker^42^, RAB5 as an early endosome marker^43^, and clathrin (CLTC) as a marker for coated vesicles and coated pits involved in endocytosis^43^. In secretory stage ameloblasts, ACP4 immunostaining was restricted largely to the Tomes’ process, the specialized secretory organ comprising the distal membrane of secretory ameloblasts. RAB5 showed significant overlap with LAMP1 expression only in the Tomes’ process and cytoplasm, whereas ACP4 showed minimal overlap with LAMP1 localization. On the other hand, the localization of CLTC and LAMP1 was significantly overlapping.

### Dental phenotype of *Acp4*^R110C/R110C^ mice

**Figure 5:**
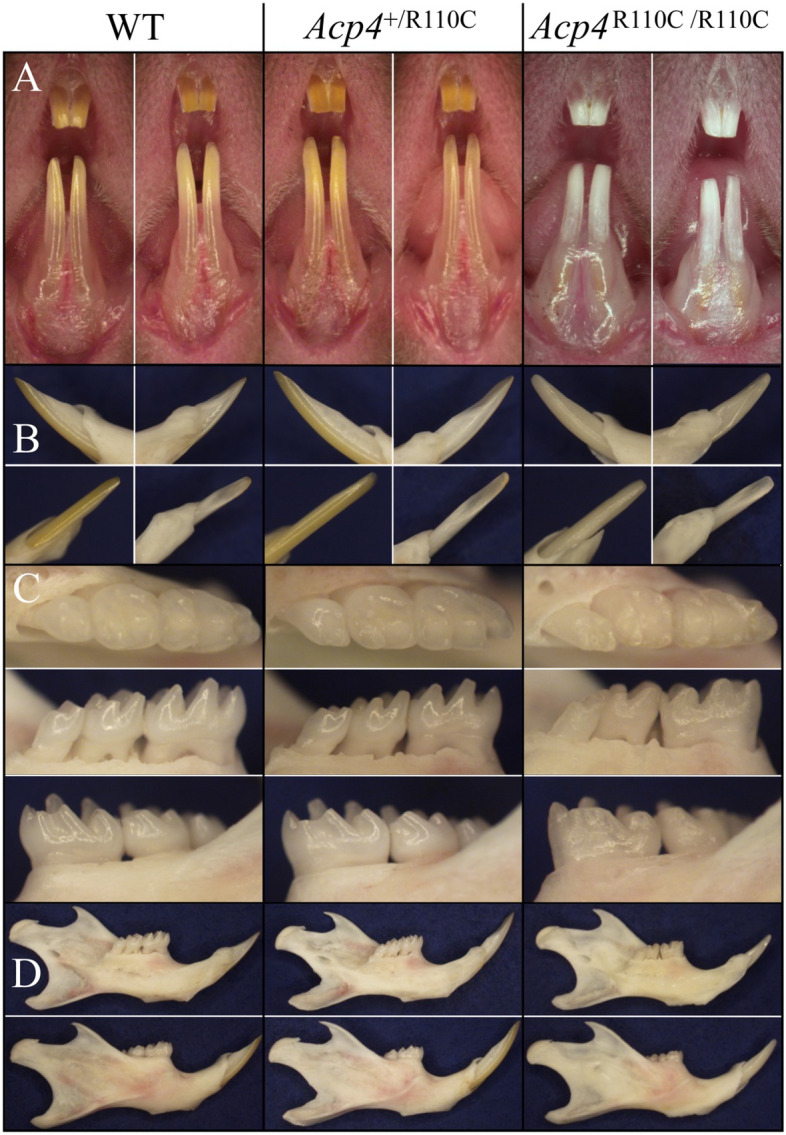
Dental Phenotype of *Acp4*^+/+^, *Acp4*^+/R110C^, and *Acp4*^R110C/R110C^ 7-week-old Mice. There were no appreciable differences in dental phenotype observed between *Acp4*^+/+^ and *Acp4*^+/R110C^ mice. (**A**) Frontal view of maxillary and mandibular incisors. The *Acp4*^R110C/R110C^ incisors appeared chalky white, and the enamel had abraded from dentin surface down to the cervical region and made the incisors appear shorter. (**B**) Medial (upper left), lateral (upper right), lingual (lower right), and labial (lower left) views of mandibular incisors. The *Acp4*^R110C/R110C^ incisal edge was flat and appeared shorter than that of *Acp4*^+/+^ due to attrition and chipping. (**C**) Occlusal (upper), lingual (middle), and buccal (lower) views of mandibular molars. No differences in alveolar bone level were observed among the three genotypes. The *Acp4*^R110C/R110C^ molars showed a dull and rough appearance and significant attrition. (**D**) Medial (upper) and lateral (lower) views of the hemimandible. No overt bony abnormality was observed for the *Acp4*^R110C/R110C^ mandible.

We first characterized the dental phenotype of 7-week mice of all three genotypes under a dissection microscope (Fig. 5). The heterozygous (*Acp4*^+/R110C^) mice appeared to be normal in all respects, while the homozygous (*Acp4*^R110C/R110C^) mice could be readily distinguished from their wild-type (*Acp4*^+/+^) and heterozygous (*Acp4*^+/R110C^) littermates because of their obvious enamel defects. In contrast to *Acp4*^+/+^ and *Acp4*^+/R110C^ mice, the *Acp4*^R110C/R110C^ incisors appeared chalky-white and both the incisors and molars underwent rapid attrition, showing blunt incisal tips and molar cusps. No apparent abnormalities were noted in bony structures of the *Acp4*^R110C/R110C^ mandible.

**Figure 6:**
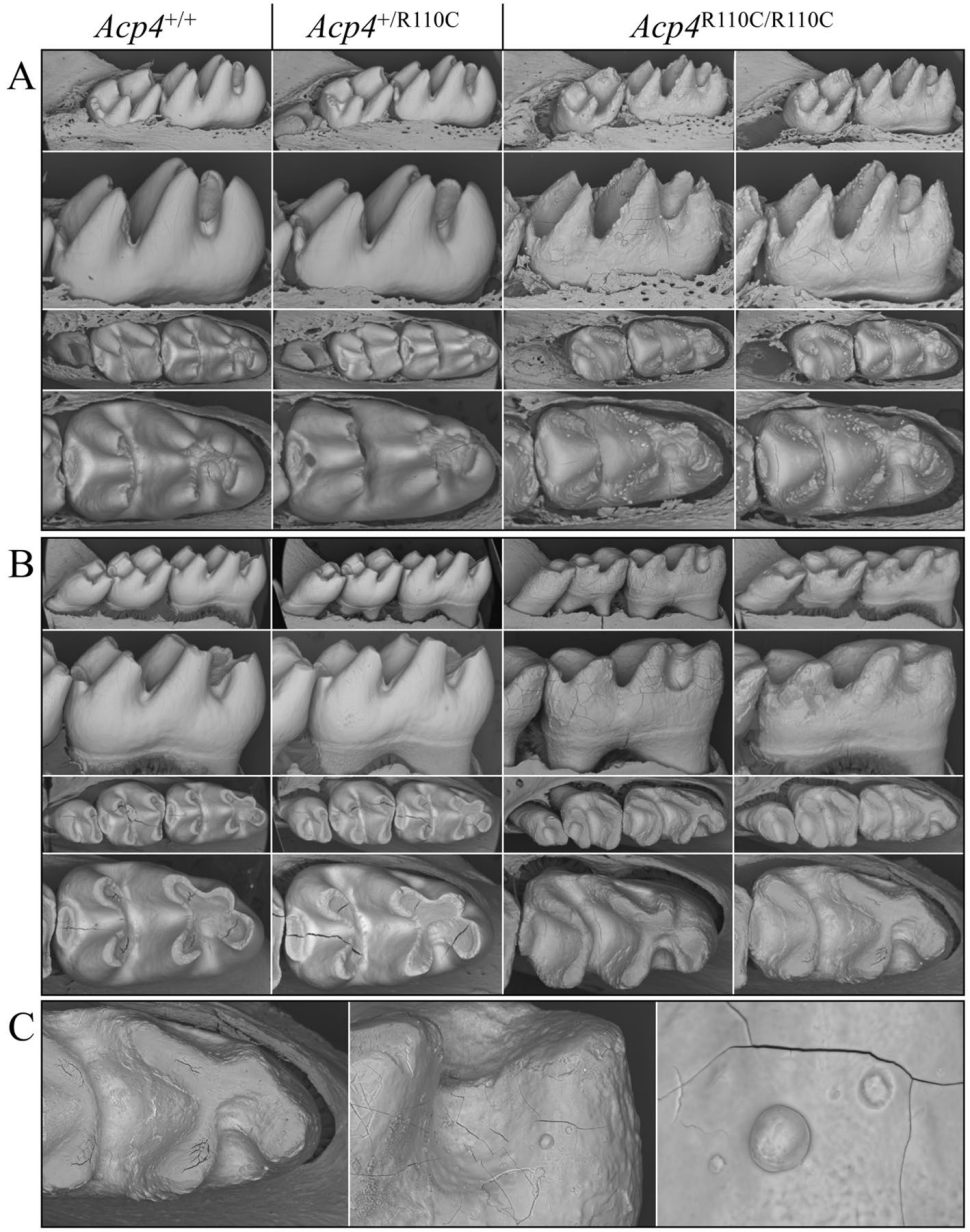
Backscattered SEM Images of *Acp4*^+/+^, *Acp4*^+/R110C^, and *Acp4*^R110C/R110C^ Mandibular Molars from D14 and 7-week Mice. (**A**) At D14 the mandibular first molars are very close to erupting into the oral cavity. The soft tissues have been removed to see the completed crown surface prior to potential post-eruptive changes. (**B**) The 7-week molars have been in function for about 5 weeks and feeding on a soft chow diet. (**C**) Close views of a 7-week *Acp4*^R110C/R110C^ mandibular first molar. The *Acp4*^+/+^ and *Acp4*^+/R110C^ molars are indistinguishable. The cusps of D14 *Acp4*^R110C/R110C^ molars appear slender and pointed as the mineralized material covering them is much thinner than normal enamel. This layer of aplastic enamel is studded with small nodules that give the surface a rough appearance. The 7-week *Acp4*^R110C/R110C^ molars show severe attrition and dentin exposure due to the absence of a true enamel layer. Most mineralized nodules that appeared at D14 have been abraded or chipped away, while some remain on the tooth surface.

We further conducted backscattered SEM (bSEM) analyses for D14 and 7-week mandibular molars (Fig. 6). At D14, the first molar was fully developed but not yet erupted into function where it could be altered by occlusal forces. Compared to *Acp4*^+/+^ and *Acp4*^+/R110C^, the D14 *Acp4*^R110C/R110C^ first molars had thin, pointed cusps because of the thinness of the enamel layer and a rough surface due to numerous ectopic mineralized nodules. By 7-weeks (5-weeks after their eruption into function) the first molars had already undergone rapid attrition, leaving shortened cusps, as observed with dissection microscopy.

**Figure 7:**
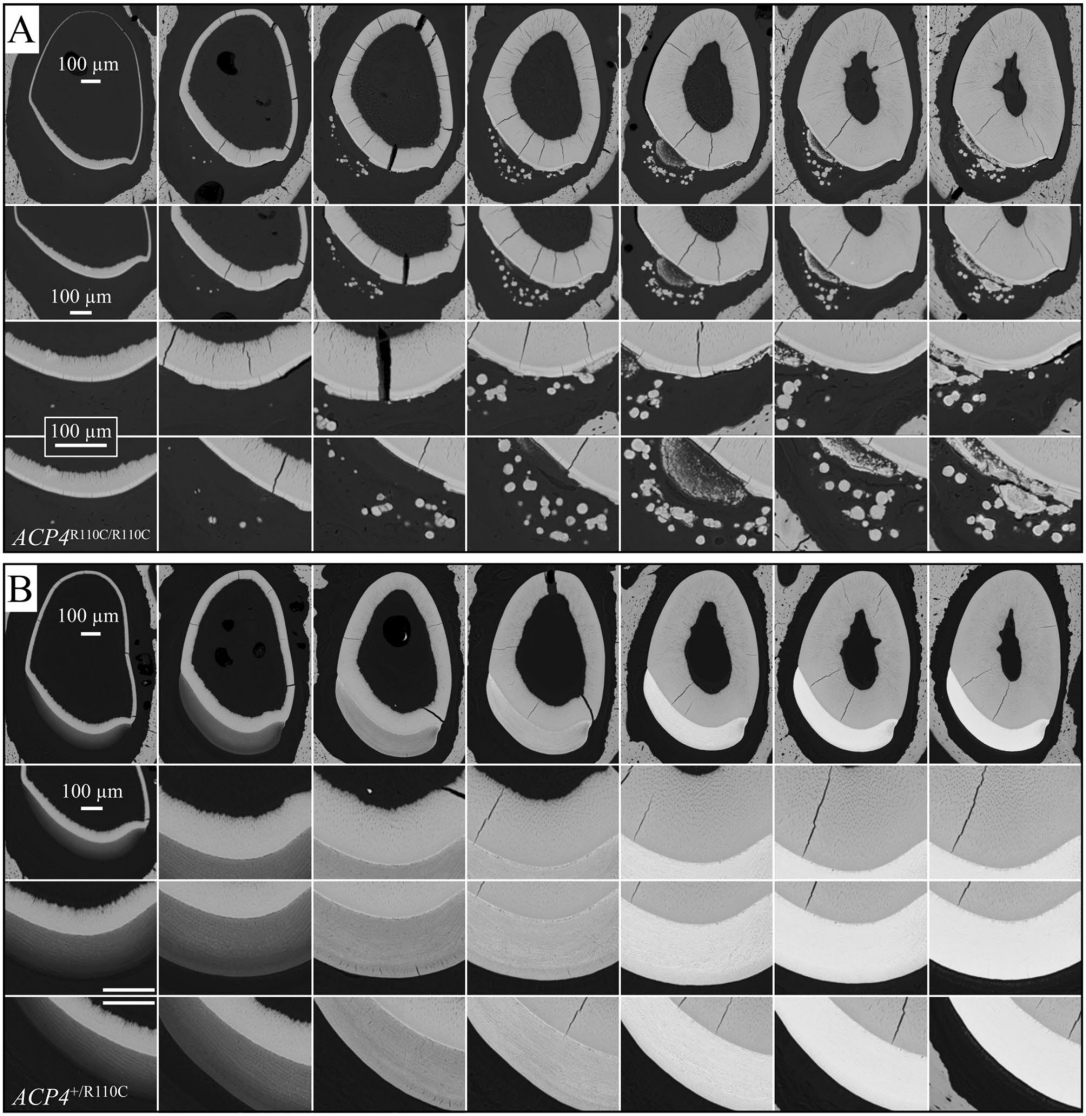
Backscattered SEM Images of *Acp4*^R110C/R110C^ and *Acp4*^+/+^ 7-week Mandibular Incisors. The incisors were cross-sectioned at 1 mm increments (levels 2 through 8). The first two columns (levels 2 and 3) show the secretory stage of enamel formation, where the enamel mineral ribbons elongate to their full length and the enamel layer achieves its final thickness, contour, and rod/interrod organization. Columns 3 through 7 (levels 4 through 8) show enamel maturation, where the thin crystals deposited during the secretory stage grow in width and thickness and the enamel layer as a whole achieves its final degree of mineralization. (**A**) *Acp4*^R110C/R110C^ incisors have a thin, disorganized layer of mineralized material that forms on the surface of dentin, before cell pathology advances to the point where mineral is ectopically deposited within the cellular layer and grows into nodules that are often not attached to the enamel surface. In contrast, (**B**) the *Acp4*^+/R110C^ enamel appears to be totally normal, suggesting that half the normal amount of ACP4 enzyme is sufficient to avoid pathology.

A standardized bSEM analysis of 7-week-old mandibular incisor cross-sections taken at 1 mm increments was also performed to visualize the progress of tooth mineralization^44–50^. Levels 1 through 3 corresponded to the secretory stage where the enamel layer expands as the enamel mineral ribbons elongate, while levels 4 through 8 showed enamel maturation where the enamel layer increases in degree of mineralization without changing the dimensions of the enamel layer as a whole (Fig. 7; Supplemental data Figs. S15–S17). The *Acp4*^R110C/R110C^ incisor appeared to deposit a thin layer of “enamel” on the surface of dentin, before mineral was ectopically deposited and hardened into nodules. While the *Acp4*^+/R110C^ heterozygous incisor showed a gradually increasing thickness of enamel layer from Level 2 to Level 3, the *Acp4*^R110C/R110C^ homozygous incisor failed to expand the enamel layer after Level 2. However, the hypoplastic “enamel” layer was hypercalcified from the start, displaying a higher electron density than the normal enamel of *Acp4*^+/R110C^ incisor at corresponding levels. Noticeably, more mineral nodules gradually formed labial to the enamel surface, within the enamel organ and connective tissues. At Level 8, where the *Acp4*^+/R110C^ enamel had reached a full thickness of ~ 121 μm and high density, the *Acp4*^R110C/R110C^ enamel below the ectopic mineral crust appeared to be only ~ 15 μm in thickness. The mineral density of the surface of the crust was higher than that of dentin, but much lower than normal enamel. Some of the mineral nodules showed concentric rings. The *Acp4*^+/R110C^ enamel appeared to be totally normal, suggesting that half the normal amount of ACP4 enzyme is sufficient to avoid pathology. It also suggested that the mutant ACP4 protein was not causing dominant negative effects.

### Histological analyses of tooth development in *Acp4*^R110C/R110C^ mice

**Figure 8:**
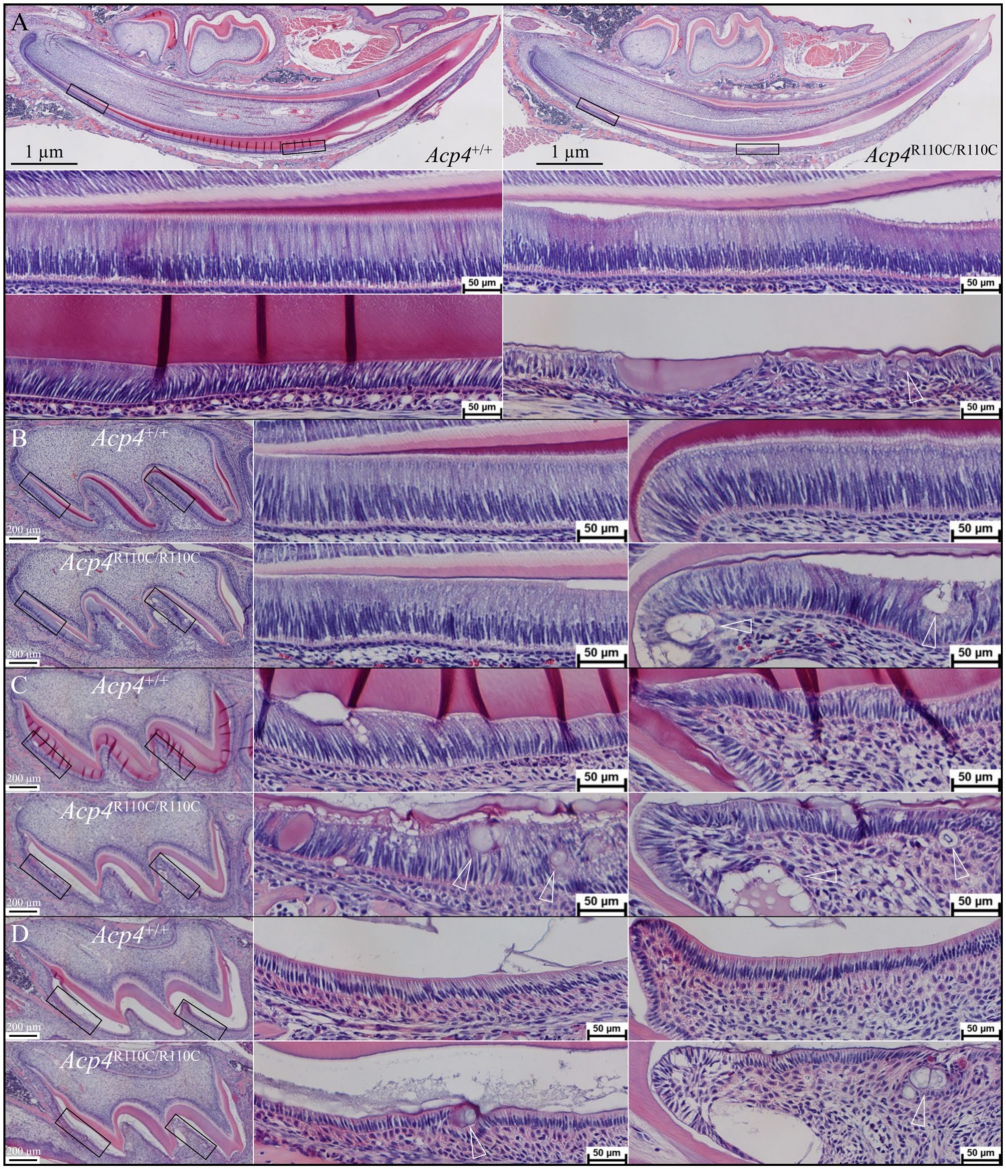
Histology of Sagittally Sectioned, H&E Stained, Developing Incisors and Molars from *Acp4*^+/+^ and *Acp4*^R110C/R110C^ Mice. (**A**) Histology of D12 mandibular incisors. Top: sections through *Acp4*^+/+^ (left) and *Acp4*^R110C/R110C^ (right) hemimandibles (40×). Black rectangles outline positions of higher magnification (200×) images shown below, covering the initiation of enamel formation (middle) and post-secretory transition (bottom). Note that in *Acp4*^R110C/R110C^ mice no proper enamel matrix is formed. Ameloblasts detach but remain polarized during the secretory stage (middle), but progressively degenerate as pathological ectopic mineral nodules (arrowhead) form in the soft tissue. (**B**–**D**) Histology of D4 (**B**), D8 (**C**), and D12 (**D**) *Acp4*^+/+^ and *Acp4*^R110C/R110C^ maxillary first molars. Left: whole molar sections with black rectangles outlining ameloblasts on the mesial and distal cusps shown at higher magnification (200×) toward the right. As in the incisors, in *Acp4*^R110C/R110C^ molars no proper enamel formation was observed. Ameloblasts polarized but detached from the mineral surface, ectopic mineral nodules formed (arrowheads) and expanded, and cell pathology worsened with time.

To investigate the pathogenesis of the observed dental defects, we compared the histology of sagittal sections of D12 mandibular incisors (Fig. 8, top) and D4, D8, and D12 maxillary first molars (Fig. 8, bottom) from *Acp4*^+/+^ and *Acp4*^R110C/R110C^ mice. In the *Acp4*^R110C/R110C^ incisors, ameloblasts differentiated into a recognizable ameloblast layer, but the enamel layer itself was noticeably thinner than normal, and segments of ameloblasts lost their sheet-like morphology and seemed to roll-up into a small matrix-containing cyst or nodule. The nodules increased in size, frequency, and degree of mineralization as the process of incisor formation progressed. Typically, a layer of squamous or short cuboidal epithelial cells surrounded the ectopic mineral nodules. In D4 maxillary first molars the nodules appeared to be more cyst-like and within the ameloblast layer. In D8 molars many nodules, that varied in their degree of mineralization, were evident within the ameloblast sheet and behind it, further away from the irregular enamel surface. By D12 the nodules were more numerous and mineralized, with many of them observed to be clustered together. However, unlike those of D12 incisors, the maturation stage ameloblasts of molars at most areas maintained recognizable morphology and normal cell polarity. The mineralized nodules we observed here corresponded to those found in bSEM analyses of *Acp4*^R110C/R110C^ incisors and molars.

To further examine how ameloblasts were altered, we examined the histology of D12 *Acp4*^R110C/R110C^ incisors that were immunostained for ß-actin and one of the major secreted enamel proteins: amelogenin (Supplemental data Fig. S18), enamelin (Supplemental data Fig. S19), and ameloblastin (Supplemental data Fig. S20). The ß-actin staining clearly outlined the sheet of ameloblasts by highlighting the proximal and distal poles of the ameloblasts in green. In general, amelogenin (AMEL), enamelin (ENAM), and ameloblastin (AMBN) expression appeared to proceed normally until midway through the secretory stage when the ameloblast layer separated from the enamel surface, the ameloblasts lost polarity, and the linear pattern of actin staining at the distal ends of the ameloblasts dispersed. The ectopic nodules were positively stained for all three enamel proteins. Furthermore, expression of the mutant ACP4 p.R110C protein was detected and was targeted normally to the Tomes' process in D5 *Acp4*^R110C/R110C^ maxillary molars (Supplemental data Fig. S21).

### Ultrastructural characterization of early amelogenesis

**Figure 9:**
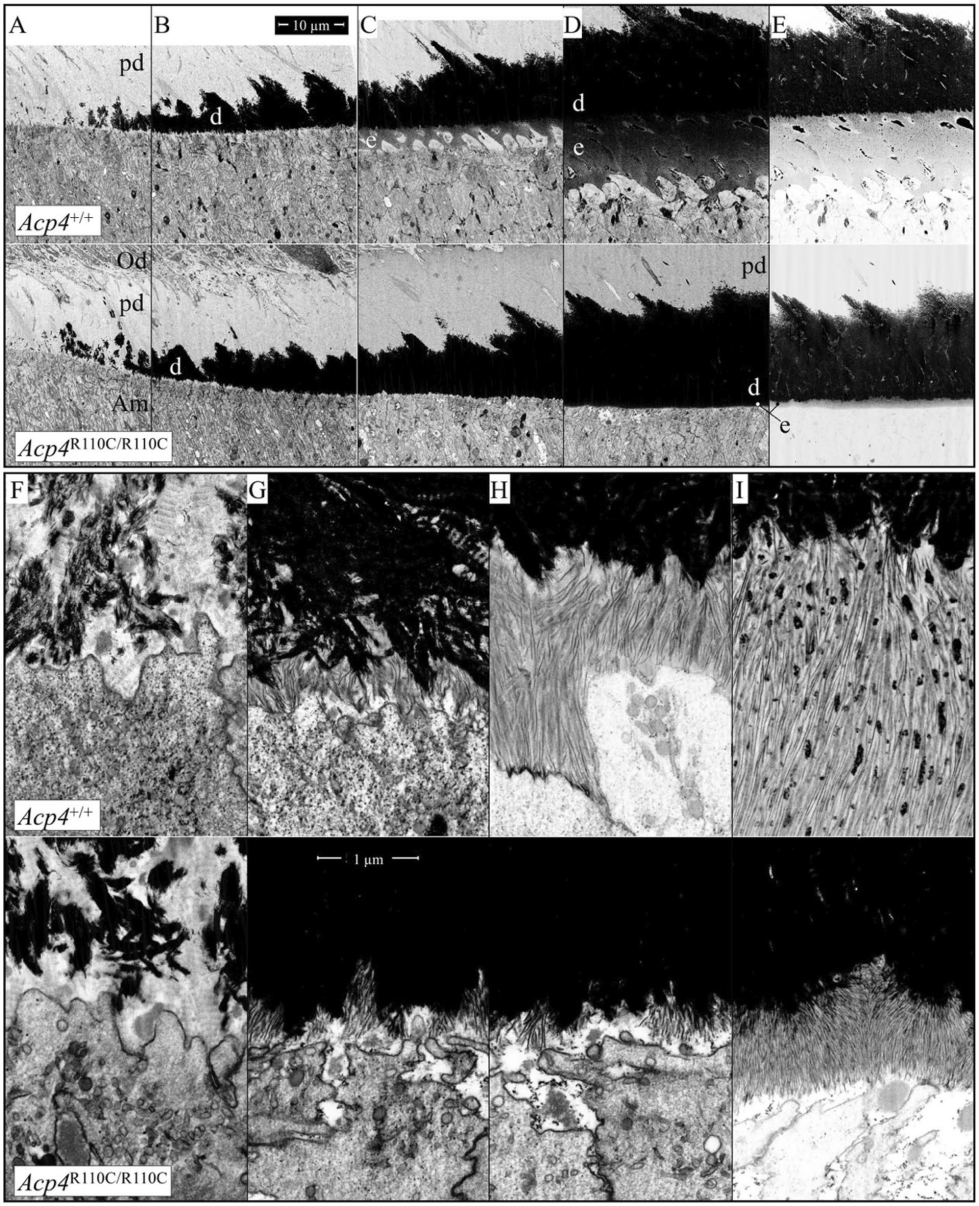
FIB-bSEM of 7-week *Acp4*^+/+^ and *Acp4*^R110C/R110C^ Mandibular Incisors. To compare the ultrastructure of early enamel formed in *Acp4*^+/+^ and *Acp4*^R110C/R110C^ mice, sites are sampled (1) after dentin mineralizes and before enamel secretion begins (**A**, **F**; Level 0.92), (2) just after enamel secretion starts (**B**, **G**; level 1.11), (3) transition from formation of the initial enamel layer to development of Tomes processes (**C**, **H**; level 1.20), and (4) early inner enamel layer formation (**D**, **I**; Level 1.33). (**E**) A brighter version of image of (**D**). Low magnification (mag) images (**A**–**E**) (2500×) with mag bar in (**B**); high mag images (**F**–**I**) (35,000×) with mag bar in (**G**). In *Acp4*^+/+^ mice, initial enamel mineral ribbons appear to elongate away from the tips and sides of mineralized collagen fibers at the DEJ (**A**, **B**). This process continues as the enamel prongs map out the Tomes processes (**C**) and continue on with formation of rod and interrod areas typical of the inner enamel layer (**D**). In *Acp4*^R110C/R110C^ mice, initial enamel ribbons start to form sporadically (**F**, **G**), but ameloblasts are unable to elongate them to create an initial layer, enamel prongs or Tomes processes (**G**, **H**), thereby negating any further normal development of an enamel layer. Noticeably, those initial ribbons are not associated with the ameloblast cell membrane. The mineral crystals formed in the absence of normal ACP4 appear thin, short in length and closely compressed close to each other (**I**). Am, ameloblasts; d, dentin; e, enamel; Od, odontoblasts; pd: predentin.

**Figure 10:**
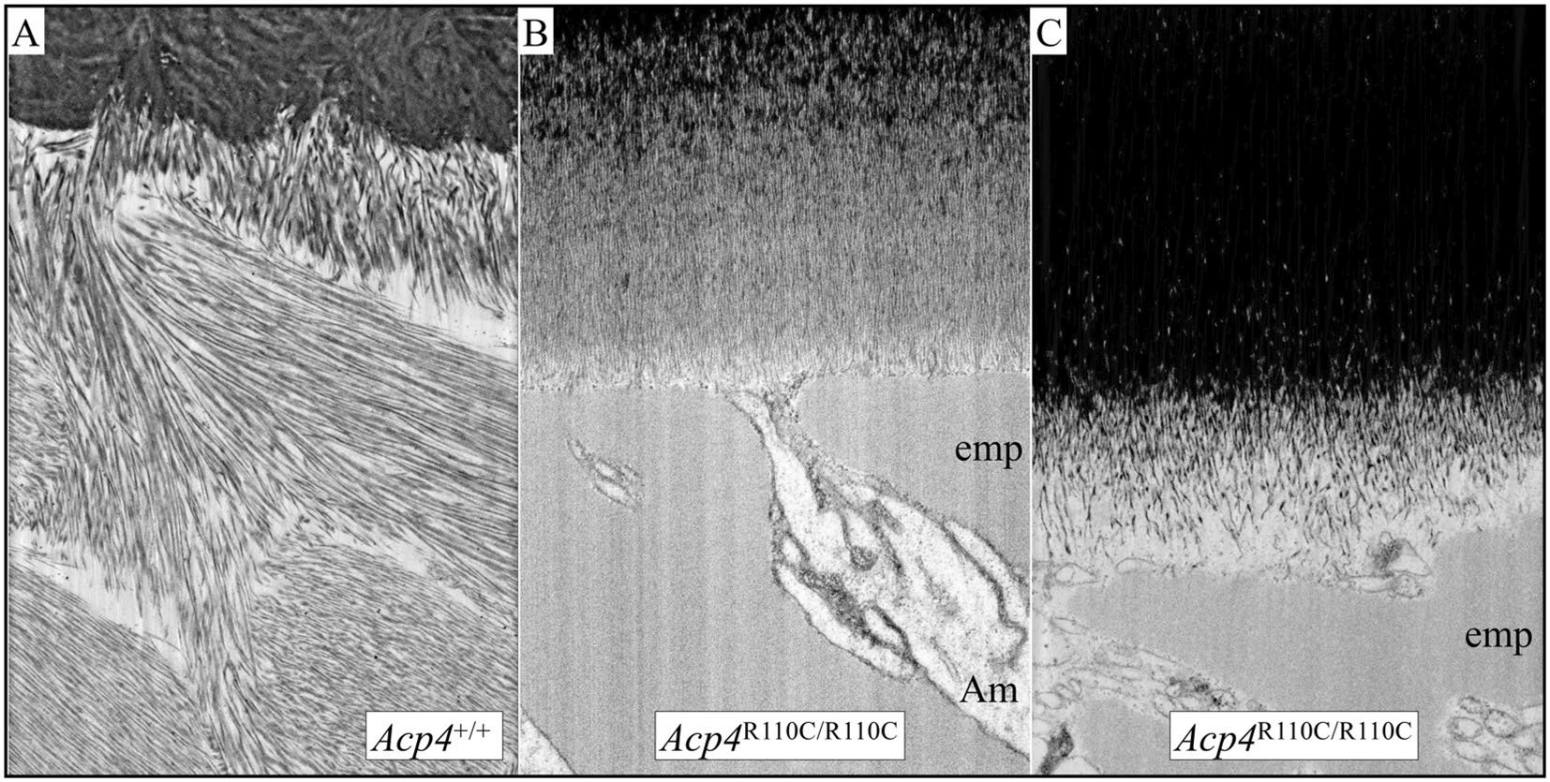
FIB-bSEM of 7-week *Acp4*^+/+^ and *Acp4*^R110C/R110C^ Mandibular Incisors (continued). The images show enamel formed later during the secretory stage in (**A**) *Acp4*^+/+^ and (**B**, **C**) *Acp4*^R110C/R110C^ incisors (all at 20,000×). At the position where *Acp4*^+/+^ ameloblasts have formed an enamel layer that is ~ 30 µm in thickness (7-wk incisor Level 1.5; DEJ area shown in **A**), the *Acp4*^R110C/R110C^ ameloblasts have only deposited an aplastic enamel layer about 6 µm thick on the DEJ (**B**). Abundant enamel proteins have also accumulated on top of the mineralized portion, and the distal ends of ameloblasts retain only finger-like point contacts/attachment with the mineralized surface (**B**). By roughly the midpoint of the secretory stage (Level 2, **C**), the enamel organ in *Acp4*^R110C/R110C^ mice degenerates into a disorganized mass of cells. The precise location of the DEJ becomes indistinct due to increased mineralization of the deeper parts of the aplastic enamel layer, which is now about 11 µm in thickness. Am, ameloblasts; emp, enamel matrix proteins.

**Figure 11:**
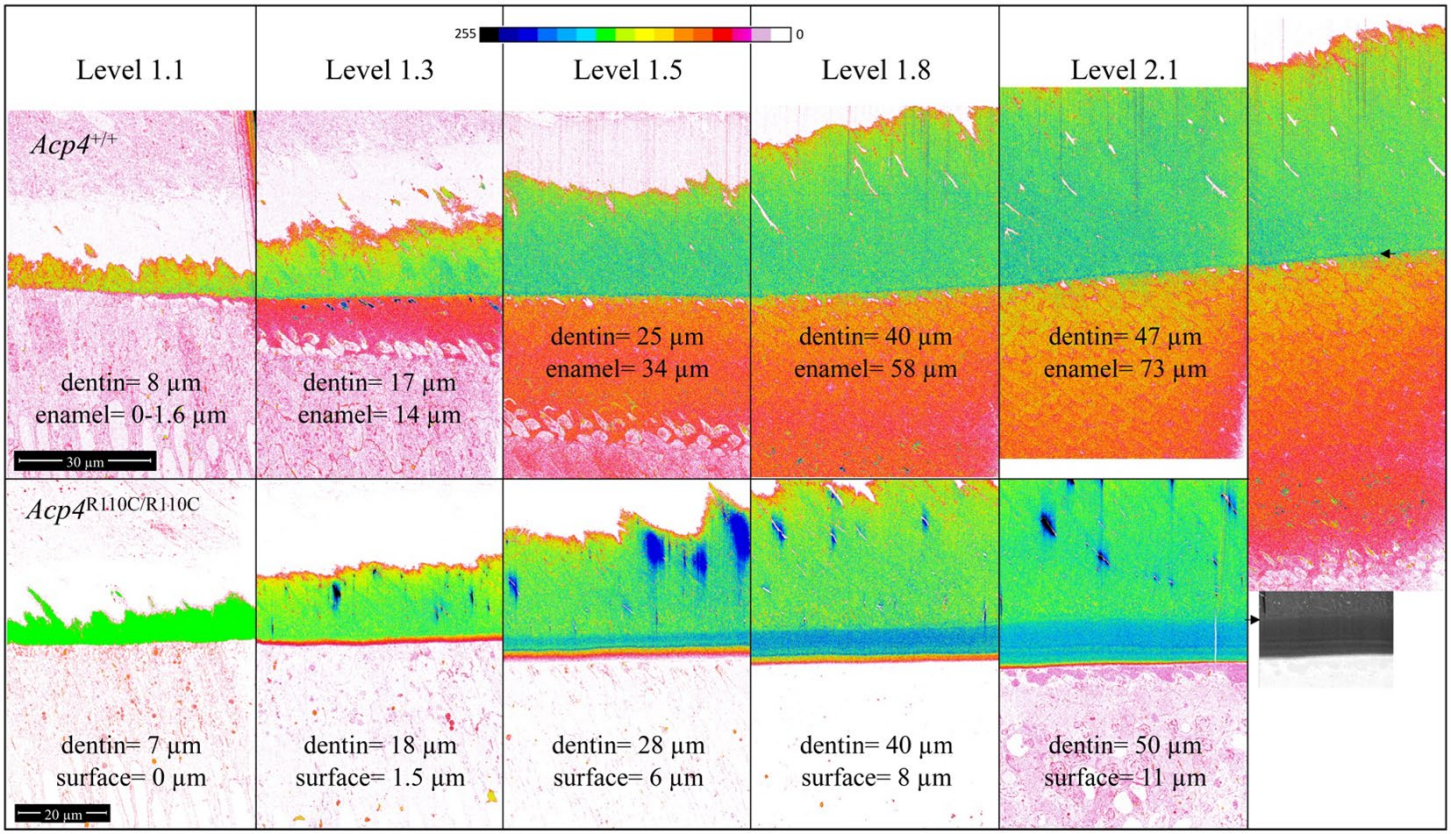
Pseudocolored FIB-bSEM Images of the DEJ Region in *Acp4*^+/+^ and *Acp4*^R110C/R110C^ Mandibular Incisors. Images of various sites along DEJ from Level 1.1 (start enamel formation) to Level 2.1 (mid secretory stage) are presented (all at 1500×). The image in the righthand panel shows the full thickness of dentin and enamel layers in *Acp4*^+/+^ mice at Level 2.1. Beneath it is grey scale image illustrating the difficulty of trying to decide where the DEJ is located because of multiple variations in image intensity across the aplastic enamel layer. Measurements of dentin thickness in the pseudocolored images provides a direct method to delineate this boundary. These measurements provide evidence that dentin formation appears normal within the *Acp4*^R110C/R110C^ mice and that loss of ACP4 function leads to production of little more than a dysfunctional initial enamel layer on the surfaces of mutant teeth. Inverted backscatter intensity pseudo color ramp is shown at the top of the figure.

A comprehensive study of enamel ribbon formation in the *Acp4*^R110C/R110C^ mice using FIB-bSEM is provided in Supplemental data Figs. S22–S27. This study starts at Level 0.673, well before the onset of enamel formation. It continues uninterrupted until level 1.33, jumps to cover Level 1.58–1.70, and ends at Level 1.83 when the ameloblasts undergo cell pathology. FIB-bSEM imaging of mandibular incisors from *Acp4*^+/+^ and *Acp4*^R110C/R110C^ mice at various locations along the sagittal plane from just prior to the onset of mineralization in dentin to around the middle of the secretory stage (Fig. 9) indicated that events leading to the inability of ameloblasts to form a normal enamel layer in *Acp4*^R110C/R110C^ mice begin immediately upon initiation of enamel secretion. Predentin appears to develop and to undergo initiation of mineralization normally (Fig. 9A,F) but the sites where enamel ribbons first appear are more widely spaced and develop in relation to a DEJ area that has more spaces interspersed with pools of organic material (Fig. 9B,G). The distal ends of ameloblasts also appear somewhat expanded in height and studded with small microvillar-like projections (Fig. 9G). The ameloblasts in *Acp4*^R110C/R110C^ mice appear to remain in a static configuration across the interval of time when wild-type ameloblasts move away from the DEJ to form the initial enamel layer followed by elongation of the prongs of interrod enamel needed to map out the territories for the Tomes processes (Fig. 9C,H). As wild-type ameloblasts continue to thicken the enamel layer by appositional growth of the inner enamel layer (Fig. 9D,E/I, *Acp4*^+/+^), ameloblasts in *Acp4*^R110C/R110C^ mice show some signs of moving away from the DEJ leaving behind a very thin layer of mineralizing material containing thin needle-like crystals oriented normal to the DEJ (Fig. 9D,E/I). A small space containing dispersed globs of protein rich material separates this aplastic layer from the distal end of ameloblasts that continues to show microvillar-like projections (Fig. 9E/I). The aplastic enamel layer in *Acp4*^R110C/R110C^ mice continues to increase slowly in thickness to a point in time that is about halfway through the secretory stage (Figs. 9I, 10A–C). Across this interval of time, a much thicker layer of organic material collects at the surface of the mineralized portion of the aplastic enamel layer and the distal ends of ameloblasts develop much larger and widely dispersed villus-like projections that contact the surface of the mineralized portion (Fig. 10B,C). In addition, over time the outermost region of the aplastic mineralized layer shows a more irregular and loose arrangement of widely spaced needle-like mineral crystals studded with small mineral foci while the inner regions become hypermineralized obscuring the DEJ area (Fig. 10C; panel A is for comparison of the appearance of the DEJ at the same location in *Acp4*^+/+^ mice). The inability to delineate the DEJ prompted us to employ pseudocoloring of FIB-bSEM images in order to measure the thicknesses of dentin and enamel in *Acp4*^+/+^ and *Acp4*^R110C/R110C^ mice (Fig. 11). Results from these measurements indicated that dentin appears to maintain normal thickness in *Acp4*^R110C/R110C^ mice but a severe aplasia occurs in the dysfunctional enamel layer that is abnormal in structure (thin and laminated in nature) and achieves only about 10% of the full thickness expected for wild type mice (Fig. 11).

## Discussion

In this study, we report the first *ACP4* indel mutation, c.774_775del, causing severe hypoplastic AI. This deletion will shift the reading frame and cause a premature termination codon (TGA) in Exon 9, which would presumably cause the mutant transcript to undergo nonsense mediated decay and make a complete null *ACP4* allele. The other 10 reported disease-causing mutations are all single nucleotide variants that cause nonsynonymous amino acid changes in ACP4. Interestingly, while all these substituted residues are highly conserved throughout ACP4 evolution, the majority of them are not when compared to ACP2 and ACP3, including Arg^76^, Gln^117^, Ala^128^, Glu^133^, Ser^238^, and Pro^249^ (Supplemental data Fig. S1). This lack of conservation among paralogs suggests that these residues might be critical to the substrate specificity of individual ACPs rather than the general enzymatic function of the phosphatase^51^. Recently, p.Ser238Leu mutant ACP4, which was identified in Family 2 and the other 2 reported families, was shown to retain most of its phosphatase activity in vitro^52^, which supports this hypothesis. A lack of reported complete loss-of-function mutations suggests the amorphic condition might be lethal, especially since *ACP4* is thought to be expressed in many tissues^28,53^. According to HPA^54,55^, and FANTOM5^56^ transcriptomics datasets, expression of *ACP4* can be detected in testis, skin, bone marrow, parathyroid gland, blood, pancreas, and brain tissues. However, the *ACP4* null allele, c.774_775del, we report here strongly indicates that human *ACP4* is only critical for tooth development, as none of the affected individuals in Family 1 showed clinically-detectable nondental abnormalities. This notion is further supported by a recent finding that *ACP4* is inactivated in multiple lineages of toothless or enamelless mammals^36,37^. Therefore, it can be reasonably concluded that *ACP4*, while expressed in nondental tissues, serves critical functions only in teeth.

Acid phosphatase activity has long been detected in developing teeth^33,57–59^. While originally thought to be mainly confined to lysosomes, extra-lysosomal activity was later identified in Golgi apparatus and related organelles of extracellular matrix-forming cells during odontogenesis and osteogenesis^60,61^. Using sodium α-naphthyl acid phosphate as the substrate to detect acid phosphatase activity in developing rat molars, Hammarström et al*.* showed an intense reaction primarily in the Golgi zone, and Tomes’ processes of secretory stage ameloblasts^57^. While the activity was also strongly detected in both stratum intermedium and odontoblasts, it was rather low in maturation stage ameloblasts. The acid phosphatase activity in these developing tissues was distinct from that of osteoclasts in its resistance to fluoride. Particularly, it was demonstrated that the ameloblast reaction was specifically inhibited by sodium molybdate, suggesting a unique acid phosphatase in secretory stage ameloblasts^57^. Several ultrastructural studies further revealed that acid phosphatase activity could be detected in lysosomes, Golgi apparatus and GERL of distal cytoplasm; secretory granules and plasma membrane surface of Tomes’ process; and infranuclear membranes^31,61,62^. All these findings of traditional histocytochemistry are consistent with the results of ACP4 immunohistostaining in developing mouse teeth. In secretory stage ameloblasts, the strongest reactivity was detected at Tomes’ process and a light cytoplasmic reaction presumably in secretory pathway organelles. Little, if any, signal was seen in stratum intermedium and odontoblasts, suggesting that an acid phosphatase other than ACP4 gives rise to the activity detected with traditional histocytochemistry in these cells^57,59^. Based upon the expression pattern of ACP4, we could therefore hypothesize that ACP4 is the fluoride and copper resistant, molybdate and tartrate sensitive acid phosphatase described in the literature. Furthermore, the ACP4 immunostaining also suggests that ACP4, unlike its closest paralog ACP2, is probably not mainly located in the lysosome. This finding is supported by the lack of a lysosomal sorting signal in its cytoplasmic tail. Both ACP2 and ACP3 have a consensus “YXXØ” motif in the C-terminal domain, which is recognized by the AP-2 adaptor protein complex for sorting of transmembrane proteins to endosomes and lysosomes^63–65^. This tyrosine-containing motif on ACP2 has been shown to be necessary and sufficient for its targeting to lysosomes^63^. However, all ACP4 orthologs do not have the critical tyrosine of “YXXØ” motif or other known lysosome sorting signals, suggesting that ACP4 is primarily not a lysosomal protein. Moreover, in the *Acp4*^R110^^C/R110C^ mouse incisor, secretory stage ameloblasts do not show significantly enlarged lysosomes, which are commonly seen in specific cells of mice lacking one of the lysosomal acid phosphatases, including ACP2, ACP3, and ACP5^66,67^. Collectively, all these pieces of evidence indicate that ACP4 probably does not function as a lysosomal degradative enzyme during enamel formation.

The *Acp4* mutant mice (*Acp4*^R110C/R110C^) we generated in this study successfully recapitulate the hypoplastic enamel defects in human *ACP4*-associated AI. Only a thin layer of aplastic enamel, with a certain degree of maturation, is formed on the mutant incisors and molars, indicating that ACP4 plays a critical role in appositional growth of dental enamel formation. Ultrastructurally, the formation of enamel ribbons on dentin mineral is sporadic and delayed, and arrests following very slow formation of initial enamel mineral ribbons, with no subsequent formation of an ameloblast Tomes' process or rod/interrod enamel. Instead, numerous thin needle-like crystals form, mature, and eventually give rise to the thin layer of aplastic enamel. These abnormalities might result from an aberrant secretion or turnover of enamel matrix proteins (EMPs), including ENAM, AMBN, and AMEL. Specifically, a possible reduction of ENAM and AMBN detected in the Tomes’ processes of mutant ameloblasts suggests that these proteins might not be able to function properly along the secretory surface of ameloblast cell membrane, and therefore initiation of enamel mineralization is affected. On the other hand, an increased amount of both intracellular and extracellular AMEL found in the mutant incisor might impede elongation and proper orientation of mineral ribbons. Interestingly, the numerous ectopic mineral depositions in the *Acp4* mutant incisors and molars appear similar to those found in the *Enam* and *Ambn* null mice, although to a lesser extent^45,68,69^. Along with the finding that ACP4 localizes to the Tomes’ process, the above observations suggest that ACP4 is essential for both initiation and elongation of enamel mineral ribbons probably through regulating secretion or turnover of EMPs around mineralization front apparatus^69,70^.

At a molecular level, how ACP4, as an acid phosphatase, functions during appositional growth of enamel formation remains to be elucidated. Whereas other ACPs have been shown to dephosphorylate various molecules in vitro, identifying in vivo substrates for these ACPs has proven difficult^27^. ACP2 and ACP5 mediate dephosphorylation of Man6P (mannose-6-phosphate)-containing proteins, a critical step for activation of lysosomal enzymes^71^. *Acp2* and *Acp5* deficient mice show accumulated unesterified cholesterol in the lysosomes of hepatocytes. The transmembrane isoform of ACP3 functions extra-lysosomally to dephosphorylate extracellular adenosine monophosphate (AMP) to adenosine, which activates A1-adenosine receptor to suppress nociception^72^. ACP5, the most abundant tartrate-resistant acid phosphatase in osteoclasts, dephosphorylates osteopontin (OPN)^73^. *Acp5*-deficient mice exhibit disrupted endochondral ossification and mild osteopetrosis^74^. As the active sites of dimeric ACP enzymes lie in open clefts residing at the interface of distant subunits in the protein's primary structure, a variety of substrates, from large peptides to small molecules, can be possibly processed^27^. Previously, it was proposed that ACP4 could function as a tyrosine phosphatase to dephosphorylate the ErbB4 receptor and might play a role in neuronal differentiation and synaptogenesis^53^. However, this is unlikely because there is no mechanistic explanation as to how the “extracellular” enzymatic domain of ACP4 dephosphorylates the “intracellular” phosphotyrosines of ErbB4 receptor. Therefore, at this stage, the biological substrates of ACP4 remain to be discerned. It is reasonable to hypothesize that ACP4 might dephosphorylate EMPs to regulate their secretion and turnover. However, it is also possible that ACP4 could process signaling molecules that are critical for ameloblast function. It is generally believed that during secretory stage, the extracellular pH values range around neutral conditions (~ 7.2), which might not be optimal for the enzymatic activity of acid phosphatase. Nevertheless, as protons are released when hydroxyapatite crystals form, it is possible that the pH value can drop locally, particularly close to the ameloblast distal membrane where crystallization initiates. This localized, probably transient, acidic environment allows ACP4 to function. Correspondingly, its activity might be controlled in a pH-dependent manner.

Finding that ACP4 localizes to the lateral membrane of secretory stage ameloblasts is interesting. Immunogold staining has localized enamel matrix proteins laterally between ameloblasts^17^, especially basolaterally^75^, indicating that some enamel protein secretion occurs at sites other than the usual rod and interrod secretion sites^4^. Further investigations are warranted to study the molecular functions of ACP4 during enamel formation.

Recently, a spontaneous AI-affected dog breed of Akita was found to carry a homozygous *ACP4* 1-bp duplication in Exon 11, XM_541473.2:c.1189dup^26^. This mutation is predicted to cause a frameshift, p.(Ala397fs), which would delete the C-terminal 31 amino acids of dog ACP4 and predictedly acquire extraneous 91 amino acids due to loss of the natural termination codon. The mutant ACP4 would have an intact extracellular domain with presumably preserved enzymatic activity but lose both the transmembrane domain and the cytoplasmic tail. Interestingly, this mutation appeared to be a hypomorph rather than an amorph, since the affected teeth of the AI dog showed hypoplastic enamel with around 50% of normal thickness^26^. This finding suggested that the mutant dog ACP4, while lacking transmembrane nature and therefore likely to be secreted, preserves its partial function during amelogenesis and thus causes a less severe enamel phenotype compared to human *ACP4*-associated AI and the *Acp4*^R110^^C^ mouse model. This hypothesis is supported by the finding that an *Acp4* transcript expressed by EOE of developing mouse molars encodes a potential secreted form of ACP4s, although the functional significance of this isoform in enamel formation requires further investigations. The mouse model we generated here is an *Acp4* p.Arg110Cys missense variant knock-in mutant, which recapitulates human AI caused by p.Arg111Cys mutation^23^. Based upon the crystal structure of ACP3, this extremely-conserved Arginine (Arg^111^ on human ACP3) resides at the active site of the enzyme and serves as a critical hydrogen bond donor^76^. Therefore, the substitution with a Cysteine should theoretically abolish the acid phosphatase activity of ACP4 in the *Acp4*^R110C^ mouse model. However, it remains as a possibility that this *Acp4*^R110C^ mutant allele is hypomorphic (partial loss of function) rather than amorphic (null). An even more severe enamel phenotype might be expected when ACP4 is completely depleted.

## Materials and methods

### Human subjects

The study protocol and subject consent forms were reviewed and approved by the Ethics Committee at the University of Istanbul and the Institution Review Boards at the University of Michigan. Informed consent was obtained from all participants and/or their legal guardians. Study explanation, pedigree construction, subject enrollment, clinical examinations, and collection of saliva samples were completed under the proper consenting procedure specified in the study protocols and according to the Declaration of Helsinki.

### Laboratory animals

The animal study protocol was reviewed and approved by the Institutional Animal Care and Use Committee at the University of Michigan and complied with US National Research Council's Guide for the Care and Use of Laboratory Animals, the US Public Health Service's Policy on Humane Care and Use of Laboratory Animals, and Guide for the Care and Use of Laboratory Animals^77^. All experiments were performed in accordance with relevant guidelines and regulations and the authors complied with the ARRIVE (Animal Research: Reporting of In Vivo Experiments) guidelines.

### Whole‐exome sequencing and bioinformatic analyses

Subject sample preparation, whole exome sequencing, variant annotation, and mutational analyses were conducted based on established protocols^78^. In brief, variant calling software was used to list DNA sequences that differ from the reference sequences. Searching for genetic variations within a list of genes associated with inherited enamel malformations (Supplemental data Table S2) initially identified the *ACP4* mutations reported here, which were further confirmed by Sanger sequencing.

### Segregation analyses using Sanger sequencing

PCR amplification of *ACP4* exon 7 followed by Sanger sequencing for recruited family members was performed to determine segregation of the identified sequence variant with the disease phenotype. The PCR primers (F: 5′GGCTCCGTTTCAAAACAAAA; R: 5′AAATCGCTGTCACCCTCATC) were used to generate a 530 bp amplification product. Each PCR reaction contained 20 µL of Platinum Hot Start PCR Master Mix (2×) (Invitrogen, Carlsbad, CA, USA), 2 µL of 10 µM primer mix, 1–3 µL of DNA template (final conc. < 500 ng/rxn) and raised to 40 µL with distilled water. The reactions were run using a GeneAmp PCR System 9700 Thermocycler (Applied Biosystems, Foster City, CA, USA). PCR conditions were 94 °C for 2 min, then [30 cycles of 94 °C for 30 s, then 59 °C for 30 s followed by 72 °C for 40 s], 72 °C for 2 min and then hold at 4 °C. PCR products were purified using QIAquick PCR Purification Kit (Qiagen, Hilden, Germany) and sent for Sanger sequencing at the U-M Advanced Genomics Core.

### Analyses of *Acp4* transcript variant expression by mouse EOE

RNA was isolated from enamel organ epithelia (EOE) dissected from first molars of wild-type mice at D5 (secretory stage) and D11 (maturation stage) using the RNeasy Mini Kit (Qiagen, Hilden, Germany), converted to cDNA by the Invitrogen SuperScript III First‐Strand Synthesis System (Invitrogen, Waltham, MA, USA), and PCR amplified using Platinum Hot Start PCR Master Mix (2×) (Invitrogen, Waltham, MA, USA). *Acp4* transcript variant-specific primers and *Gapdh* specific primers are shown in Supplemental data Fig. S3. The RT-PCR amplification products were visualized on a 1.5% agarose gel stained with ethidium bromide (Supplemental data Fig. S3). The PCR conditions were 94 °C for 2 min, then 25 cycles of [94 °C for 30 s, 58 °C for 30 s] followed by 72 °C for 100 s. Each reaction contained 10 µL of Platinum Hot Start PCR Master Mix (2×) (Invitrogen, Carlsbad, CA, USA), 1 µL of 10 µM primer mix, 2 µL of cDNA template and raised to 20 µL with distilled water. The PCR products were purified and analyzed by Sanger sequencing.

### Generation of *Acp4*^R110C^ knockin mouse model

CRISPR/Cas9 technology was used to generate a mouse strain carrying the *Acp4* gene modified by introduction of a cysteine codon (TGC) to replace arginine codon (CGG) at position 110. The *Acp4* mouse model was produced by the University of Michigan Transgenic Animal Core. Mouse Acid Phosphatase 4 (*Acp4*), formerly acid phosphatase, testicular (*Acpt*), coding sequence (Chromosome 7: 44,252,148–44,257,378) was analyzed, and DNA oligo donor and guide RNA sequences designed. The targeting strategy was to introduce a codon change resulting in p.Arg110Cys substitution. The oligonucleotide used for homology directed repair also introduced new restriction sites absent from the wild-type gene. Other silent coding changes were introduced to block Cas9 cleavage of the correctly targeted gene (Supplemental data Fig. S7).

Mouse *Acp4* sequence (chr7:44,253,333–44,253,424) was analyzed to design the single guide RNA (sgRNA). To test the in vitro activity, sgRNA CACAGACTTTGACCGGACATTGG was electroporated with PGKpuro into mouse ES cells, and the cells were subjected to transient puromycin selection. Genomic DNA was prepared from the surviving cells, and a DNA fragment spanning the expected Cas9 cut sites amplified using PCR. Following the confirmation of sgRNA activity, one round of microinjection of 250 eggs from (C57BL/6xSJL)x(C57BL/6xSJL) produced 67 viable offspring. Genotyping was conducted using two PCR primer sets: *Acp4*-WT forward 5′-TTTGACCGGACATTGGAGAG with *Acp4*-WT reverse 5′-TTCCCCTCTCCAGCTCAGTA (408 bp product), and *Acp4*-mutant forward 5′-CACAGACTTCGATTGCACACT with *Acp4*-mutant reverse 5′-ACTCTCCCAGTTTCCCCTGT (629 bp product). The PCR conditions were 94˚C 5:00, followed by (94 °C 0:30, 58 °C 0:30, 72 °C 1:00) for 35 cycles, 72 °C 5:00, and 4 °C hold. Genotyping identified 6 males and 10 females positive for the targeted mutation. Chimeras were bred to produce *Acp4* homozygous mutants. Twelve out of sixteen chimeras demonstrated germline transmission. Once dental phenotypes in the homozygous *Acp4* mutants were observed, genomic DNA from 6 of the best breeders was subjected to Sanger sequence to determine *Acp4* coding sequence integrity.

### Germline transmission

Selected breeders (G0) with properly targeted sequence changes were crossed with C57BL/6N mice (Charles River, strain code 027) for seven generations then sib-pair breeding was conducted for sample collections. All G0 founders demonstrated germline transmission. No behavior changes or physical defects from neonatal stage to young adulthood were detected in the founders or their *Acp4*^+/R110C^ offspring. Genotyping and sequence validation were conducted as described in Supplemental data Fig. S8.

### Dissection microscopy

Frontal facial images were taken of 7-week *Acp4*^+/+^, *Acp4*^+/R110C^, and *Acp4*^R110C/R110C^ mice lightly anesthetized with isoflurane using the Nikon SMZ1000 dissection microscope with a Nikon DXM1200 digital camera. Three samples from each genotype were evaluated. Separately, another group of mice were deeply anesthetized with isoflurane and perfused with 1× phosphate buffered saline (PBS) for 10 min. Mandibles were dissected out and cleaned of soft tissues, post-fixed by immersion in 4% paraformaldehyde (PFA) overnight, then rinsed with PBS three times for 5 min each. Hemimandibles were cleaned with 1% bleach (sodium hypochlorite), rinsed with PBS, air dried, displayed on the dissection microscope, and photographed, as described previously^50^.

### Histological analysis

*Acp4*^+/+^, *Acp4*^+/R110C^, and *Acp4*^R110C/R110C^ mice at postnatal Days 5, 8 and 12 were harvested, and their heads dissected into halves. Separately, hemimandibles of D12 mice were dissected out from the heads. Samples were fixed in 4% paraformaldehyde in diethyl pyrocarbonate (DEPC)-treated phosphate buffered saline (PBS; 137 mM NaCl, 2.7 mM KCl, and 11.9 mM phosphates) at 4 °C for ~ 18 h. The samples were decalcified in DEPC-treated 4.13% EDTA, pH 7.4, with agitation at 4 °C. The EDTA solution was changed every 3 days for 5 to 12 days, depending on the age of the mice where the samples were harvested. The tissues were then dehydrated and embedded in paraffin. Longitudinal sections (5 µm) were prepared for Hematoxylin and Eosin (H&E) staining as previously described^50^. Sections were inspected under a Nikon Eclipse TE300 microscope and photographed using a Nikon DXM1200 digital camera.

### In situ hybridization

Longitudinal sections prepared for histological analysis were further used for in situ hybridization. An antisense *Acp4* riboprobe for RNAscope in situ hybridization was designed and produced by Advanced Cell Diagnostics (Newark, CA, USA). RNAscope^®^ 2.5 Assay with RED HD Detection Reagent (Advanced Cell Diagnostics, Inc. Newark, CA, USA) was performed following the user manuals 322452 (FFPE sample preparation and pretreatment) and 322360 (RNAscope^®^ 2.5 HD Detection Reagent—RED user manual) as described previously^46,79^. The following probes were used: (1) Mm-Acp4 (Cat #590761, targeting NM_001195034.1, nt 215–1091) and (2) Neg Ctrl Probe_dapB (Cat #310043). Photographs were taken using a Nikon DXM1200 digital camera connected to a Nikon Eclipse TE300 microscope.

### Immunohistochemistry

Paraffin sections were heated at 60 °C for 1 h, rehydrated in xylene and an ethanol series. Antigen retrieval was performed in Tris–EDTA buffer (10 mM Tris Base, 1 mM EDTA, 0.05% Tween 20, pH 9.0). The antigen retrieval container was placed in boiling water for 22 min, followed by cooling down at room temperature (RT) for 22 min. Sections were then blocked in 3% bovine serum albumin, 10% normal goat or donkey serum, 0.05% Tween 20 in PBS at RT for 2 h, and incubated in an unconjugated primary antibody in antibody dilution buffer (10% normal goat or donkey serum, 0.05% Tween 20 in PBS) at RT for 1 h, then at 4 °C overnight. The unconjugated primary antibodies used in this study were (1) ACP4 rabbit polyclonal antibody (Biorbyt, orb101887), (2) LAMP1 rat monoclonal IgG2a antibody (DSHB, 1D4B-c), (3) RAB5 rabbit monoclonal (C8B1) antibody (Cell Signaling Technology, #3547), (4) CLTC/clathrin heavy chain rabbit monoclonal [EPR12235(B)] antibody (Abcam, ab172958), (5) AMEL rabbit polyclonal antibody (rM179)^80^, (6) ENAM rabbit polyclonal antibody (Ms #223–236)^81^, and (7) AMBN rabbit polyclonal antibody (Pig #89–102)^82^. After a thorough wash in 0.05% Tween 20 in PBS, secondary antibodies were incubated at RT for 1 h. Secondary antibodies used in this study were: (1) Goat anti‐rabbit IgG(H + L) Secondary Antibody, Alexa Fluor Plus 555 (Invitrogen, A32732), (2) Donkey anti-rabbit IgG(H + L) Secondary Antibody, Alexa Fluor Plus 647 (Invitrogen, A32795), and (3) Donkey anti-rat IgG(H + L) Secondary Antibody, Alexa Fluor Plus 488 (Invitrogen, A48269). To delineate the Tomes’ process and distal and proximal terminal webs, a mouse monoclonal (AC-15) antibody against β-actin with an FITC conjugate (Abcam, ab6277) was incubated for 1 h at RT. The section was counterstained in 4′,6-Diamidino-2-phenylindole dihydrochloride (DAPI, 1 μg/mL in distilled water; D9542, Sigma) for 2 min, then mounted in Invitrogen ProLong Gold Antifade Mountant with DAPI (P36935, Invitrogen, Carlsbad, CA). Some images (Fig. 4, Supplemental data Figs. S10–S14) were taken using a Leica STELLARIS 8 confocal system equipped with a White Light Laser and 5 Power HyD Detectors. Others (Supplemental data Figs. S18–S21) were taken using a Nikon A1 confocal combined with an inverted Ti-E microscope. Both confocal microscopes were at the Imaging Laboratory of the Michigan Diabetes Research Center (Ann Arbor, MI).

### Backscattered scanning electron microscopy (bSEM)

Topographic scanning of the tooth surface was performed on D14 and 7-week mandibular molars of *Acp4*^+/+^, *Acp4*^+/R110C^, and *Acp4*^R110C/R110C^ mice. The hemimandibles were submerged in 4% PFA overnight and carefully dissected of soft tissues. On the following day, samples were submerged in 1% NaOCl for 20 min, rinsed, dehydrated using an acetone series (30, 50, 70, 80, 90, and 100%), and air dried before mounted on metallic stubs using conductive carbon cement. Images were taken using a Joel JSM-7800FLV field-emission scanning electron microscope operating at an accelerating voltage of 15 kV in the backscatter mode at the University of Michigan Robert B. Mitchell Electron Microbeam Analysis Lab (EMAL, Ann Arbor, MI, USA).

For bSEM cross-sectional analyses of mouse mandibular incisors, the teeth were prepared following an established protocol^83^. Seven‐week‐old *Acp4*^+/+^, *Acp4*^+/R110C^, and *Acp4*^R110C/R110C^ mice were anesthetized with isoflurane, perfused with 4% PFA, and their hemimandibles dissected free of soft tissue, dehydrated with an acetone series (30, 50, 70, 80, 90, and 100%), embedded in epoxy, and cross sectioned at 1 mm increments along their lengths as described previously^44,84^. The cut surface was polished and coated with carbon to increase conductivity and examined at ×5000 magnification in Joel 7800 (JEOL USA, Inc., Peabody, MA, USA) using the backscatter mode at a beam current of 20 kV and 10 nA. Images were taken at a working distance of 10 mm, with minor adjustments to focus. With ImageJ (http://rsb.info.nih.gov/ij/), selected images were normalized to have the same mean grayscale intensities for mineralized dentin so that the grayscale of bSEM images from different samples could be accurately compared for the degree of mineralization.

### Focused ion beam scanning electron microscopy (FIB‑bSEM)

Mandibular incisors of *Acp4*^+/+^ and *Acp4*^R110C/R110C^ mice were fixed and embedded in epoxy plastic as described previously^2^. The specimen faces in the polymerized blocks were smoothed with glass knives, and the blocks attached to 45° chamfered mounting stubs with conductive silver paste. The stubs were placed in the imaging chamber of a Helios Nanolab 660 FIB-SEM (FEI, Systems for Research, Longueuil, QC, Canada). A sampling area 100 μm × 100 μm in size was selected and milled with gallium ions at rough (45 nA) followed by fine (9.4 nA) settings. Imaging was performed with the through lens detector (TLD) and where possible with the in-column detector (ICD). All FIB-bSEM imaging was done using blocks prepared for sagittal (longitudinal) views of the enamel layer and associated enamel organ cells. Where possible, blocks from 3 different mice per genotype were examined. Charging on the surfaces of block faces was reduced by coating them with a thin layer of platinum (3 nm) where required. Curtaining defects in TLD images were reduced using python language software described previously^85^. In an attempt to better define the DEJ area in *Acp4*^R110C/R110C^ mutant mice, images from *Acp4*^+/+^ and *Acp4*^R110C/R110C^ mice at sequential levels of section across the first half of the secretory stage were normalized in ImageJ (https://imagej.nih.gov/ij/) based on mean dentin intensity and then pseudocolored using the built in 16 color lookup table (LUT) (Fig. 11). The images were scaled to magnification markers embedded on each image, and the thickness of dentin and enamel or aplastic enamel in the case of *Acp4*^R110C/R110C^ mutant mice measured and recorded.

## Supplementary Information

Supplementary Information 1.

Supplementary Information 2.

Supplementary Information 3.

## Data Availability

The WES data from this study can be accessed at Genetics of Disorders Affecting Tooth Structure, Number, Morphology and Eruption (dbGaP Study Accession: phs001491.v2.p1). Data dictionaries and variable summaries are available on the dbGaP FTP site: https://ftp.ncbi.nlm.nih.gov/dbgap/studies/phs001491/phs001491.v2.p1 (accessed on 11 November 2019). The mouse strain, 067187-UNC or B6N.Cg-Acp4em1Jcch/Mmnc, is available at the MMRRC-UNC facility.
