# Supplementary material for: Enamel defects in Acp4R110C/R110C mice and human ACP4 mutations
Source: Sci Rep. 2022 Oct 1;12:16477. doi: 10.1038/s41598-022-20684-9 (PMC9526733; doi:10.1038/s41598-022-20684-9)
Supplement: Supplementary file 1 — Supplementary Information 1. [file 41598_2022_20684_MOESM1_ESM.pdf]

Enamel Defects in *Acp4*$^{R110C/R110C}$ Mice and Human *ACP4* Mutations

Tian Liang[1], Shih-Kai Wang[2,3], Charles Smith[1,4], Hong Zhang[1], Yuanyuan Hu[1], Figen Seymen[5], Mine Koruyucu[6], Yelda Kasimoglu[6], Jung-Wook Kim[7,8], Chuhua Zhang[1], Thomas L. Saunders[9], James P. Simmer[1]*, and Jan C-C. Hu[1]

*There were equal contributions from Tian Liang and Shih-Kai Wang, and both should be considered to be first authors.

Affiliations

[1]Department of Biologic and Materials Sciences, University of Michigan School of Dentistry, 1210 Eisenhower Place, Ann Arbor, MI 48108, USA.

[2]Department of Dentistry, National Taiwan University School of Dentistry, No. 1, Changde St., Zhongzheng Dist., Taipei City 100, Taiwan.

[3]Department of Pediatric Dentistry, National Taiwan University Children's Hospital, No. 8, Zhongshan S. Rd., Zhongzheng Dist., Taipei City 100, Taiwan.

[4]Department of Anatomy & Cell Biology, Faculty of Medicine & Health Sciences, McGill University, Montreal, QC, Canada

[5]Department of Pedodontics, Faculty of Dentistry, Altinbas University, Istanbul, 34147, Turkey.

[6]Department of Pedodontics, Faculty of Dentistry, Istanbul University, Istanbul, 34116, Turkey

[7]Department of Molecular Genetics & Dental Research Institute, School of Dentistry, Seoul National University, Seoul 03080, Republic of Korea.

[8]Department of Pediatric Dentistry & Dental Research Institute, School of Dentistry, Seoul National University, Seoul 03080, Republic of Korea.

[9]Department of Internal Medicine, Division of Molecular, Medicine and Genetics, University of Michigan Medical School, Ann Arbor, MI 48109, USA.

Contents of Supplemental Data File 1

Table S1. Known *ACP4* Pathogenic Variants

Table S2. AI Candidate Genes Specifically Targeted during Whole Exome Analyses

Figure S1. Alignment of Histidine Acid Phosphatases.

Figure S2. Analysis of Mouse *Acp4* Transcript Variants.

Figure S3. Analysis of Mouse *Acp4* Transcript Variants Amplified from Enamel Organ Epithelium (EOE) of D5 and D11 First Molars.

Figure S4. Exon Composition of Membrane-Bound Form of Mouse ACP4.

Figure S5. Exon Composition of Potentially Secreted Form of Mouse ACP4.

Figure S6. Partial Intron/Exon Structure of Mouse *Acp4* Gene.

Figure S7. CRISPR/Cas9 Strategy to Generate *Acp4*^{R110C} Knockin Mice.

Figure S8. *Acp4*^{R110C} Genotyping and Validation.

Figure S9. *In Situ* Hybridization of *Acp4* in *Acp4*$^{+/+}$, *Acp4*$^{+/R110C}$, and *Acp4*$^{R110C/R110C}$ Mouse Teeth.

Figure S10: Immunohistochemistry of ACP4 (anti-mouse ACP4 antibody) and LAMP1 in *Acp4*$^{+/+}$ (WT) and *Acp4*$^{R110C/R110C}$ D12 Mandibular Incisors.

Figure S11. Immunohistochemistry of ACP4 (anti-human ACP4 antibody) and LAMP1 in *Acp4*$^{+/+}$ (WT) and *Acp4*$^{R110C/R110C}$ D12 Mandibular Incisors.

Figure S12. Immunohistochemistry of ACP4, LAMP1, RAB5, and CLTC on Ameloblasts of *Acp4*$^{+/+}$ D12 Mandibular Incisors.

Figure S13. Immunohistochemistry of LAMP1 and CLTC in *Acp4*$^{+/+}$ and *Acp4*$^{R110C/R110C}$ D12 Mandibular Incisors.

Figure S14. Immunohistochemistry of LAMP1 and RAB5 in *Acp4*$^{+/+}$ and *Acp4*$^{R110C/R110C}$ D12 Mandibular Incisors.

Figure S15. bSEM Images of *Acp4*$^{R110C/R110C}$ (226) and *Acp4*$^{+/R110C}$ (277) 7-week Mandibular Incisors Cross-sectioned at 1 mm Increments (levels 2 through 8).

Figure S16. bSEM Images of *Acp4*$^{R110C/R110C}$ (230) and *Acp4*$^{+/R110C}$ (281) 7-week Mandibular Incisors Cross-sectioned at 1 mm Increments (levels 2 through 8).

Figure S17. bSEM Images of *Acp4*$^{R110C/R110C}$ (294) and *Acp4*$^{+/R110C}$ (282) 7-week Mandibular Incisors Cross-sectioned at 1 mm Increments (levels 2 through 8).

Figure S18. Immunohistochemistry of AMEL in *Acp4*$^{+/+}$ and *Acp4*$^{R110C/R110C}$ D12 Mandibular Incisors.

Figure S19. Immunohistochemistry of ENAM in *Acp4*$^{+/+}$ and *Acp4*$^{R110C/R110C}$ D12 Mandibular Incisors.

Figure S20. Immunohistochemistry of AMBN in *Acp4*$^{+/+}$ and *Acp4*$^{R110C/R110C}$ D12 Mandibular Incisors.

Figure S21. Immunohistochemistry of ACP4 in *Acp4*$^{+/+}$ and *Acp4*$^{R110C/R110C}$ D5 Maxillary First Molars.

Table S1. Known *ACP4* Pathogenic Variants

Exon	NG_052652.1	NM_033068.3	NP_149059.1	dbSNP	Reference
Exon 3	g.5369C>T	c.226C>T	p.(Arg76Cys)	rs1057519277	[1]
Exon 3	g.5405C>A	c.262C>A	p.(Arg88Ser)	rs1190557090	[2]
Exon 4	g.6269C>T	c.331C>T	p.(Arg111Cys)	rs202073531	[1]
Exon 4	g.6288A>G	c.350A>G	p.(Gln117Arg)	N/A	[2]
Exon 4	g.6320G>C	c.382G>C	p.(Ala128Pro)	rs767907487	[1]
Exon 4	g.6335G>A	c.397G>A	p.(Glu133Lys)	rs779823931	[1]
Exon 4	g.6357C>T	c.419C>T	p.(Pro140Leu)	rs1371134137	[2]
Exon 4	g.6366C>T	c.428C>T	p.(Thr143Met)	rs546603773	[3]
Exon 7	g.8337C>T	c.713C>T	p.(Ser238Leu)	rs763573828	Family 2; [1,2]
Exon 7	g.8370C>T	c.746C>T	p.(Pro249Leu)	rs1085307111	[3]
Exon 7	g.8398_8399del	c.774_775del	p.(Gly260Aspfs*29)	rs768702435	Family 1

1. Seymen, F. *et al.* Recessive Mutations in ACPT, Encoding Testicular Acid Phosphatase, Cause Hypoplastic Amelogenesis Imperfecta. *Am. J. Hum. Genet.* **99,** 1199-1205 (2016).
2. Kim, Y. J. *et al.* Recessive Mutations in ACP4 Cause Amelogenesis Imperfecta. *J Dent Res* **101,** 37-45 (2022).
3. Smith, C. E. *et al.* Defects in the acid phosphatase ACPT cause recessive hypoplastic amelogenesis imperfecta. *Eur J Hum Genet.* **25,** 1015-1019 (2017).

Table S2. AI Candidate Genes Specifically Targeted during Whole Exome Analyses

ACP4	*ADAMTS2*	*AIH3*	*AIRE*	*ALDH3A2*	*ALPL*	*AMBN*
AMELX	*AMTN*	*ARHGAP6*	*ATP6v0d2*	*ATP6v1b2*	*ATP6v1c1*	*ATP6v1e1*
ATR	*AVPR2*	*CACNA1C*	*CELIAC1*	*CLDN1*	*CLDN10*	*CLDN16*
CLDN19	*CNNM4*	*COL17A1*	*COL7A1*	*CREBBP*	*CTBP*	*CTBP1*
CYP27B1	*DLX3*	*DMP1*	*DNAJC21*	*DSC1*	*DSC2*	*DSPP*
ENAM	*EP300*	*ERCC8*	*EVC1*	*EVC2*	*FAM20A*	*FAM20C*
FAM83H	*FBLN2*	*FERMT1*	*FGF23*	*FGF3*	*FGFR10*	*FGFR2*
FGFR3	*FOXC1*	*GALNS*	*GALNT3*	*GJA1*	*GLB1*	*GNAS*
GPR68	*GPR98*	*HSD17B4*	*HSD17B4*	*IRX5*	*ITGA6*	*ITGB4*
ITGB6	*ITPR3*	*KIAA0753*	*KIF1A*	*KIF2*	*KIF21A*	*KIF4A*
KL	*KLK4*	*LAMA3*	*LAMB3*	*LAMC2*	*LAMC3*	*LTBP3*
MBTPS2	*MMP20*	*MTX2*	*NF1*	*NHS*	*OCRL1*	*ODAM*
ODAPH	*OPDZD7*	*ORAI1*	*PDZD7*	*PEX1*	*PEX26*	*PEX6*
PHEX	*PITX2*	*PLEC1*	*PORCN*	*PTDSS1*	*RAC1*	*RAI1*
ROGDI	*RUNX2*	*SATB1*	*SATB2*	*SLC13A5*	*SLC24A4*	*SLC24A5*
SLC34A2	*SLC4A4*	*SP6*	*STIM1*	*TBCE*	*TMEM165*	*TP63*
TSC1	*TSC2*	*VDR*	*WDR72*			

```
hACP4   MAG-LGFWGHPAG--PLLLLLLLVLPPRALPEGPLVFVALVFRHGDRAPLASYPMDPHKEVAST  61
mAcp4   MAE-PGSQGHTVG--PLLLLLLL-LLPRALPEGPLLFVALVFRHGDRAPLASYPTDPHKEAAST  60
hACP2   MAGKRSGWSRAAL--LQLLLGVNLVVMPPTRARSLRFVTLLYRHGDRSPVKTYPKDPYQE---E  59
hACP3   MRAAPLLLARAASLSLGFLFLLFFWLDRSVLAKELKFVTLVFRHGDRSPIDTFPTDPIKE---S  61
        *       .: .     :*: :            * **:*::*****:*: ::* ** :*

hACP4   LWPRGLGQLTTEGVRQQLELGRFLRSRYEAFLSPEYRREEVYIRSTDFDRTLESAQANLAGLFP 125
mAcp4   LWPRGLGQLTKEGIRQQLELGRFLRRRYKAFLSPEYKREEVYIRSTDFDRTLESAQANLAGLFP 124
hACP2   EWPQGFGQLTKEGMLQHWELGQALRQRYHGFLNTSYHRQEVYVRSTDFDRTLMSAEANLAGLFP 123
hACP3   SWPQGFGQLTQLGMEQHYELGEYIRKRYRKFLNESYKHEQVYIRSTDVDRTLMSAMTNLAALFP 125
         **:*:****  *: *: ***. :* **. **. .*::::**:****.**** ** :***.***

hACP4   EAAP--GSPEARWRPIPVHTVPVAEDKLLRFPMRSCPRYHELLREATEAAEYQEALEGWTGFLS 187
mAcp4   EAAP--GSPETDWKPIPVHTVPVSEDKLLRFPMRSCPRYHELLRESTEAADYQEALEGWTDFLT 186
hACP2   PNGMQRFNPNISWQPIPVHTVPITEDRLLKFPLGPCPRYEQLQNETRQTPEYQNESSRNAQFLD 187
hACP3   PEGVSIWNPILLWQPIPVHTVPLSEDQLLYLPFRNCPRFQELESETLKSEEFQKRLHPYKDFIA 189
          .    .*   *:********::**:** :*:  ***:.:*  *: :: ::*:       *:

hACP4   RLENFTGLSLVGEPLRRAWK-VLDTLMCQQAHGLPLPAWASPDVLRTLAQISALDIGAHVGPPR 250
mAcp4   RLGNFTGLSLVGEPLRRAWK-VLDTLICQRAHGLDLPSWASPDVLRTLSQISALDIRAHVGPPR 249
hACP2   MVANETGLT--DLTLETVWN-VYDTLFCEQTHGLRLPPWASPQTMQRLSRLKDFSFRFLFGIYQ 248
hACP3   TLGKLSGLH--GQDLFGIWSKVYDPLYCESVHNFTLPSWATEDTMTKLRELSELSLLSLYGIHK 251
         : : :**   .  *   *. * * * *: .*.: ** **: :.:  * .:. :.:    *  :

hACP4   AAEKAQLTGGILLNAILANFSRVQRLGLPLKMVMYSAHDSTLLALQGALGLYDGHTPPYAACLG 294
mAcp4   AAEKAQLTGGILLDAILSNFSRTQRLGLPLKMVMYSAHDSTLLALQGALGLYDGNTPPYAACMA 293
hACP2   QAEKARLQGGVLLAQIRKNLTLMATTSQLPKLLVYSAHDTTLVALQMALDVYNGEQAPYASCHI 292
hACP3   QKEKSRLQGGVLVNEILNHMKRATQIPSYKKLIMYSAHDTTVSGLQMALDVYNGLLPPYASCHL 295
          **::* **:*:  *  ::.         *:::*****:*: .** **.:*:*   ***:*

hACP4   FEFRKHLGNPA-KDGGNVTVSLFYRNDSAHLPLPLSLPGCPAPCPLGRFYQLTAPARPPAHGVS 377
mAcp4   FEFRGSSREPEEEDGENVTVSLIYRNDTSRPPLPLRVPGCPAPCPLGRFQQLTAPARPPAHGAP 377
hACP2   FELY-------QEDSGNFSVEMYFRNESDKAPWPLSLPGCPHRCPLQDFLRLTEPVVPKDWQQE 369
hACP3   TELY-------FE-KGEYFVEMYYRNETQHEPYPLMLPGCSPSCPLERFAELVGPVIPQDWSTE 371
         *:         :   :  *.: :**:: : * ** :***   ***  *  .*.  *. *

hACP4   CHGPYEAAIPPAPVVPLLAGAVAVLVA---LSLGLGLLAWRPGCLRALGGPV*--- 426
mAcp4   CHGSYEPASPPA-TVPLLAGAVAVLAV---LSLGLGLLAWRPRCLRALGGTV*--- 425
hACP2   CQLASGPADTEVIVALAVCGSILFLLIVLLLTVLF-RMQAQPPGYRHVADGEDHA* 423
hACP3   CMTTNSHQVLKVIFAVAFCLISAVLMVLLFIHIRR-GLCWQRESYGNI*------- 418
        *          .  .  ..    .*     : :    :  :      :
```

Figure S1. Alignment of Histidine Acid Phosphatases. Human and mouse ACP4 protein sequences were aligned to the human ACP2 and ACP3 sequences: hACP4 (NP_149059.1), mAcp4 (NP_001181963.1), hACP2 (NP_001601.1), hACP3 (NP_001127666.1). Human *ACP4* encodes a single-pass type I membrane glycoprotein. Noteworthy structural features of hACP4 are: A signal peptide (green, 1-26); extracellular domain (blue, 27-393) with N-glycosylation target sequences (cyan; N191, N269, N330, and N339), and disulfide bonds (yellow; connecting C159-C378, C214-C312, and C353-C357); transmembrane domain (gray, 394-414); and a cytoplasmic domain (orange, 415-426). The hACP4 structural features were obtained from UniProtKB, see: https://www.uniprot.org/uniprot/Q9BZG2#function. The mouse sequence used to generate a new mACP4 anti-peptide antibody is underlined.

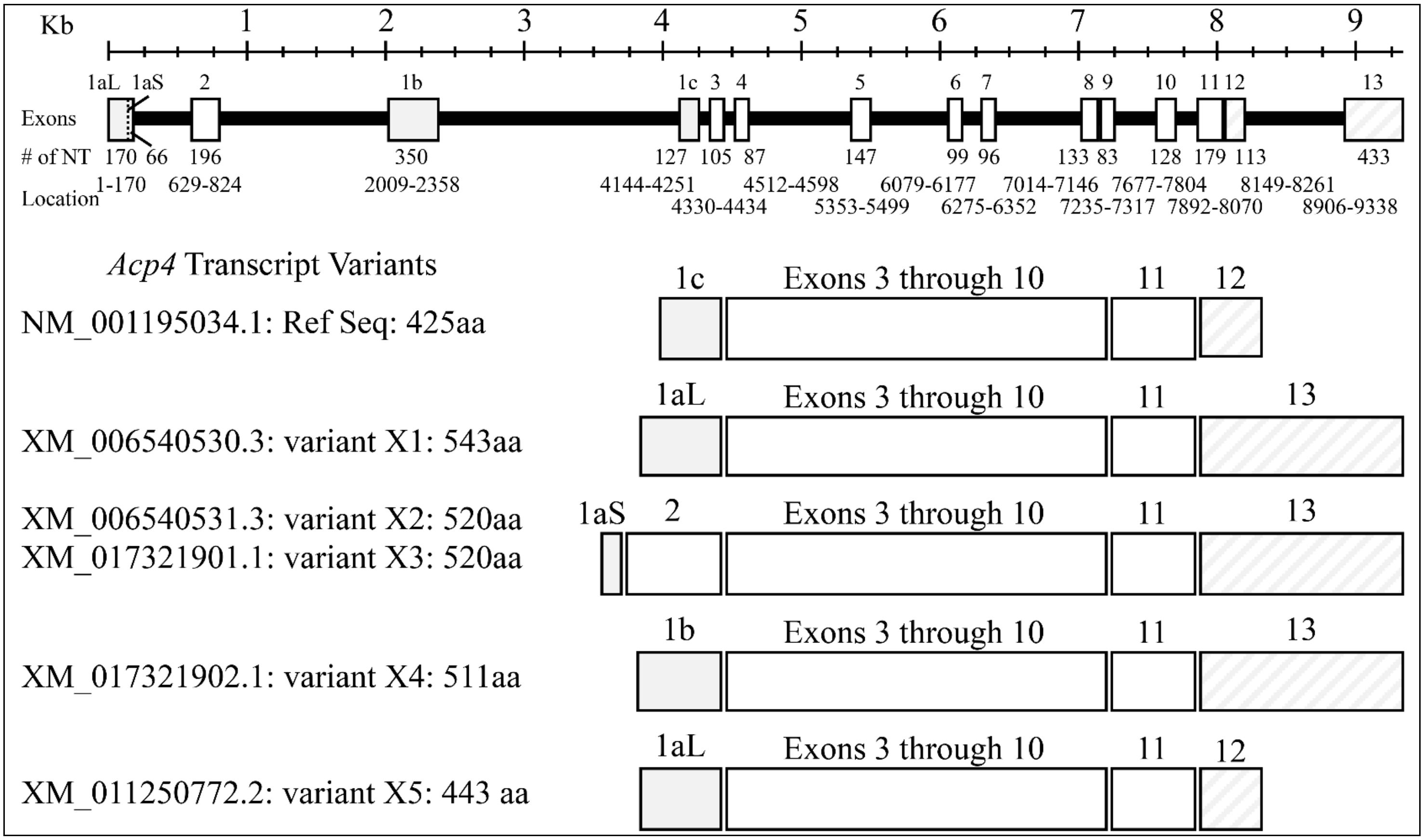

Figure S2. Analysis of Mouse *Acp4* Transcript Variants. The NCBI Reference Sequence (NC_000073.7) for the C57BL/6J mouse *Acp4* gene on chromosome 7 is 9338 bp in length. The scale for this sequence (in Kb) is shown at the top. Below this is a scale model of the mouse *Acp4* exon (boxes) and intron (black line) gene structure. At the time of our initial analysis, there were one reference transcript and five *Acp4* mRNA isoform X variants listed in NCBI. Exons 3 through 11 are found on all transcripts. The X variants have unique starting points at their 5' ends as well as unique translation initiation sites relative to the reference sequence: Exon 1aL (long, 1-170), or Exon 1aS (105-170) that is paired with unique exon 2 (629-824) that contains the translation initiation codon, or Exon 1b (2009-2358). None of these variants produce a recognizable signal peptide and are of dubious function. In NCBI database, only the reference transcript (NM_001195034.1) starts at Exon 1c (4144-4251), which encodes a signal peptide. Exon 12 encodes the transmembrane domain and the cytoplasmic domain. This exon is replaced by Exon 13 in four of the isoform X variants.

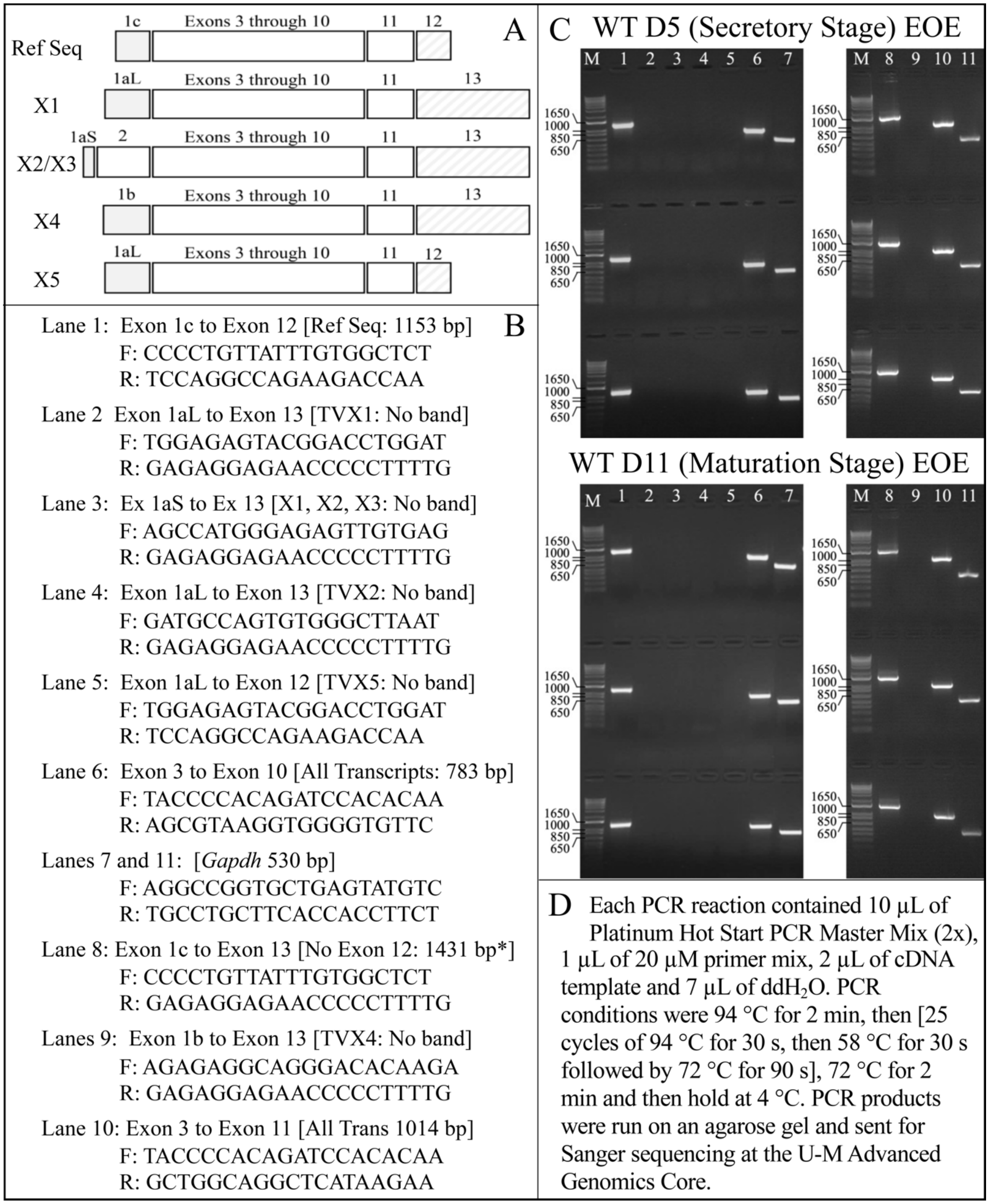

Figure S3. Analysis of Mouse *Acp4* Transcript Variants Amplified from Enamel Organ Epithelium (EOE) of D5 and D11 First Molars. ***A:*** Exon structures of *Acp4* mRNA reference transcript (NM_001195034.1), TVX1 (XM_006540530.5), TVX2 (XM_006540531.4), TVX3 (XM_017321901.2), TVX4 (XM_017321902.3) and TVX5 (XR_003946367.1). ***B:*** Sequences of PCR amplification primers and sizes of the amplification products as determined by Sanger sequencing. ***C:*** Agarose gel electrophoreses showing RT-PCR results from D5 (top) and D11 (bottom) molars. Note that the amplicons from the three D5 (secretory stage) and three D11 (maturation stage) molars gave the same amplification patterns. In mouse EOE only the band in lane 1 gave an amplification product (1153 bp) identical to the mouse *Acp4* reference sequence (NM_001195034.1), which has the same structure as the human *ACP4* reference (NM_033068.3). Lanes 6 and 10 gave single amplification products (783 bp and 1014 bp), demonstrating that exons 3 through 11 are found on all mouse *Acp4* transcript variants expressed in molars. Lanes 7 and 11 both show the *Gapdh* control product (530 bp). Lane 8 gave an amplicon that was identical to the reference except that the 3' exon on the reference sequence (exon 12) was deleted by alternative splicing and replaced with a novel exon 13. Only two *Acp4* transcripts are expressed in mouse EOE, and both start with exon 1c just upstream of exon 3 (Lanes 1 and 8). ***D:*** PCR reaction conditions.

4144..4251, 4330..4434, 4512..4598, 5353..5499, 6079..6177, 6257..6352, 7014..7146, 7235..7317, 7677..7804, 7892..8070, 8149..8261

```
atggctgagcccgggtctcagggccacaccgtcggacccttgctgctgctgctgttgctgttgctgcctcgggccctgccggagggg
 M  A  E  P  G  S  Q  G  H  T  V  G  P  L  L  L  L  L  L  L  L  P  R  A  L  P  E  G
cccctgttatttgtggctctggtgttccgacatggcgaccggccccactggcctcctaccccacagatccacacaaggaagctgcc
 P  L  L  F  V  A  L  V  F  R  H  G  D  R  A  P  L  A  S  Y  P  T  D  P  H  K  E  A  A
tccaccttgtggcctcgaggcttgggccaactgactaaggaggggatccgccagcagctagaactgggccgatttctgaggaggcgt
 S  T  L  W  P  R  G  L  G  Q  L  T  K  E  G  I  R  Q  Q  L  E  L  G  R  F  L  R  R  R
tacaaggctttcctgagccctgagtacaagcgagaagaggtgtacatccgcagcacagactttgaccggacattggagagtgcacaa
 Y  K  A  F  L  S  P  E  Y  K  R  E  E  V  Y  I  R  S  T  D  F  D  R  T  L  E  S  A  Q
gccaacctggctgggctcttccctgaggctgcccctggaagtcctgagactgactggaagcccattccagtgcacacagtgcctgtg
 A  N  L  A  G  L  F  P  E  A  A  P  G  S  P  E  T  D  W  K  P  I  P  V  H  T  V  P  V
tctgaggacaagttgctgaggttccccatgcgcagctgtcctcgataccatgagctgttacgagagtccacagaggcagctgactac
 S  E  D  K  L  L  R  F  P  M  R  S  C  P  R  Y  H  E  L  L  R  E  S  T  E  A  A  D  Y
caggaggccctggagggctggacggacttcctgacccgcctgggcaacttcactgggctgtccctggttggagagccactccggaga
 Q  E  A  L  E  G  W  T  D  F  L  T  R  L  G  N  F  T  G  L  S  L  V  G  E  P  L  R  R
gcatggaaagttctggataccttgatctgccagcgtgcccatggtcttgaccttccatcctgggcctctccagatgtcttgaggact
 A  W  K  V  L  D  T  L  I  C  Q  R  A  H  G  L  D  L  P  S  W  A  S  P  D  V  L  R  T
ctgtcacagatttctgctctggatatcagggcccatgtaggcccaccccgagcagcagaaaaggcccagctgacaggggggatcctg
 L  S  Q  I  S  A  L  D  I  R  A  H  V  G  P  P  R  A  A  E  K  A  Q  L  T  G  G  I  L
ctggatgctattctcagcaatttctcccggacccagcgccttgggttgcccctcaagatggtcatgtactcagctcatgacagcacc
 L  D  A  I  L  S  N  F  S  R  T  Q  R  L  G  L  P  L  K  M  V  M  Y  S  A  H  D  S  T
ctgctggccctccagggggccctgggcctctacgatgggaacaccccaccttacgctgcctgcatggcctttgaattccggggggagc
 L  L  A  L  Q  G  A  L  G  L  Y  D  G  N  T  P  P  Y  A  A  C  M  A  F  E  F  R  G  S
tccagggaacccgaggaggaagatggagagaatgtcactgtctctctcatctaccgcaatgacacctcccgcccacccctgccactc
 S  R  E  P  E  E  E  D  G  E  N  V  T  V  S  L  I  Y  R  N  D  T  S  R  P  P  L  P  L
agggtccctgggtgcccagctccctgtccacttgggcgcttccagcagctgactgctccagcccggcctccagcccatggggccccc
 R  V  P  G  C  P  A  P  C  P  L  G  R  F  Q  Q  L  T  A  P  A  R  P  P  A  H  G  A  P
tgccatggttcttatgagcctgccagcccccccagccacggtgcccctgctggccggagctgtggctgtgctggccgtgctaagcctg
 C  H  G  S  Y  E  P  A  S  P  P  A  T  V  P  L  L  A  G  A  V  A  V  L  A  V  L  S  L
gggcttggtcttctggcctggaggcccagatgcctgcgggccctgggagggactgtgtga
 G  L  G  L  L  A  W  R  P  R  C  L  R  A  L  G  G  T  V  *
```

Figure S4. Exon Composition of Membrane-Bound Form of Mouse *Acp4*. This is the mouse *Acp4* NCBI Gene Reference Sequence showing the cDNA sequence and deduced 425-amino-acid sequence. At the top is the range of each exon in the genomic sequence highlighted with the same colors shown in the cDNA sequence. This protein is equivalent to the only form of ACP4 found in humans. According to the exon designations shown in Fig. S2 and Fig. S3, the structure of this mouse cDNA is: Exon 1c (yellow), then exons 3 through 12. This is the deduced protein sequence shown in the alignment in Fig. S1 (m*Acp4*). Note that the transmembrane and cytoplasmic domains of ACP4 are encoded by exon 12, the last coding exon.

4144..4251, 4330..4434, 4512..4598, 5353..5499, 6079..6177, 6257..6352, 7014..7140, 7235..7317, 7677..7804, 7892..8070, 8906..9338.

```
atggctgagcccgggtctcagggccacaccgtcggacccttgctgctgctgctgttgctgttgctgcctcgggccctgccggagggg
 M  A  E  P  G  S  Q  G  H  T  V  G  P  L  L  L  L  L  L  L  L  P  R  A  L  P  E  G
cccctgttatttgtggctctggtgttccgacatggcgaccggccccactggcctcctaccccacagatccacacaaggaagctgcc
 P  L  L  F  V  A  L  V  F  R  H  G  D  R  A  P  L  A  S  Y  P  T  D  P  H  K  E  A  A
tccaccttgtggcctcgaggcttgggccaactgactaaggaggggatccgccagcagctagaactgggccgatttctgaggaggcgt
 S  T  L  W  P  R  G  L  G  Q  L  T  K  E  G  I  R  Q  Q  L  E  L  G  R  F  L  R  R  R
tacaaggctttcctgagccctgagtacaagcgagaagaggtgtacatccgcagcacagactttgaccggacattggagagtgcacaa
 Y  K  A  F  L  S  P  E  Y  K  R  E  E  V  Y  I  R  S  T  D  F  D  R  T  L  E  S  A  Q
gccaacctggctgggctcttccctgaggctgcccctggaagtcctgagactgactggaagcccattccagtgcacacagtgcctgtg
 A  N  L  A  G  L  F  P  E  A  A  P  G  S  P  E  T  D  W  K  P  I  P  V  H  T  V  P  V
tctgaggacaagttgctgaggttccccatgcgcagctgtcctcgataccatgagctgttacgagagtccacagaggcagctgactac
 S  E  D  K  L  L  R  F  P  M  R  S  C  P  R  Y  H  E  L  L  R  E  S  T  E  A  A  D  Y
caggaggccctggagggctggacggacttcctgacccgcctgggcaacttcactgggctgtccctggttggagagccactccggaga
 Q  E  A  L  E  G  W  T  D  F  L  T  R  L  G  N  F  T  G  L  S  L  V  G  E  P  L  R  R
gcatggaaagttctggataccttgatctgccagcgtgcccatggtcttgaccttccatcctgggcctctccagatgtcttgaggact
 A  W  K  V  L  D  T  L  I  C  Q  R  A  H  G  L  D  L  P  S  W  A  S  P  D  V  L  R  T
ctgtcacagatttctgctctggatatcagggcccatgtaggcccaccccgagcagcagaaaaggcccagctgacaggggggatcctg
 L  S  Q  I  S  A  L  D  I  R  A  H  V  G  P  P  R  A  A  E  K  A  Q  L  T  G  G  I  L
ctggatgctattctcagcaatttctcccggacccagcgccttgggttgcccctcaagatggtcatgtactcagctcatgacagcacc
 L  D  A  I  L  S  N  F  S  R  T  Q  R  L  G  L  P  L  K  M  V  M  Y  S  A  H  D  S  T
ctgctggccctccaggggccctgggcctctacgatgggaacaccccaccttacgctgcctgcatggcctttgaattccggggggagc
 L  L  A  L  Q  G  A  L  G  L  Y  D  G  N  T  P  P  Y  A  A  C  M  A  F  E  F  R  G  S
tccagggaacccgaggaggaagatggagagaatgtcactgtctctctcatctaccgcaatgacacctcccgcccacccctgccactc
 S  R  E  P  E  E  E  D  G  E  N  V  T  V  S  L  I  Y  R  N  D  T  S  R  P  P  L  P  L
agggtccctgggtgcccagctccctgtccacttgggcgcttccagcagctgactgctccagcccggcctccagcccatggggccccc
 R  V  P  G  C  P  A  P  C  P  L  G  R  F  Q  Q  L  T  A  P  A  R  P  P  A  H  G  A  P
tgccatggttcttatgagcctgccagcccccagaccgctggagtatcctctggaaacaggccaaaggtgggcaagtgcgcctgtgc
 C  H  G  S  Y  E  P  A  S  P  P  D  R  W  S  I  L  W  K  Q  A  K  G  G  Q  V  R  L  C
ctcacctgctctggacccagctcatcacccagattctacccacgccggaaccgaatcaaacagcacaccctacagagggcaatgaac
 L  T  C  S  G  P  S  S  S  P  R  F  Y  P  R  R  N  R  I  K  Q  H  T  L  Q  R  A  M  N
cagccatttaatagaaactcacgaaggcagaagttccagggctgcccacctcacccctccactaaatccagatctgcattggccagg
 Q  P  F  N  R  N  S  R  R  Q  K  F  Q  G  C  P  P  H  P  S  T  K  S  R  S  A  L  A  R
ggccatctgtgcagccagggagaccagacccggctggaaggtgcttcaaaagggaagggcatggttaaaagaaaacggggacaaggt
 G  H  L  C  S  Q  G  D  Q  T  R  L  E  G  A  S  K  G  K  G  M  V  K  R  K  R  G  Q  G
ccccaaagcaaaatctttacaaaaggggggttctcctctccccaaaaccaaagtgtcacataagctcaaaataaaatataaagtaaaa
 P  Q  S  K  I  F  T  K  G  G  S  P  L  P  K  T  K  V  S  H  K  L  K  I  K  Y  K  V  K
atgctgcattagaaaagtttgcaatgttaagc
 M  L  H  *
```

Figure S5. Exon Composition of Potentially Secreted Form of Mouse ACP4. This is the cDNA sequence of the other mouse *Acp4* transcript detected in EOE and its deduced 525-amino-acid sequence. This cDNA formed by alternative splicing. Exon 12 was skipped during splicing and a downstream exon 13 included. At the top is the range of each exon in the genomic sequence highlighted with the same colors highlighting the cDNA sequence. This protein is unlike the form of ACP4 expressed in humans due to its novel 3' cDNA sequence that replaces the transmembrane and cytoplasmic domains; thus, the alternative form of ACP4 is likely to be secreted. According to the exon designations shown in Figs. S2 and S3, the structure of this mouse cDNA is: Exon 1c (yellow), then exons 3 through 11, then exon 13. This cDNA sequence is not listed in NCBI (although there are other alternatively spliced *Acp4* cDNA sequences in NCBI that contain exon 13). It was discovered by amplifying cDNA obtained from the enamel organ epithelia of mouse molars.

aggccacagcacagacggaggctgagctcagcgtctgctctctgtggaa**atg**gctgagcccgggtctcagggccacaccgtcggacccttgctg
ctgctgctgttgctgttgctgcctcgggccctgccggagggcccctgttatttgtggctctggtgcgaagccctccgaccccacctgacccac
cctctaccttccaagtcagtcctgacctggttctccaacatccccaggtgttccgacatggcgaccgggccccactggcctcctaccccacaga
tccacacaaggaagctgcctccaccttgtggcctcgaggcttgggccaactgactaaggtgaggagggagatggggtgagaaagagggctaggg
gtcaggcgatgagggtctgctgaggcccctctgtccctaggaggggatccgccagcagctagaactgggccgatttctgaggaggcgttacaa
ggctttcctgagccctgagtacaagcgagaagaggtactttgttgacctttgaccccagagcccccttggtcttcacctcttgtctttgatct
ccatctatttgacctgcaccccctatgggcttctaagtcagtagggaatccacaccgacctctgaccttctctcaaacgatctgtctttccactc
actggacctcaacctttgccttgatttccaaccgccccatcctgacctcctgacctttgaacccaactctgtctttgatgcttgctttgcccac
ctgatgtctgttaggggtggggtttcttggtaaggatctggacctgtgcatgcttggcaagccctccaccactgggtaccaccccccatttcca
acctccaacctttgctccctaccatcagcattttgaccttcctttctgtcttctcacttccgttggctggcccctgacttctctgaggactgt
gtaatcaaatttagctccatggtattcagttcccaccttctacatcttgacctcttactcccaggccccttgatctgatctcagcagaccccag
cagtccaacacatcactcaactctgacctttgatcccaacttccaaagctgaaagttagacacatgaattcaccttgagttttgaccctgaatg
cctgtcctttgaccccaccctcttcctgacctttaaccttacgcactccaaactaaatctgaagtttttgatttgagaccctgctgacccttgg
cctgtccacctgctctgctgccctcatccaccccaggtgtacatccgcagcacagactttgaccggacattggagagtgcacaagccaacctgg
ctgggctcttccctgaggctgcccctggaagtcctgagactgactggaagcccattccagtgcacacagtgcctgtgtctgaggacaaggtcag
gggcttcatctggtgggagaaacagaagaatcgagtttaatctcattctgggaaccttaatgagactttctcaaaatgaaaaaaaaaaaaaac
aaacaaacagtttaacaagaggagtggaatgtagtataggggtaaacccccccagcttagaatcctctagtgaggagggctgggaggagtgtgg
ctcagtggtaaactacctacctagggaggagctgggggtgtggcttagaggtagaaggatttcctagcctgtccaaggttctgggttctataa
ccaacaccacacatacaaaaaaggatggaaataaagcccagtactgagctggagaggggaagaggcagctccaagccaggtggatccatttctg
gaagcccagaacagctgagatcagaaaggttatccctggtagagctagctgtccagagttggtaagctgacacccctgcccagcacagctggat
aacaggggaaactgggagagttgagggctagagaccctggccccatggccaactgtgagaccccagacttgctgctgccttccctgagcctcag
tttcccccagttgctgaggttccccatgcgcagctgtcctcgataccatgagctgttacgagagtccacagaggcagctgactaccaggaggcc
ctggagggctggacggtgagtgctggtgtggctggtgtggctgggtgccaagcaacaactggtaggctcacccagccctgtatctccactcag
gacttcctgacccgcctgggcaacttcactgggctgtccctggttggagagccactccggagagcatggaaagttctggataccttgatctgcc
aggtgagtccttttctcccctctcccatctggggatttcaaatatgcaaatatcagggcaagagatttagctctgcgtaccctggtttccccat
ctgcgtggtggggatagtggcaggctgatgagatggtggcatagcatatatatatatatataaaatctcctttcctggtaccatttcattggat
ctttctagaaaaccgaatgtcagattccaaggcatctctggtcccacagggcttggtaaggactctggtctggtacttggtctggctcatcctg
tgctgaactgatctttgaggggtttgaatgagggccagggtctggagggttggctgtctgtaaggtgcttgccacacaaacatgaggaatttag
atcactagtacctacatgaaaaagctgggtgcagcagctcacacggaaatcctgctgatgaggctttgacaggaaaatcctggggttcagtagc
cagccaacttggtctacttgttgagttccaggccaggggaggctctcaaacaaacgaggtgggccaggcttgaggaacatatgcaggtaacctc
taacctaggtgcacacacacacacacacacactccaaaacaaatgggggggggttgactgaggactcccacttgagaccctttctctctccact
ctcagcgtgcccatggtcttgaccttccatcctgggcctctccagatgtcttgaggactctgtcacagatttctgctctggatatcagggccca
tgtaggcccaccccgagcagcagaaaaggcccagctgacagggggtaaggtgtggggtctgtgggcttgggggagctggtttgcaggtggagag
ggggcatctacatactctcttccccgtctgtccctcagggatcctgctggatgctattctcagcaatttctcccggacccagcgccttgggttg
cccctcaagatggtcatgtactcagctgtgagtccttgggaagggcaggcactgctgcatgagtgacaggggttgtggtggtggtggtgagact
acagtgcctatgcaatctcagtctccagccttggctcaccctcatctcctacagtacagtactgcagcattagaaacacagtaaagatgaggga
gggggtcagagaagcaagactttagtcctgaaaggactgactgtagaatgggaactgcccagtcacaatttgcacgtttggacaatcattatag
aatgcctatgatgtgaatgggagtggtctgggattacttctaactggagacaatctaggtgctggggttctgatttagcttccatccctggaaa
ttctcttcagcatgacagcaccctgctggccctccagggggccctggcctctacgatgggaacaccccaccttacgctgcctgcatggccttt
gaattccgggggagctccagggaacccgaggaggaagatggagagtgaggatgggtgatggcttggcattgggtggatgggctcttcttcggat
ggggaactagtgatgctactgtcctttccacctccaggaatgtcactgtctctctcatctaccgcaatgacacctcccgcccaccccctgccact
cagggtccctgggtgcccagctccctgtccacttgggcgcttccagcagctgactgctccagcccggcctccagcccatgggcccccctgccat
ggttcttatgagcctgccagcccccagrgtgacagccctctacgctggggtgggagtggggagttcccaaggcctgggattcacaacccccatg
ttcttcctgcagccacggtgcccctgctggccggagctgtggctgtgctggccgtgctaagcctggggcttggtcttctggcctggaggcccag
atgcctgcgggccctgggagggactgtg**tga**gggggggatgaatagacacaaaaataccccgagctgacactggatcccaacacgtgtgctcact
tgctgctctggcttgtcacttactgtgctcatgcgcgtgtggggatgaaggctgggtgggcctcaagcattatgcttgcttggacactgcaatc
aggtgttgtacatacaggtgtcggggctcacaataagcacgtgtgcaggtgggaacctgtagcctgagagcacacaggactcaattgccttaag
caactctaaccctggtatggtggcagaacagatagagatagagatccctccacttgagggagcctgccagcccccccaggtgacacttgagggag
ggcttaactcctactaaggaacagcctaccagcagccactgtgtcactgccatcacctggagccccatgcccattgccttgtgttcatcattcc
ccagagccccccagccctgtcttgagaggtttcctatctgggggaggtacatcaagacctaagagcctaaggaagctaaggagcataccctgga
atccaagaacagcttgaactagagggtctgatcaattgcaggctctgaggtaggggataaggccagccagtggctgtgactggcccccaccagc
ccacctttcctcaccagaccgctggagtatcctctggaaacaggccaaaggtgggcaagtgcgcctgtgcctcacctgctctggacccagctca
tcacccagattctacccacgccggaaccgaatcaaacagcacaccctacagagggcaatgaaccagccatttaatagaaactcacgaaggcaga
agttccagggctgcccacctcacccctccactaaatccagatctgcattggccaggggccatctgtgcagccagggagaccagacccggctgga
aggtgcttcaaaagggaagggcatggttaaaagaaaacggggacaaggtccccaaagcaaaatctttacaaaggggggttctcctctccccaaa
accaaagtgtcacataagctcaaaataaaatataaagtaaaaatgctgcat**tag**aaaagtttgcaatgttaagc

Figure S6. Partial Intron/Exon Structure of Mouse *Acp4* Gene. This gene segment contributes to the two alternatively spliced *Acp4* transcripts detected in developing mouse molars. The sequence shown includes nucleotides 4125-9338 of the mouse NCBI *Acp4* Reference Sequence: NC_000073.7. Both *Acp4* transcripts expressed the same coding regions from Exons 1c, and Exons 3 through 11: 4144..4251, 4330..4434, 4512..4598, 5353..5499, 6079..6177, 6257..6352, 7014..7146, 7235..7317, 7677..7804, 7892..8070, but differed by the inclusion or skipping of exon 12 during mRNA splicing. When exon 12 (8149..8261) is included, the ACP4 translation terminates in exon 12 generating a 425-amino-acid protein that includes a C-terminal transmembrane domain and short cytoplasmic domain. When exon 13 (8906..9338) is included, instead of exon 12, the resulting 525 aa protein lacks the transmembrane and cytoplasmic domains and may be secreted.

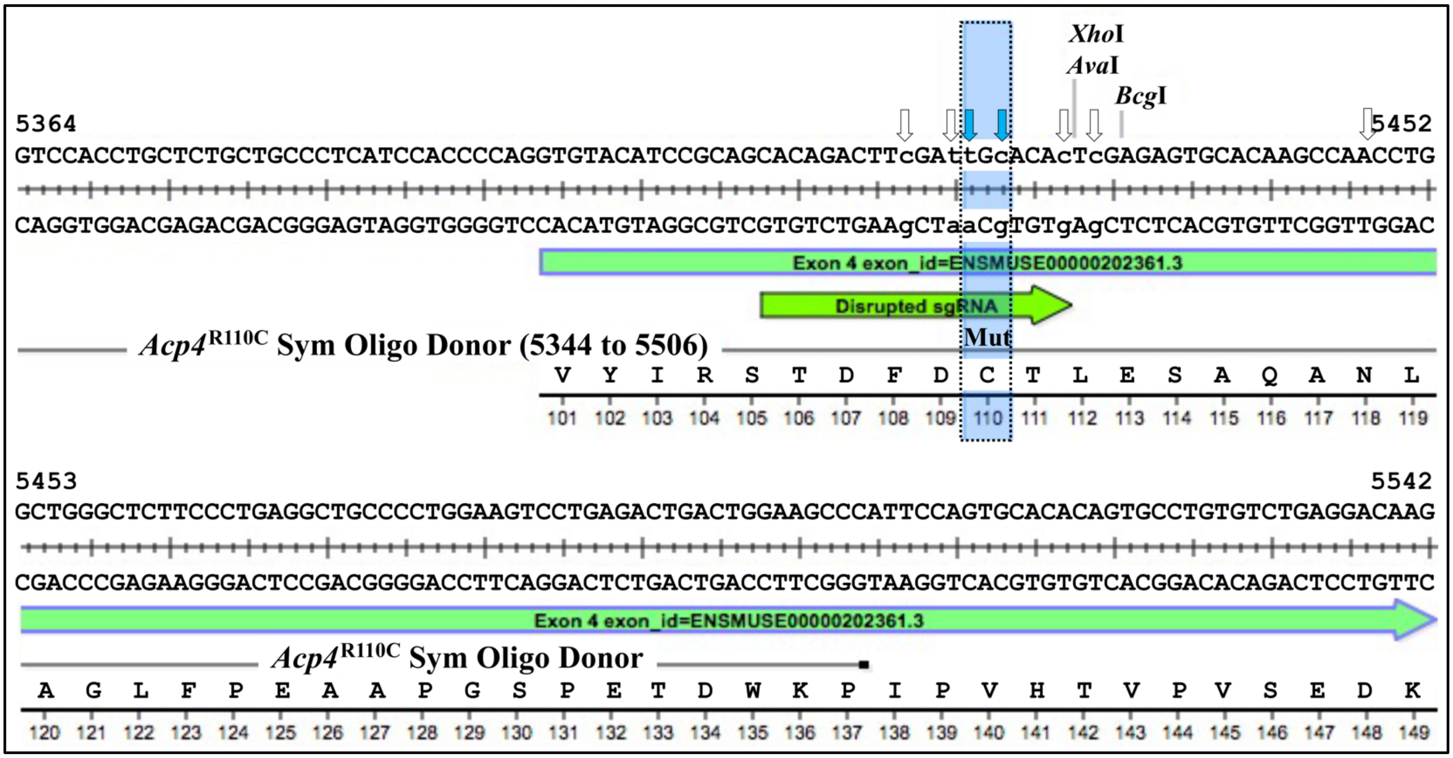

Figure S7. CRISPR/Cas9 Strategy to Generate *Acp4*^{R110C} Knockin Mice. The mouse C57BL/6J chromosome 7 region (43901427...43910814) containing the *Acp4* gene reference sequence (NC_000073.7) was analyzed to generate an *Acp4*^{R110C} mouse using CRISPR/Cas9 gene editing. The 5' region of *Acp4* Exon 4 (green bar) was analyzed to design the single guide RNA (sgRNA; green arrow) to direct a Cas9 nuclease cleavage within the Arg110 codon. The symmetrical oligonucleotide donor (Sym Oligo Donor, NC_000073.7 nucleotides 5344 to 5506) provided a repair template guide to facilitate homology-directed repair while introducing four silent variations (white arrows) and two variations that convert codon 110 into a TGC cysteine codon (blue arrows and blue box). These sequence variations potentially facilitated PCR genotyping by introducing genotype-specific oligonucleotide priming sites and an *Xho*I (C'TCGAG) and *Ava*I (C'YCGRG) restriction sites.

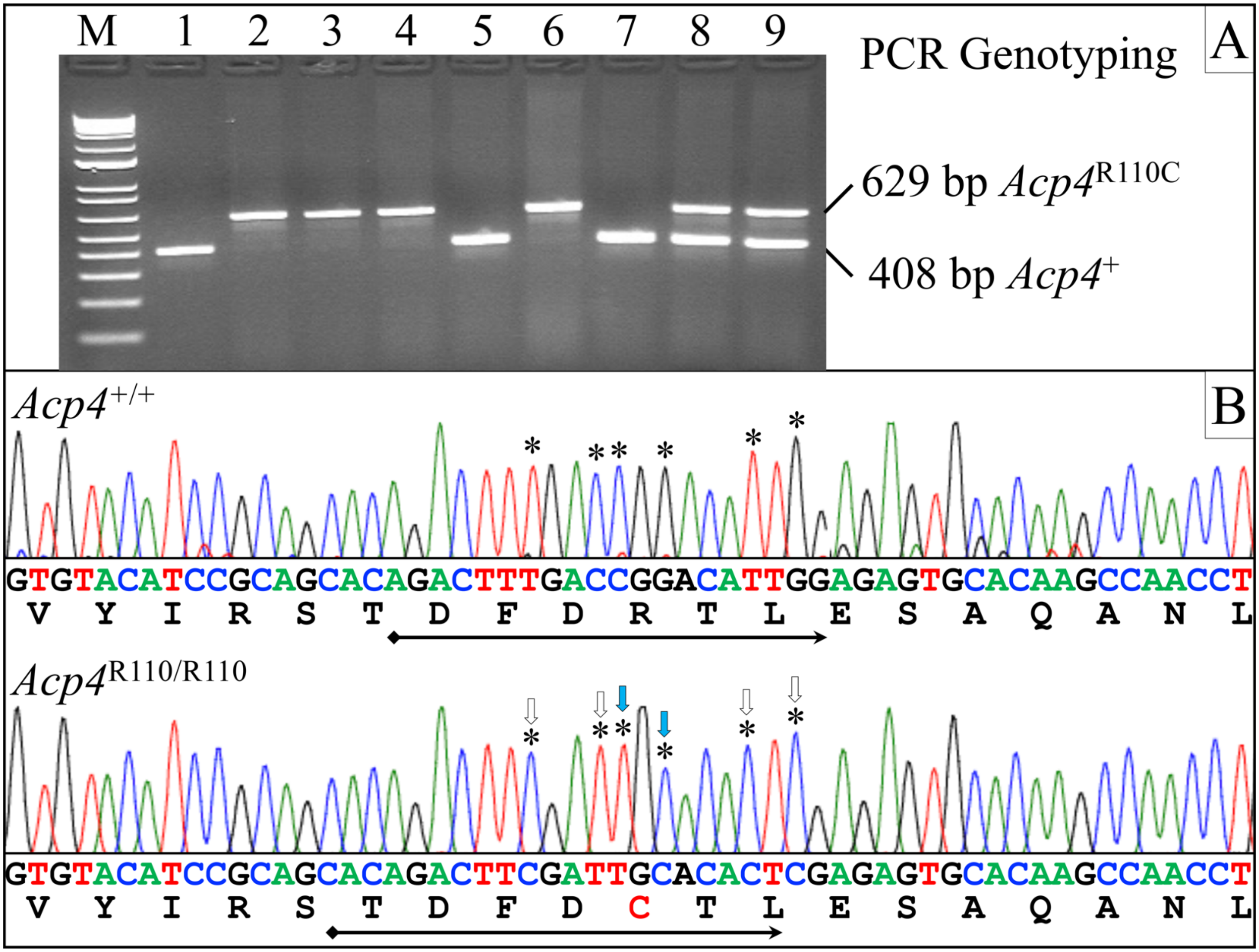

Figure S8. *Acp4*^{R110C} Genotyping and Validation. ***A:*** Mouse genomic DNA from tail biopsies was separately amplified with an *Acp4*^{R110C}-specific primer pair (F: CACAGACTTcGAttGcACAcT; R: ACTCTCCCAGTTTCCCCTGT) and an *Acp4*$^{+}$-specific primer pair (F: AGACTTTGACCGGACATTGG; R: CCAGAACCTTGGACAGGCTA). The amplification products varied in length due to variations in the annealing positions of the 3' primers. The two amplification reactions for each mouse were combined, incubated at 37 °C for 30 min, and separated on a 1.5% agarose gel stained with ethidium bromide. The *Acp4*^{R110C} amplification product was 629 bp; the *Acp4*$^{+}$ amplification product was 408 bp. The *Acp4*$^{+/R110C}$ (heterozygous) mice displayed both products. ***B:*** Genomic DNAs from *Acp4*$^{R110C/R110C}$ and *Acp4*$^{+/+}$ mice were amplified and characterized by DNA sequencing and showed no sequence variations in the coding region or adjacent intron borders excepting the six sequence variations intentionally introduced in exon 4. The 5’ annealing sites for the *Acp4*^{R110C}-specific and *Acp4*$^{+}$-specific primer pairs are indicated by arrows. The PCR conditions were 94 °C for 2 min, then 35 cycles of [94 °C for 30 s, 59 °C for 30 s] followed by 72 °C for 60 c, 72 °C for 60 sec and then hold at 4 °C. Each PCR reaction contained 10 µL of Platinum Hot Start PCR Master Mix (2x) (Invitrogen, Carlsbad, CA, USA), 1 µL of 10 µM Primer mix, 3 µL of DNA (40-50 ng/µL) template and 6 µL distilled water. The reactions were run using a GeneAmp PCR System 9700 (Applied Biosystems, Foster City, CA, USA).

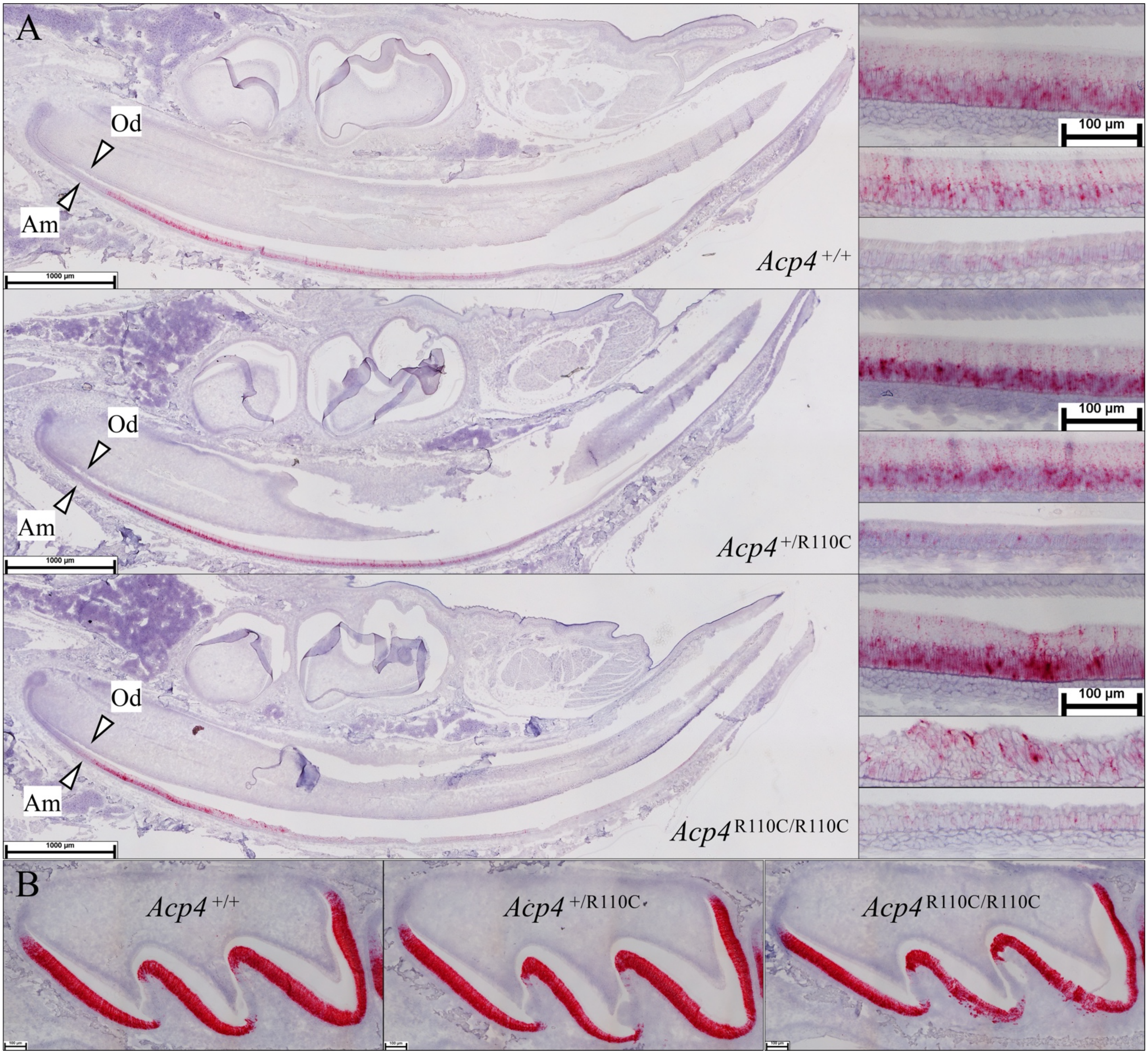

Figure S9. *In Situ* Hybridization of *Acp4* in *Acp4*$^{+/+}$, *Acp4*$^{+/R110C}$, and *Acp4*$^{R110C/R110C}$ Mouse Teeth. ***A:*** Mandibular incisors of D12 mice. The continuously growing mouse incisors show ameloblasts in a progressively later stage of development going from left (secretory stage) to right (maturation stage). On the right are 3 higher magnification views of ameloblasts: early secretory stage (top), late secretory stage (middle), and maturation stage (bottom). *Acp4* mRNA signal is strongest during the secretory stage and gradually diminishes near the end of the secretory stage and is still detectable during the maturation stage. About midway through the secretory stage the ameloblasts of *Acp4*$^{R110C/R110C}$ incisor undergo pathologic changes after which there is only spotty *Acp4* expression. ***B:*** Maxillary first molars of D4 mice. At this point all ameloblasts are in the secretory stage and show a strong *Acp4* mRNA signal, except for the ones at enamel-free areas. Am, ameloblast; Od, odontoblast.

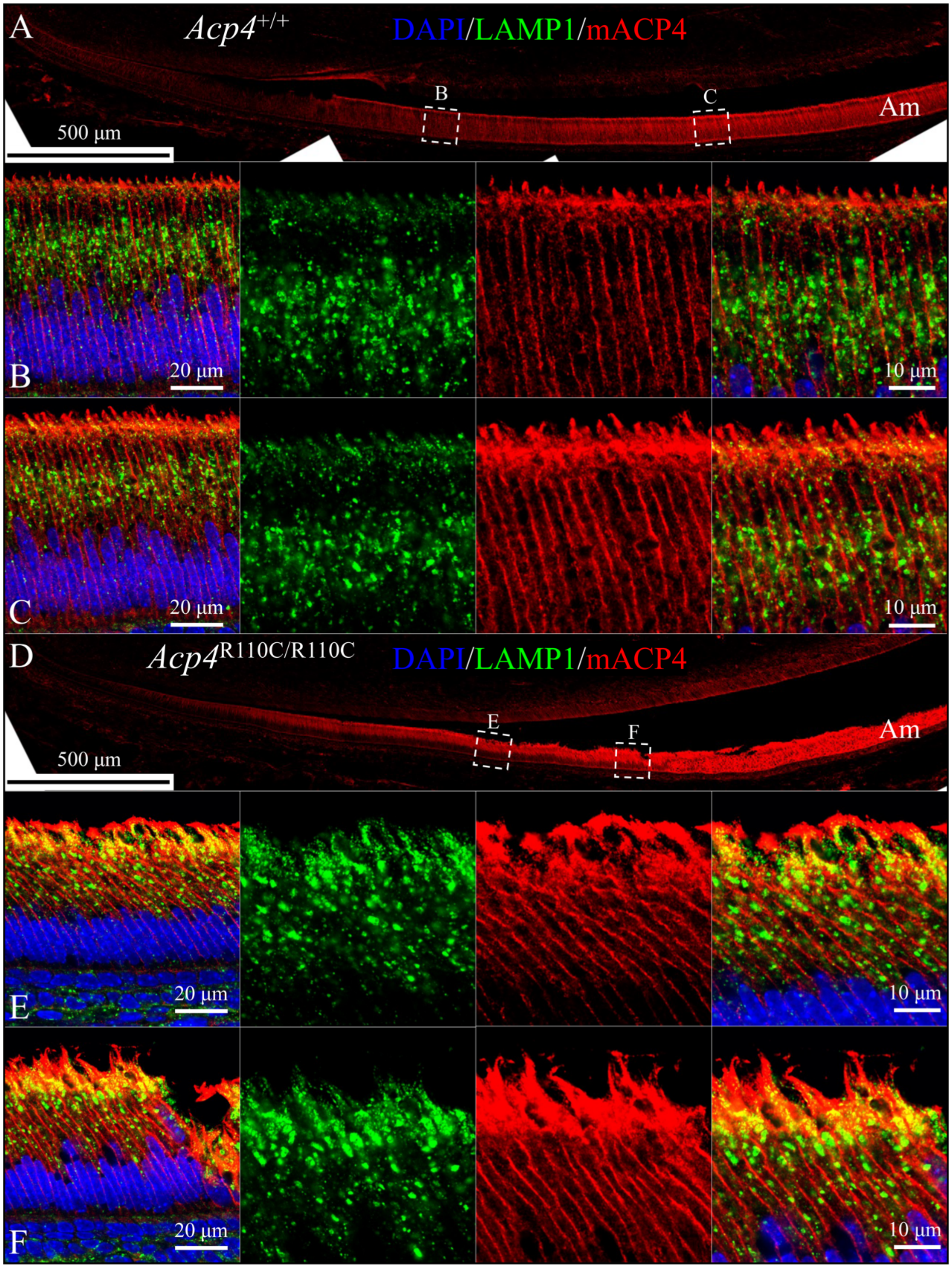

Figure S10: Immunohistochemistry of ACP4 (anti-mouse ACP4 antibody) and LAMP1 in *Acp4*$^{+/+}$ (WT) and *Acp4*$^{R110C/R110C}$ D12 Mandibular Incisors. LAMP1 (late endosomes and lysosomes) is labeled in green. ACP4 (custom anti-mouse ACP4 antibody) is labeled in red. Nuclei are in blue. Apical region of *Acp4*$^{+/+}$ (WT) (**A**) and *Acp4*$^{R110C/R110C}$ **(D)** mandibular incisors highlighting ameloblasts (Am) and odontoblasts (Od) and boxing areas imaged below at higher magnification. ACP4 signal is specific for secretory ameloblasts and concentrates in their Tomes' processes. Higher magnification images for WT (**B-C)** and *Acp4*$^{R110C/R110C}$ **(E-F)** from left to right show tri-staining of nuclei blue, LAMP1 green, and mACP4 red; succeeded by higher magnifications of the Tomes' process side of ameloblasts stained for LAMP1 only, mACP4 only, with all 3 stained on the right panel. The LAMP1 signal in the distal half of secretory ameloblasts groups into two clusters: one that includes the Tomes' process (TP) and distal terminal web (TW), the other the supranuclear (SN) region. The area between these two clusters is relatively sparse in LAMP1 signal. Most of the ACP4 signal is restricted to the Tomes' process and distal terminal web area surrounding the distal cluster of LAMP1 signal. Some ACP4 signal is observed in the cytoplasm and along the lateral plasma membrane.

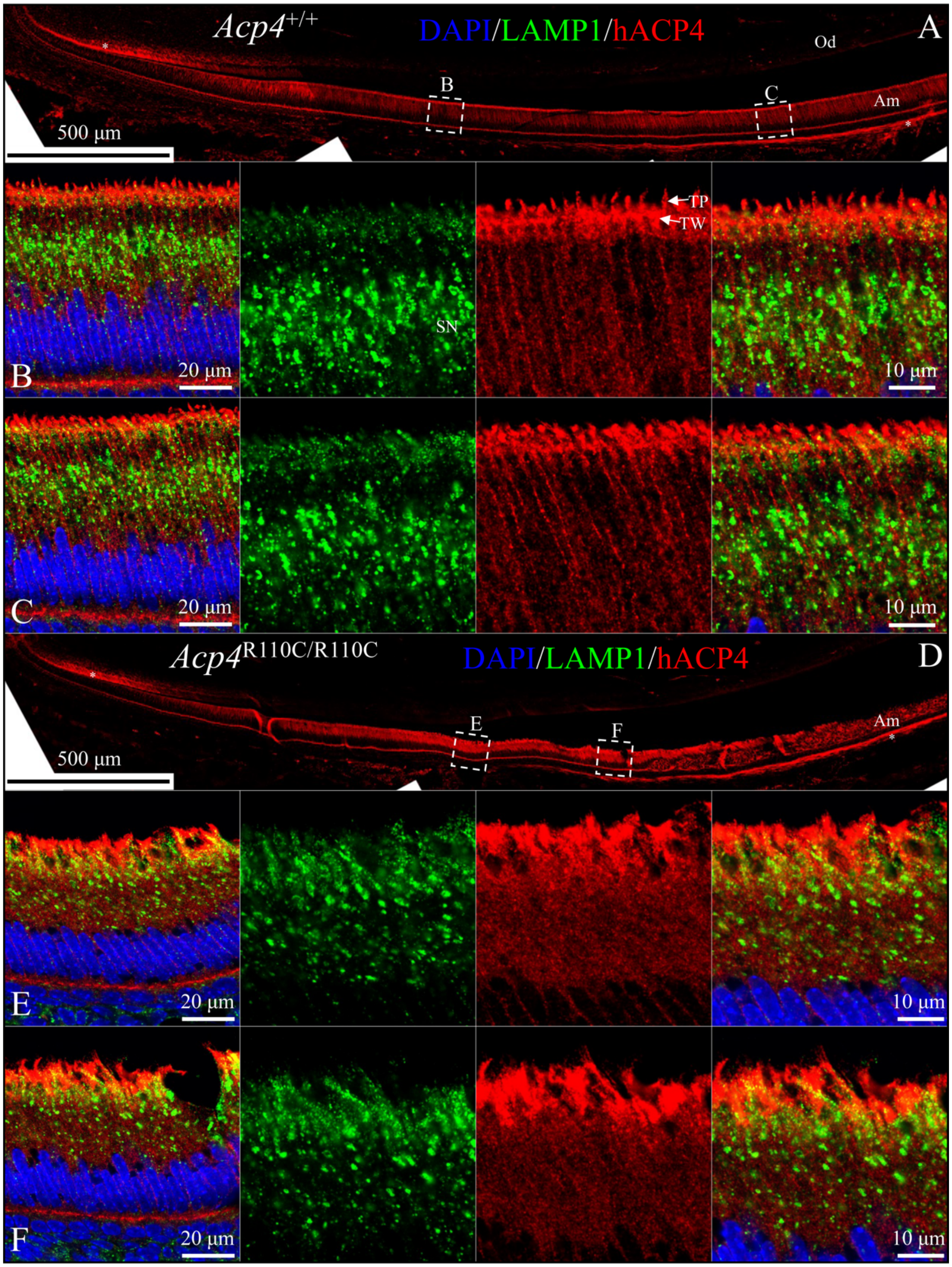

Figure S11: Immunohistochemistry of ACP4 (anti-human ACP4 antibody) and LAMP1 in *Acp4*$^{+/+}$ (WT) and *Acp4*$^{R110C/R110C}$ D12 Mandibular Incisors. LAMP1 (late endosomes and lysosomes) is labeled in green. ACP4 (orb101887 antibody) is labeled in red. Nuclei are in blue. Apical region of *Acp4*$^{+/+}$ (WT) (**A**) and *Acp4*$^{R110C/R110C}$ **(D)** mandibular incisors highlighting ameloblasts (Am) and odontoblasts (Od) and boxing areas imaged below at higher magnification. ACP4 signal is specific for secretory ameloblasts and concentrates in their Tomes' processes. Higher magnification images for WT (**B-C)** and *Acp4*$^{R110C/R110C}$ **(E-F)** from left to right show tri-staining of nuclei blue, LAMP1 green, and mACP4 red; succeeded by higher magnifications of the Tomes' process side of ameloblasts stained for LAMP1 only, mACP4 only, with all 3 stained on the right panel. The LAMP1 signal in the distal half of secretory ameloblasts groups into two clusters: one that includes the Tomes' process (TP) and distal terminal web (TW), the other the supranuclear (SN) region. The area between these two clusters is relatively sparse in LAMP1 signal. Most of the ACP4 signal is restricted to the Tomes' process and distal terminal web area surrounding the distal cluster of LAMP1 signal. Some ACP4 signal is observed in the cytoplasm and along the lateral plasma membrane.

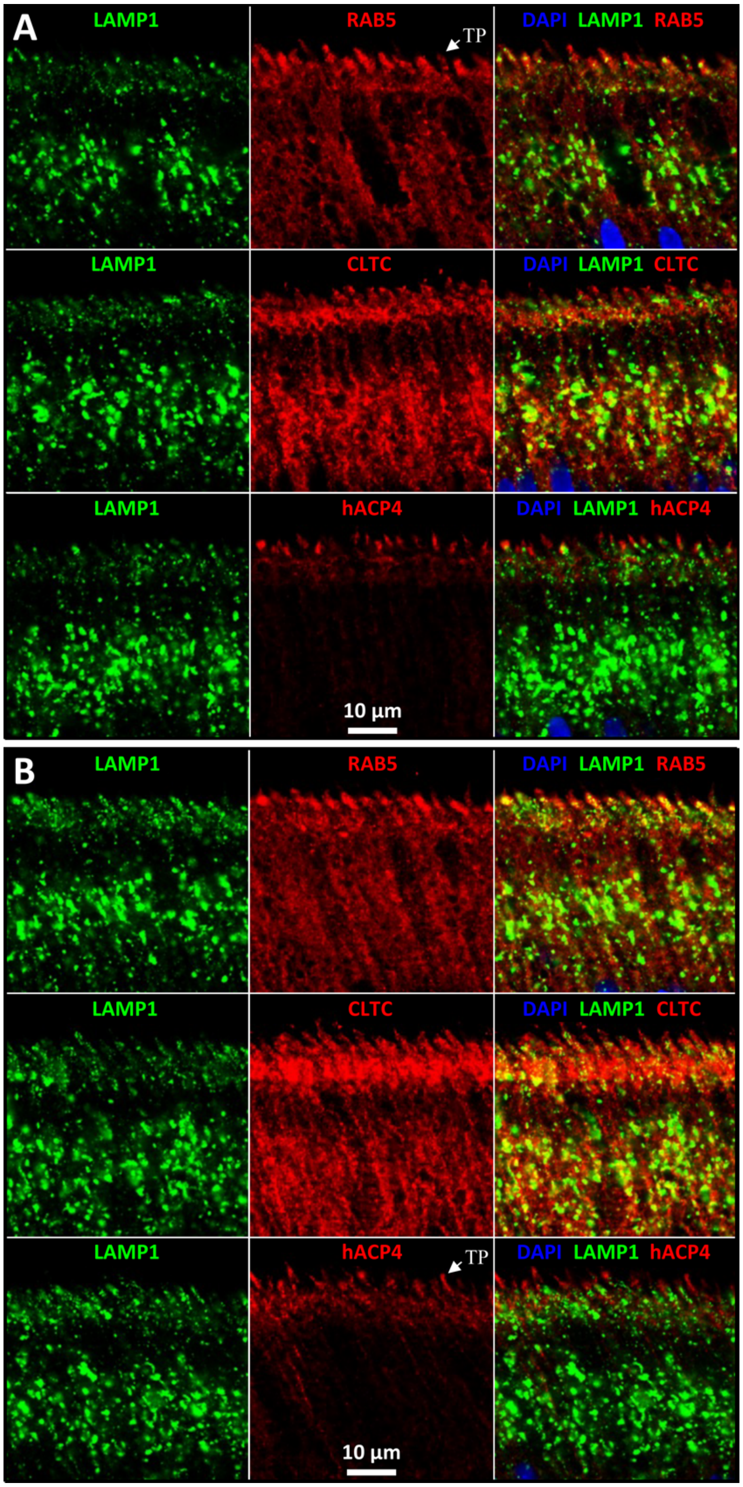

Figure S12. Immunohistochemistry of ACP4, LAMP1, RAB5, and CLTC on Ameloblasts of *Acp4*$^{+/+}$ D12 Mandibular Incisors. Longitudinally sectioned mandibular incisors were immunostained for ACP4 (orb101887 antibody) and endo-lysosomal markers, LAMP1 (lysosomes and late endosomes), RAB5 (early endosomes), and CLTC (clathrin-coated vesicles); DAPI was used to stain cell nuclei. Images were acquired from distal region of early secretory (***A***) and mid-secretory (***B***) stage ameloblasts. LAMP1-positive endo-lysosome organelles localized into two clusters, one with relatively smaller size at the Tomes' process (TP) and the distal terminal web, the other with relatively larger size closer to the nuclei. RAB5 representing early endosomes localized strongly, but less specifically in the Tomes' process. At the Tomes' process, RAB5 signal surrounded and overlapped with the LAMP1 signal. CLTC immunoreactivity also fell into two clusters, surrounding the two clusters of the LAMP1 signal. ACP4 localized almost exclusively at the Tomes' process, closely associated with the distal cluster of the LAMP1-positive organelles. However, the two signals appeared not largely overlapping.

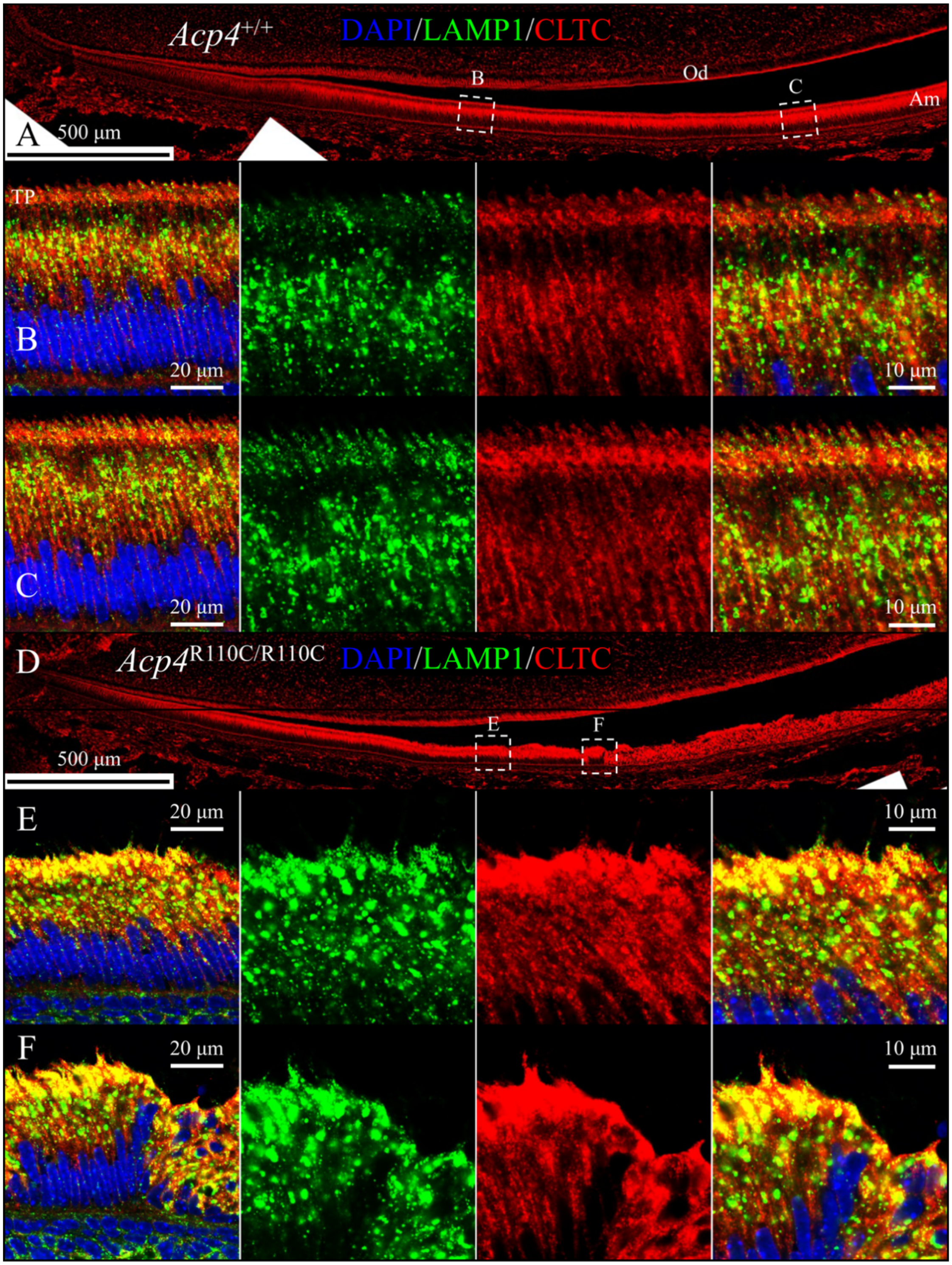

Figure S13. Immunohistochemistry of LAMP1 and CLTC in *Acp4*$^{+/+}$ and *Acp4*$^{R110C/R110C}$ D12 Mandibular Incisors. LAMP1 (late endosomes and lysosomes) is labeled in green, CLTC in red, and nuclei in blue. Longitudinal sections of the apical region of *Acp4*$^{+/+}$ (WT) (**A**) and *Acp4*$^{R110C/R110C}$ **(D)** mandibular incisors show boxed areas outlining the locations of ameloblast segments shown below at higher magnification. CLTC signal is detected in all cells, including ameloblasts (Am) and odontoblasts (Od). The higher magnification images for WT (**B-C)** and *Acp4*$^{R110C/R110C}$ **(E-F)** from left to right show tri-staining of nuclei blue, LAMP1 green, and CLTC red; succeeded by higher magnifications of the Tomes' process side of ameloblasts stained for LAMP1 only, CLTC only, and with LAMP1, CLTC and nuclei on the right panel. The CLTC immunoreaction exhibits two localization groups. One is at the Tomes' processes (TP) and distal terminal web (TW) area that colocalizes with LAMP1 signal. The other is at the supranuclear (SN) region where the Golgi apparatus localizes. While the signals of LAMP1 and CLTC are distinctly distributed, they are partly overlapping with each other. Am, ameloblast; Od, odontoblast; SN, supranuclear; TP, Tomes' process; TW, distal terminal web.

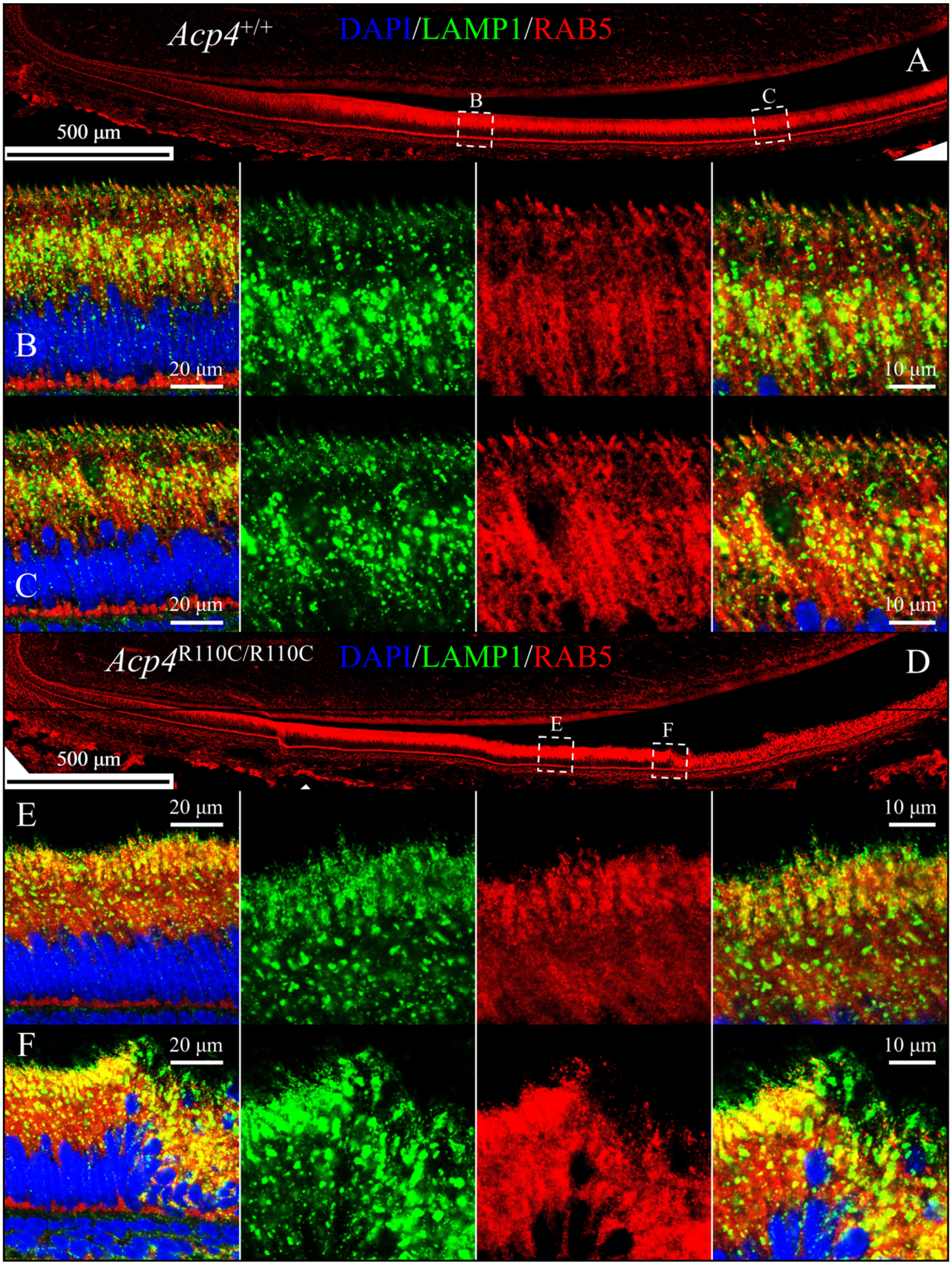

Figure S14. Immunohistochemistry of LAMP1 and RAB5 in *Acp4*$^{+/+}$ and *Acp4*$^{R110C/R110C}$ D12 Mandibular Incisors. LAMP1 (late endosomes and lysosomes) is labeled in green, RAB5 (early endosomes) in red, and nuclei in blue. ***A:*** Apical region of *Acp4*$^{+/+}$ incisor. RAB5 signal is the strongest in secretory ameloblasts. Boxed areas are shown with high-mag images. ***B, C:*** Boxed areas in A. Whole ameloblast cells are shown on the left, and their distal half on the right. Similar to LAMP1 and CLTC, the RAB5 signal also clusters at two areas, the Tomes' processes and the supranuclear region. ***D:*** Apical region of *Acp4*$^{R110C/R110C}$ incisor. Boxed areas are shown with high-mag images. ***E, F:*** Boxed areas in D showing the whole cell (left) and distal half (right) of the secretory stage ameloblasts. The distal cluster of RAB5 signal in *Acp4*$^{R110C/R110C}$ ameloblasts is stronger than that of *Acp4*$^{+/+}$.

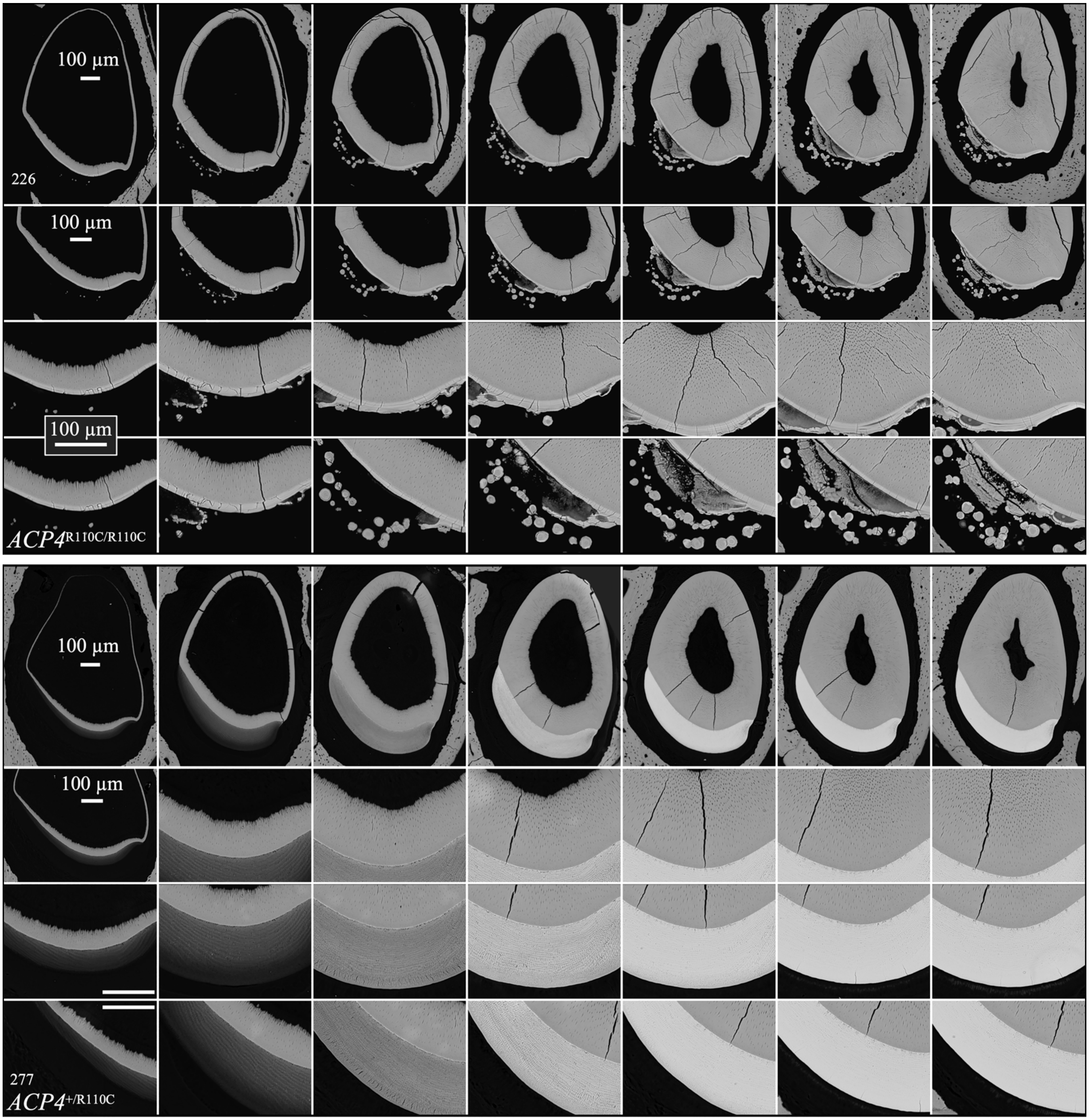

Figure S15. bSEM Images of *Acp4*$^{R110C/R110C}$ (226) and *Acp4*$^{+/R110C}$ (277) 7-week Mandibular Incisors Cross-sectioned at 1 mm Increments (levels 2 through 8). These are images from another 2 incisor samples, *Acp4*$^{R110C/R110C}$ (226) and *Acp4*$^{+/R110C}$ (277), analogous to those in Figs. 7, S16, and S17. The first two columns (Levels 2 and 3) show secretory stage enamel formation, where the enamel mineral ribbons elongate to their full thickness and the enamel layer achieves its final thickness, contour, and rod/interrod organization. Columns 3 through 7 (levels 4 through 8) show enamel maturation, where the thin crystals deposited during the secretory stage grow in width and thickness and the enamel layer as a whole achieves its final high degree of mineralization. *Acp4*$^{+/R110C}$ enamel appears to be totally normal. An enamel phenotype is only observed in the homozygous *Acp4*$^{R110C/R110C}$ mice.

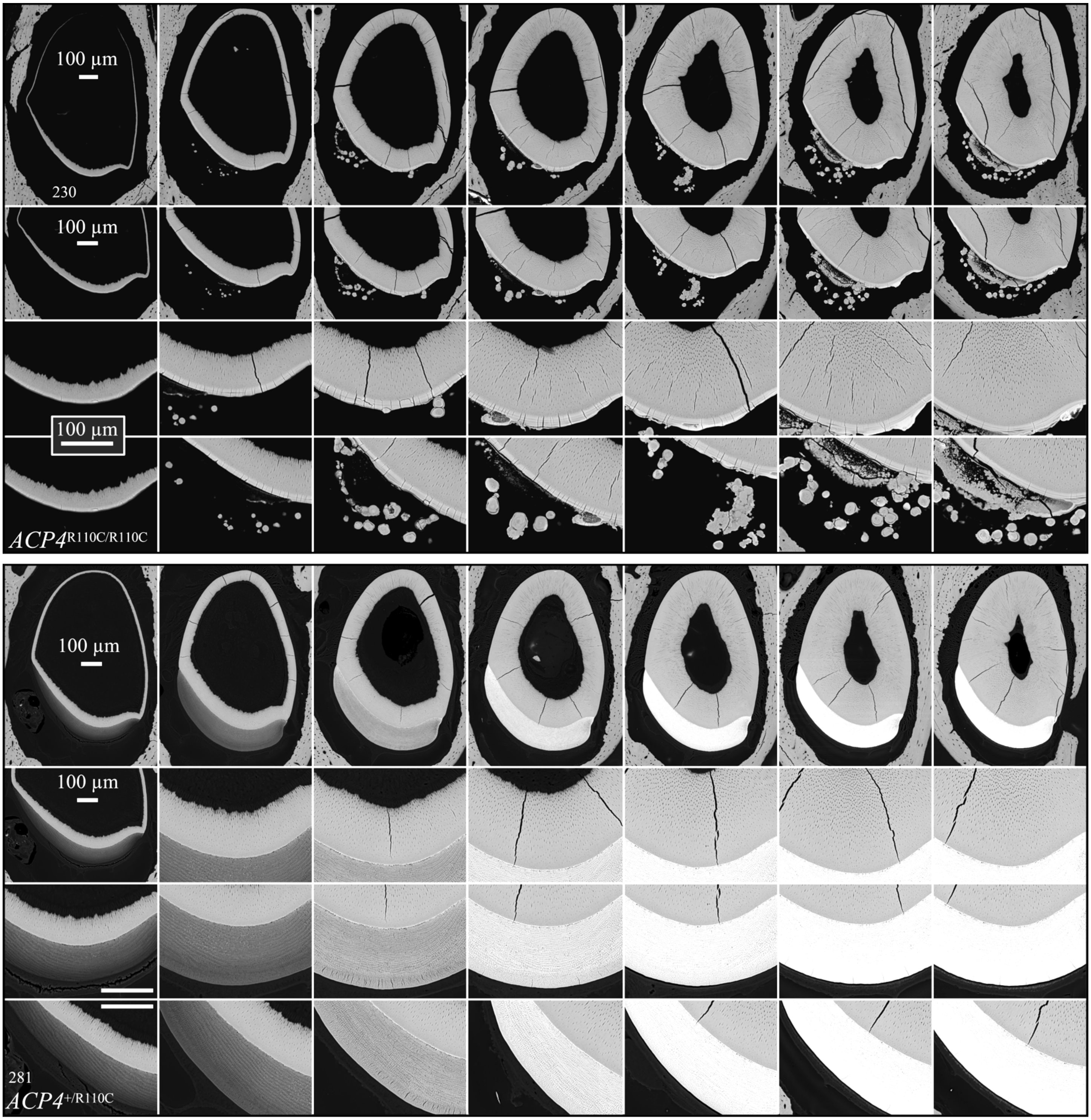

Figure S16. bSEM Images of *Acp4*$^{R110C/R110C}$ (230) and *Acp4*$^{+/R110C}$ (281) 7-week Mandibular Incisors Cross-sectioned at 1 mm Increments (levels 2 through 8). These are images from another 2 incisor samples, *Acp4*$^{R110C/R110C}$ (230) and *Acp4*$^{+/R110C}$ (281), analogous to those in Figs. 7, S15, and S17. The first two columns (Levels 2 and 3) show secretory stage enamel formation. Columns 3 through 7 (levels 4 through 8) show enamel maturation. The *Acp4*$^{R110C/R110C}$ incisors consistently deposit a thin layer of aplastic enamel on the surface of dentin, after which cell pathology has advanced to the point where mineral is ectopically deposited within the cellular layer and hardens into nodules. In contrast, *Acp4*$^{+/R110C}$ enamel appears to be totally normal.

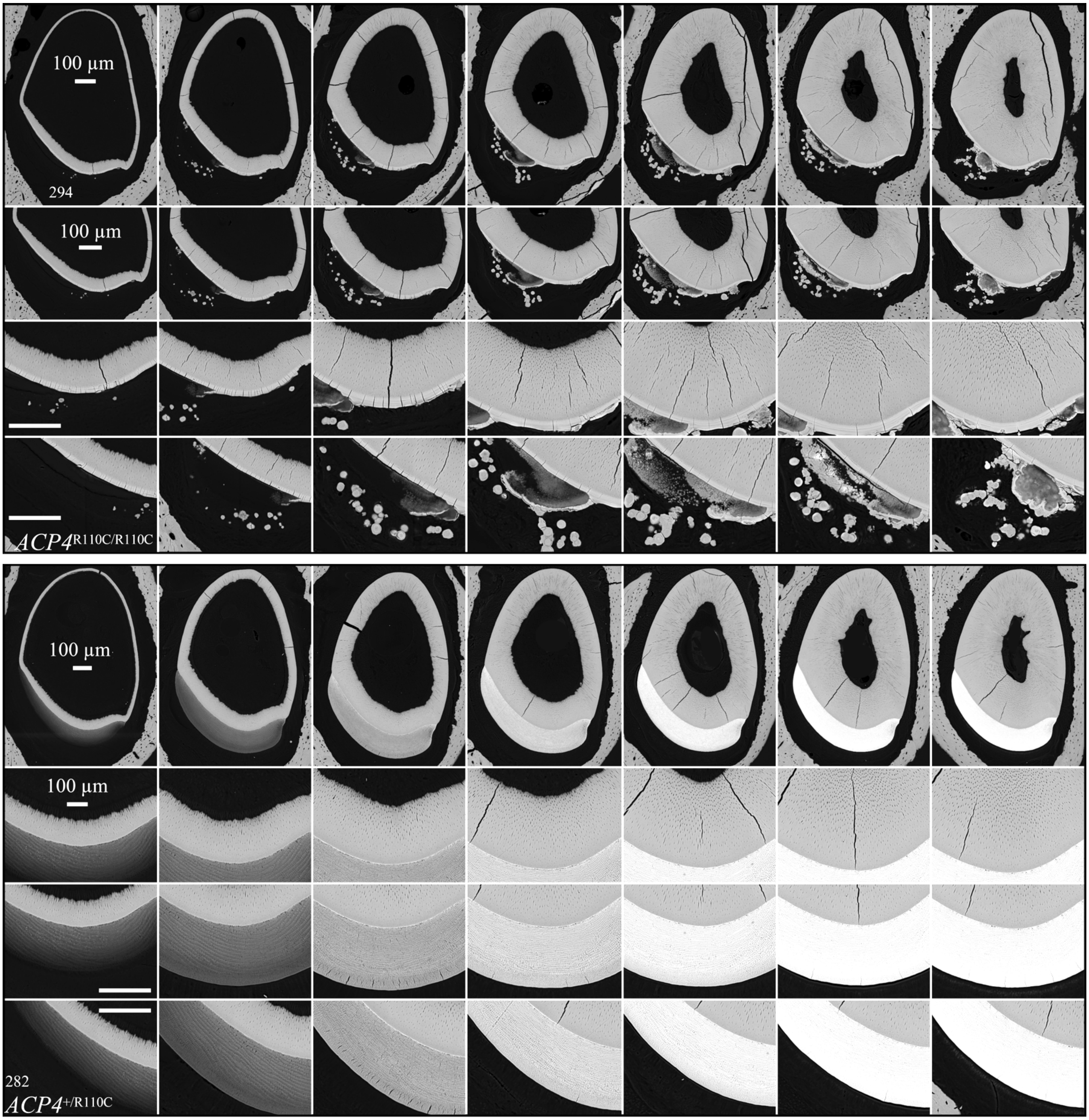

Figure S17. bSEM Images of *Acp4*$^{R110C/R110C}$ (294) and *Acp4*$^{+/R110C}$ (282) 7-week Mandibular Incisors Cross-sectioned at 1 mm Increments (levels 2 through 8). These are images show another 2 incisor samples, *Acp4*$^{R110C/R110C}$ (294) and *Acp4*$^{+/R110C}$ (282), analogous to those in Figs. 7, S15, and S16. The enamel phenotype is similar in all samples and only observed in the homozygous mutant mice. The first two columns (Levels 2 and 3) show secretory stage enamel formation. Columns 3 through 7 (levels 4 through 8) show enamel maturation. The *Acp4*$^{R110C/R110C}$ incisors consistently deposit a thin layer of aplastic enamel on the surface of dentin, after which cell pathology has advanced to the point where mineral is ectopically deposited within the cellular layer and hardens into nodules. *Acp4*$^{+/R110C}$ enamel appears to be totally normal.

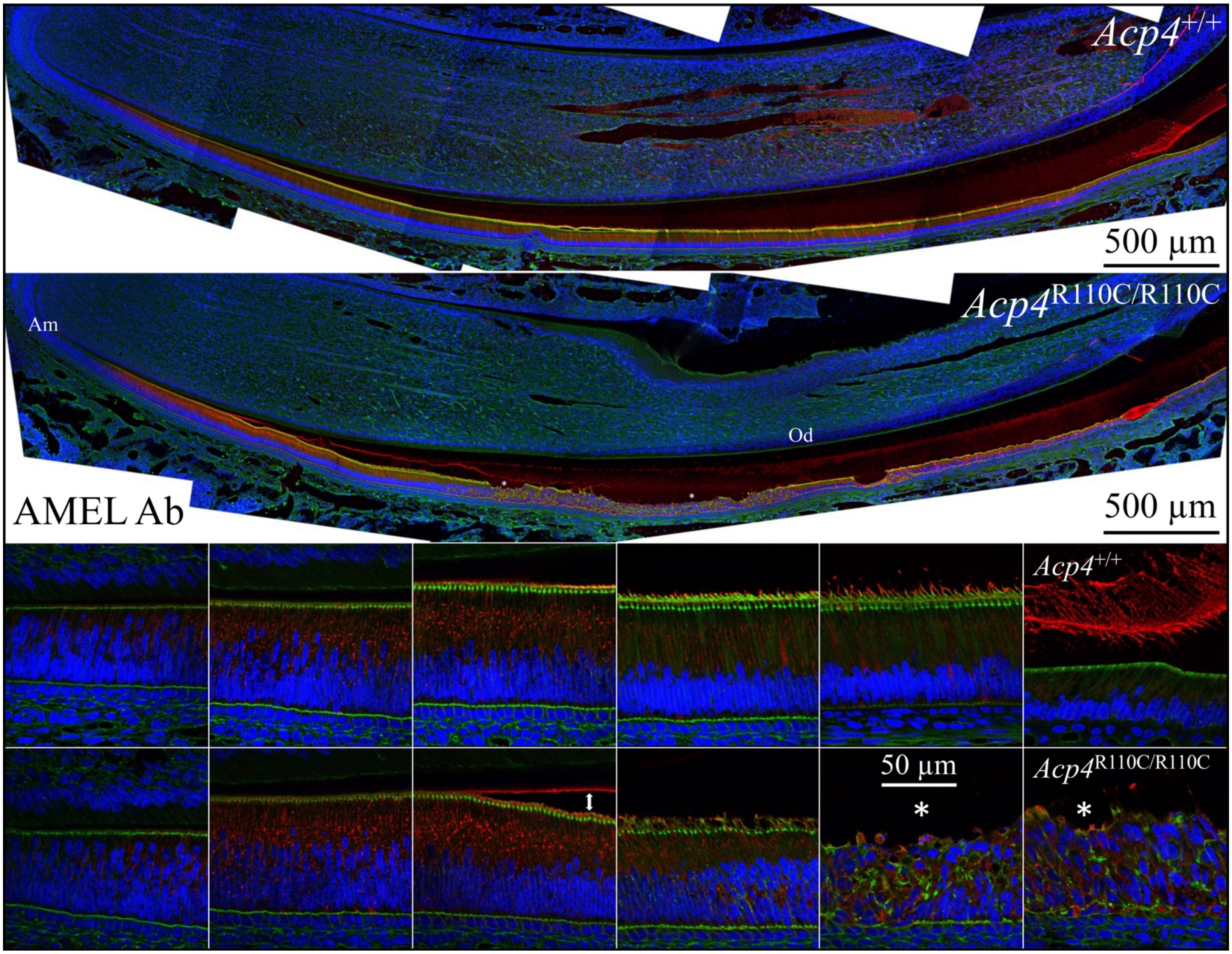

Figure S18. Immunohistochemistry of AMEL in *Acp4*$^{+/+}$ and *Acp4*$^{R110C/R110C}$ D12 Mandibular Incisors. ß-actin is labeled in green, AMEL (amelogenin) in red, and nuclei in blue. High magnification panels are shown at the bottom. At the left, the cells of the mouse incisor are differentiating into ameloblasts (Am) and odontoblasts (Od). In the *Acp4*$^{+/+}$ incisor, AMEL reactivity is evident in secretory stage ameloblasts and enamel matrix. Specifically, the signal appears in secretory pathways inside ameloblasts, in the matrix associated with Tomes processes and deeper into the enamel layer. In the *Acp4*$^{R110C/R110C}$ incisor, the ameloblasts appear to be normal in the first three high magnification panels (pre-secretory stage), although the ameloblast sheet separates from the matrix (possibly a post-mortem artifact) midway through the third high magnification panel (double sided arrow), with most of the AMEL staying within the matrix and the ß-actin staying with the cells. Noticeably, the AMEL-positive vesicles in *Acp4*$^{R110C/R110C}$ ameloblasts appear to be more than those in *Acp4*$^{+/+}$ ameloblasts. The last two panels show areas of complete ameloblast and enamel organ breakdown in *Acp4*$^{R110C/R110C}$ incisor (asterisks).

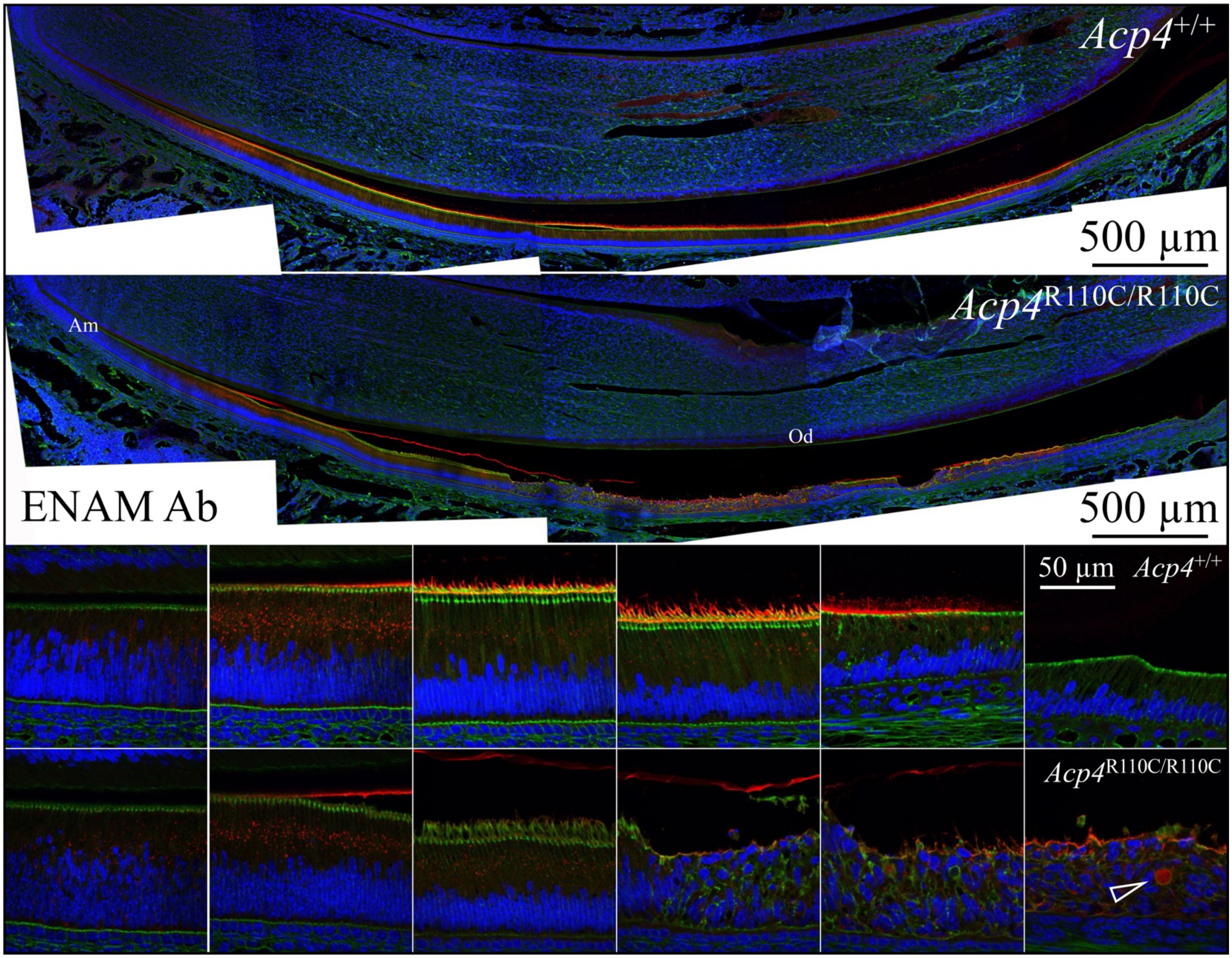

Figure S19. Immunohistochemistry of ENAM in *Acp4*$^{+/+}$ and *Acp4*$^{R110C/R110C}$ D12 Mandibular Incisors. ß-actin is labeled in green, ENAM (enamelin) in red, and nuclei in blue. In both *Acp4*$^{+/+}$ and *Acp4*$^{R110C/R110C}$ incisors, ENAM immunoreaction specifically appears in Golgi apparatus and secretory vesicles of secretory stage ameloblasts, in the matrix associated with Tomes' processes and deeper into the enamel layer. However, the reactivity is weaker in secretory vesicles and around Tomes' processes of *Acp4*$^{R110C/R110C}$ ameloblasts compared to *Acp4*$^{+/+}$. The last three panels show areas of complete ameloblast breakdown in *Acp4*$^{R110C/R110C}$ incisor. At maturation stage (the last panel), while *Acp4*$^{+/+}$ ameloblasts and enamel matrix show no ENAM reactivity, the signal can be detected in *Acp4*$^{R110C/R110C}$ enamel organ epithelium, which has gone pathological, and particularly at ectopic mineral nodules (arrowhead) seen in H&E staining (Fig. 8).

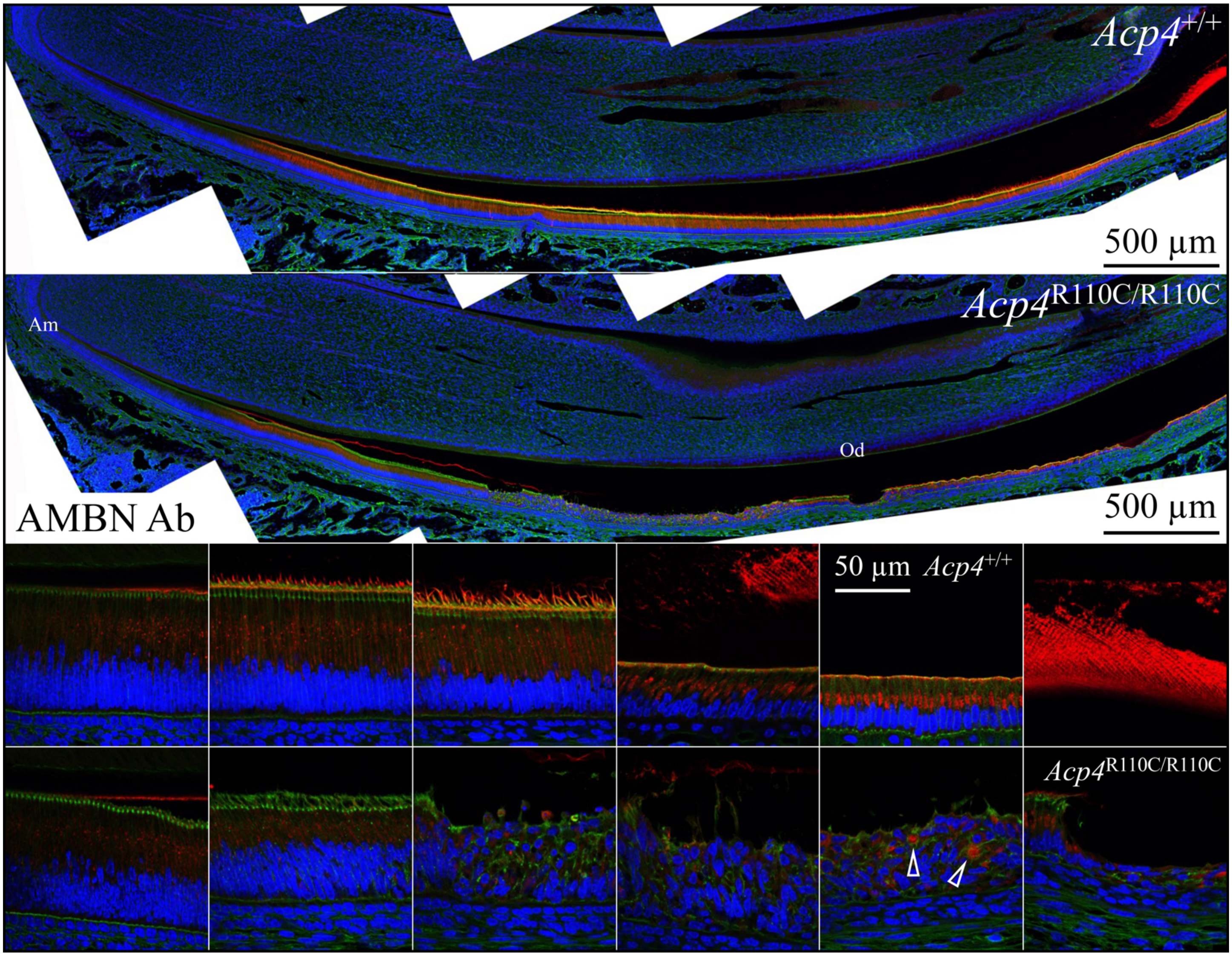

Figure S20. Immunohistochemistry of AMBN in *Acp4*$^{+/+}$ and *Acp4*$^{R110C/R110C}$ D12 Mandibular Incisors. ß-actin is labeled in green, AMBN (ameloblastin) in red, and nuclei in blue. Similar to ENAM signal, AMBN immunoreaction is detected in the Golgi apparatus and secretory vesicles of secretory stage ameloblasts, in the matrix associated with Tomes' processes and deeper into the enamel layer. However, the signal intensity is much weaker in the *Acp4*$^{R110C/R110C}$ incisor comparted to *Acp4*$^{+/+}$, particularly around Tomes' processes. The last four panels show areas of gradual ameloblast breakdown in *Acp4*$^{R110C/R110C}$ incisor. At maturation stage (the last three panel), while *Acp4*$^{+/+}$ ameloblasts show AMBN reactivity at supranuclear region and the distal membrane, the signal in *Acp4*$^{R110C/R110C}$ incisor appears sporadically in disordered enamel organ epithelium and ectopic mineral nodules (arrowhead) seen in H&E staining (Fig. 8).

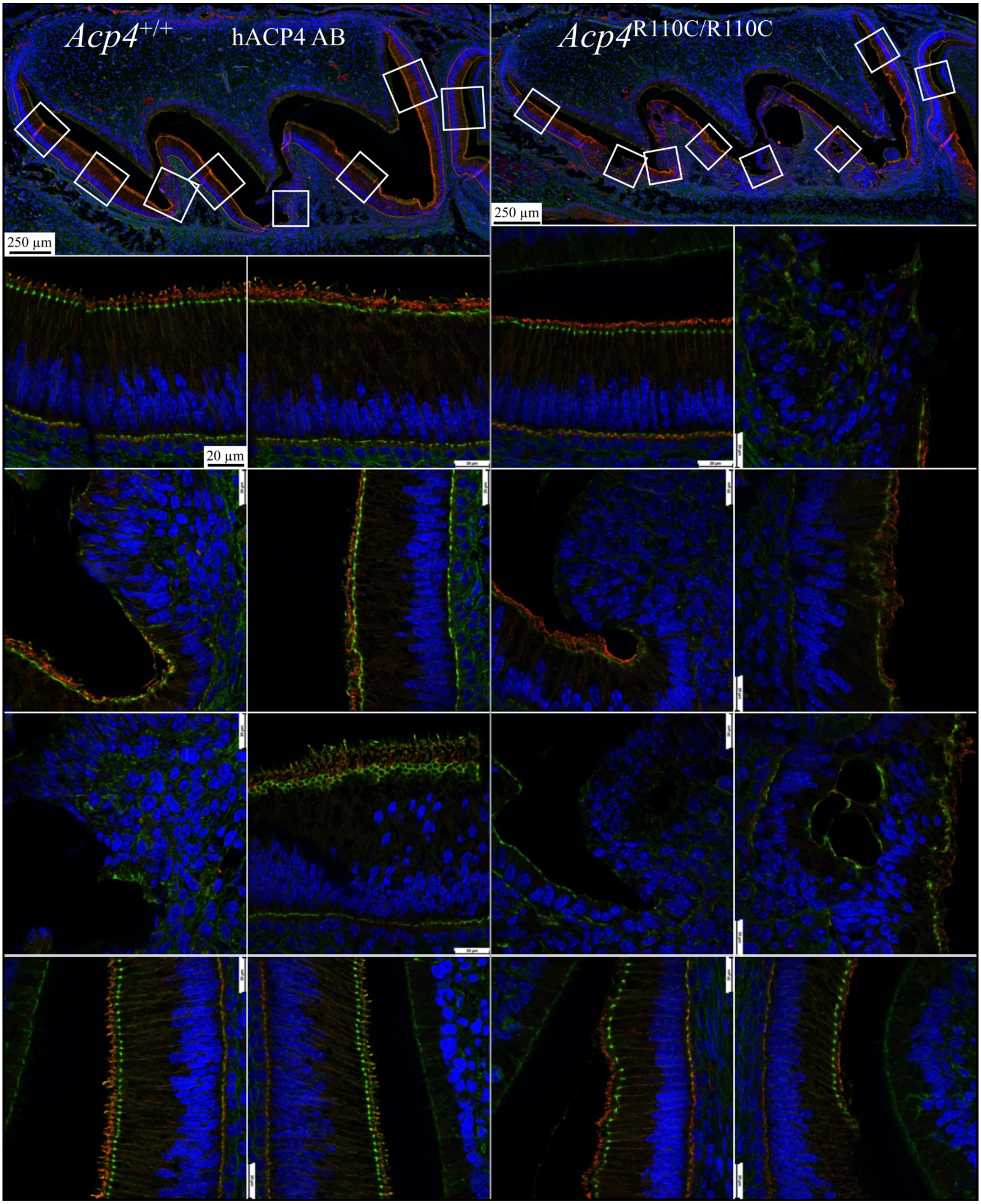

Figure S21. Immunohistochemistry of ACP4 in *Acp4*$^{+/+}$ and *Acp4*$^{R110C/R110C}$ D5 Maxillary First Molars. ß-actin is labeled in green, hACP4 (orb101887) Ab in red, and nuclei in blue. ACP4 immunoreaction in *Acp4*$^{+/+}$ and *Acp4*$^{R110C/R110C}$ molars appeared to be comparable with the most intense signal detected in the Tomes' process, distal to the ß-actin staining.
