# Supplementary material for: Enamel defects in Acp4R110C/R110C mice and human ACP4 mutations
Source: Sci Rep. 2022 Oct 1;12:16477. doi: 10.1038/s41598-022-20684-9 (PMC9526733; doi:10.1038/s41598-022-20684-9)
Supplement: Supplementary file 2 — Supplementary Information 2. [file 41598_2022_20684_MOESM2_ESM.pdf]

Enamel Defects in *Acp4*$^{R110C/R110C}$ Mice and Human *ACP4* Mutations

Tian Liang[1], Shih-Kai Wang[2,3], Charles Smith[1,4], Hong Zhang[1], Yuanyuan Hu[1],
Figen Seymen[5], Mine Koruyucu[6], Yelda Kasimoglu[6], Jung-Wook Kim[7,8],
Chuhua Zhang[1], Thomas L. Saunders[9], James P. Simmer[1]*, and Jan C-C. Hu[1]

*There were equal contributions from Tian Liang and Shih-Kai Wang and both should be considered to be first authors.

Affiliations

[1]Department of Biologic and Materials Sciences, University of Michigan School of Dentistry, 1210 Eisenhower Place, Ann Arbor, MI 48108, USA.

[2]Department of Dentistry, National Taiwan University School of Dentistry, No. 1, Changde St., Zhongzheng Dist., Taipei City 100, Taiwan.

[3]Department of Pediatric Dentistry, National Taiwan University Children's Hospital, No. 8, Zhongshan S. Rd., Zhongzheng Dist., Taipei City 100, Taiwan.

[4]Department of Anatomy & Cell Biology, Faculty of Medicine & Health Sciences, McGill University, Montreal, QC, Canada

[5]Department of Pedodontics, Faculty of Dentistry, Altinbas University, Istanbul, 34147, Turkey.

[6]Department of Pedodontics, Faculty of Dentistry, Istanbul University, Istanbul, 34116, Turkey

[7]Department of Molecular Genetics & Dental Research Institute, School of Dentistry, Seoul National University, Seoul 03080, Republic of Korea.

[8]Department of Pediatric Dentistry & Dental Research Institute, School of Dentistry, Seoul National University, Seoul 03080, Republic of Korea.

[9]Department of Internal Medicine, Division of Molecular, Medicine and Genetics, University of Michigan Medical School, Ann Arbor, MI 48109, USA.

Contents of Supplemental Data File 2

Figure S22. FIB-bSEM 5000x montages of longitudinally sectioned *Acp4*$^{R110C/R110C}$ mouse mandibular incisors showing secretory stage ameloblasts at progressively later stages of amelogenesis.

Figure S23A. *Acp4*$^{R110C/R110C}$ incisor where odontoblasts start forming predentin (levels 0.673–0.785; left) and further incisally (levels 0.694–0.890; right) where the basement membrane is degraded and ameloblasts (Am) send finger-like processes into the predentin matrix.

Figure S23B. *Acp4*$^{R110C/R110C}$ incisor longitudinal segment covering the epithelial-mesenchymal interface at levels 0.673–0.785 magnified x35000x.

Figure S23C. *Acp4*$^{R110C/R110C}$ incisor longitudinal segment covering the epithelial-mesenchymal interface at levels 0.694–0.890 magnified x35000.

Figure S24A. *Acp4*$^{R110C/R110C}$ incisor longitudinal segments (levels 0.89–1.00; left) and further incisally (levels 1.00–1.110; right) covering the onset of dentin mineralization, which is initially observed as islands of mineral, often within collagen fibers, that expand and coalesce into a continuous mineral layer that quickly achieves a high mineral density and continues to expand along an irregular mineralization front on the odontoblast side of a predentin layer at the same rate that more predentin is added.

Figure S24B. *Acp4*$^{R110C/R110C}$ incisor longitudinal segment (levels 0.89-1.00 magnified x35000) covering the onset of dentin mineralization and its expansion into a continuous, highly mineralized tissue in close proximity to the ameloblast distal membrane.

Figure S24C. *Acp4*$^{R110C/R110C}$ incisor longitudinal segment (levels 1.00-1.11 magnified x35000) covering the onset of enamelformation, which is delayed and does not progress normally in *Acp4*$^{R110C/R110C}$ mice.

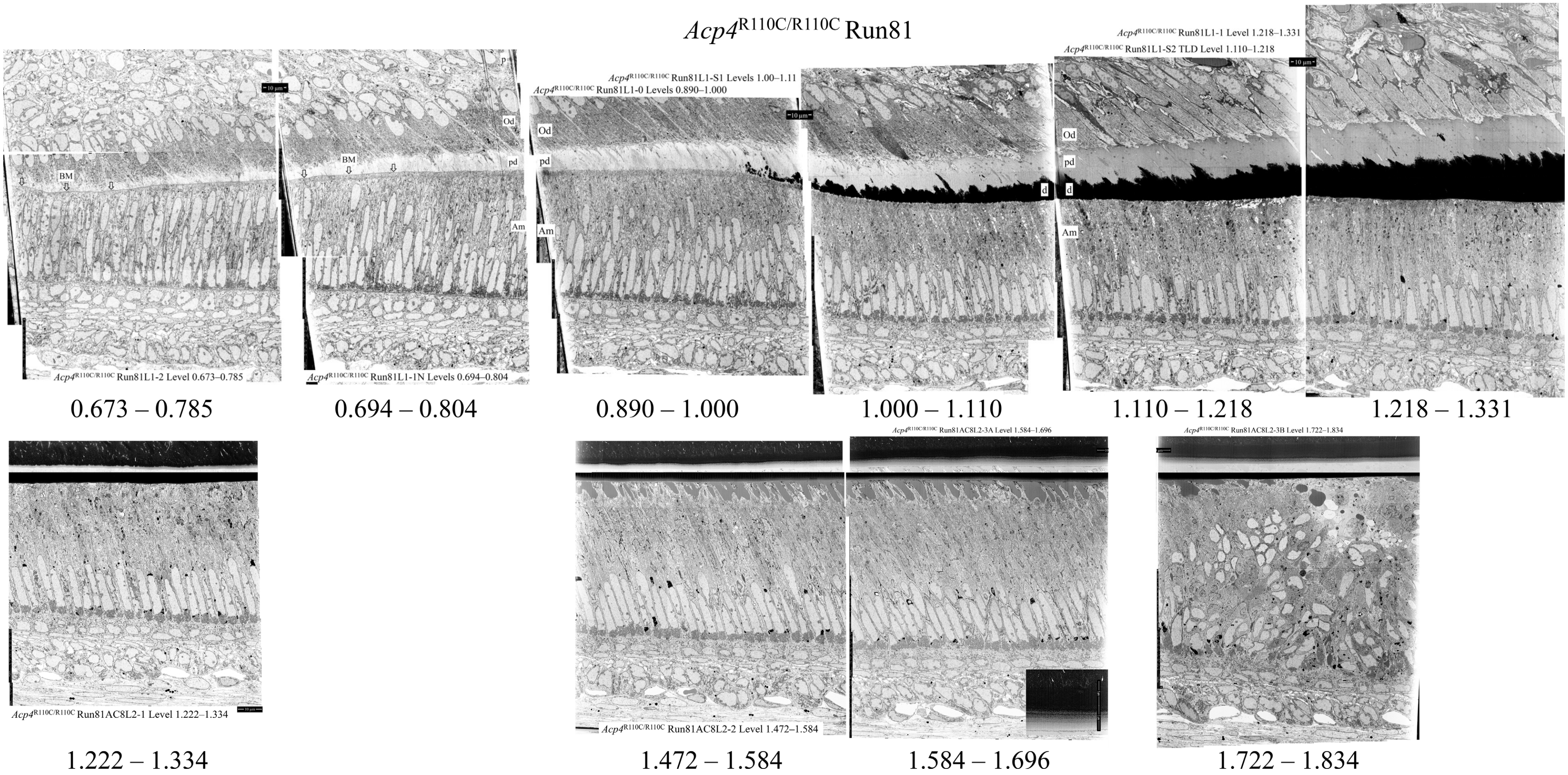

Fig. S22. FIB-bSEM 5000x montages of longitudinally sectioned *Acp4*$^{R110C/R110C}$ mouse mandibular incisors showing secretory stage ameloblasts at progressively later stages of amelogenesis (left to right). The top and bottom panels show montages from two different *Acp4*$^{R110C/R110C}$ mandibular incisors. The "levels" (in mm) allow comparison of images from different mice from the same location on the incisor. Level 1.0 is the onset of enamel formation, which is also 1 mm curvilinear distance from the beginning of the apical loop. FIB-bSEM montages of these *Acp4*$^{R110C/R110C}$ incisors are shown at multiple magnifications (x5000, x10000, x20000, and x35000) in the following figures and document the disturbed growth of aplastic enamel in the knockin mice until the ameloblast layer itself becomes pathological. Ultrastructurally, pathology is first evident starting at the very onset of amelogenesis (level 1.0), where fluid and debris accumulating the in extracellular matrix cause disturbances in enamel ribbon deposition. ***Key:*** Am, ameloblast; d, dentin; Od, odontoblast; p, pulp; pd, predentin; si, stratum intermedium.

In a 7-week wild-type mouse incisor the enamel grows to 121 μm in thickness from the DEJ to the enamel surface over a distance of ~2 mm. It starts in patches at level 1.0 and linearly adds 0.605 μm enamel thickness for each 10 μm step incisally. The enamel achieves its final thickness at level 3 and transitions into the maturation stage where the enamel layer as a whole does not expand further, but hardens as the mineral ribbons deposited during the secretory stage grow in width and thickness. Because of this linear rate of growth, it is possible to calculate what the thickness of normal enamel would be at comparable locations on the incisor.

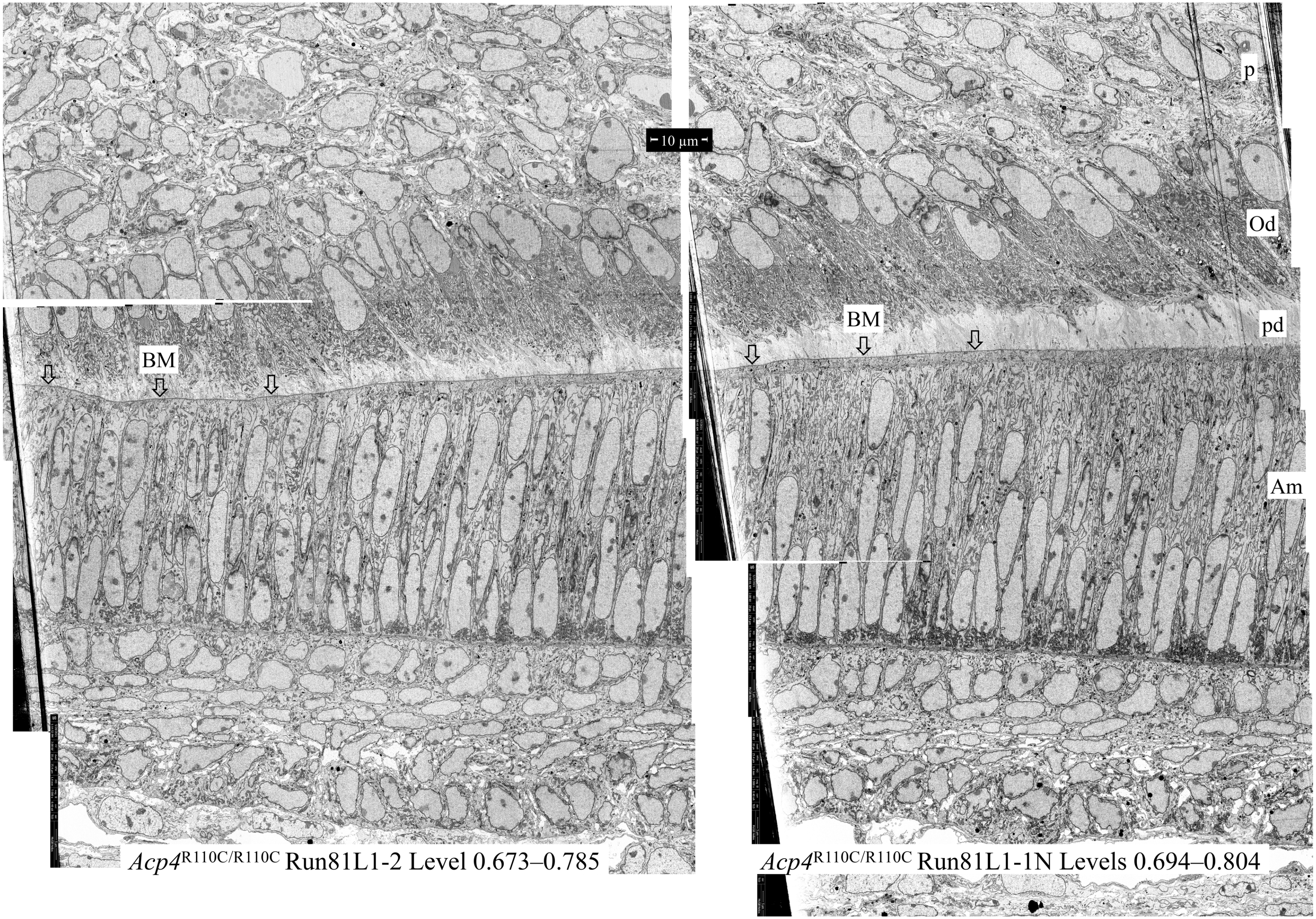

Figure S23A. *Acp4*$^{R110C/R110C}$ incisor where odontoblasts start forming predentin (levels 0.673–0.785; left) and further incisally (levels 0.694–0.890; right) where the basement membrane is degraded and ameloblasts (Am) send finger-like processes into the predentin matrix. Note the abundance of mitochondria at the proximal (lower) end of the ameloblasts in the supranuclear region. Arrows show the position of the basement membrane (BM) that anchors differentiating ameloblasts (Am) to the underlying predentin (pd).

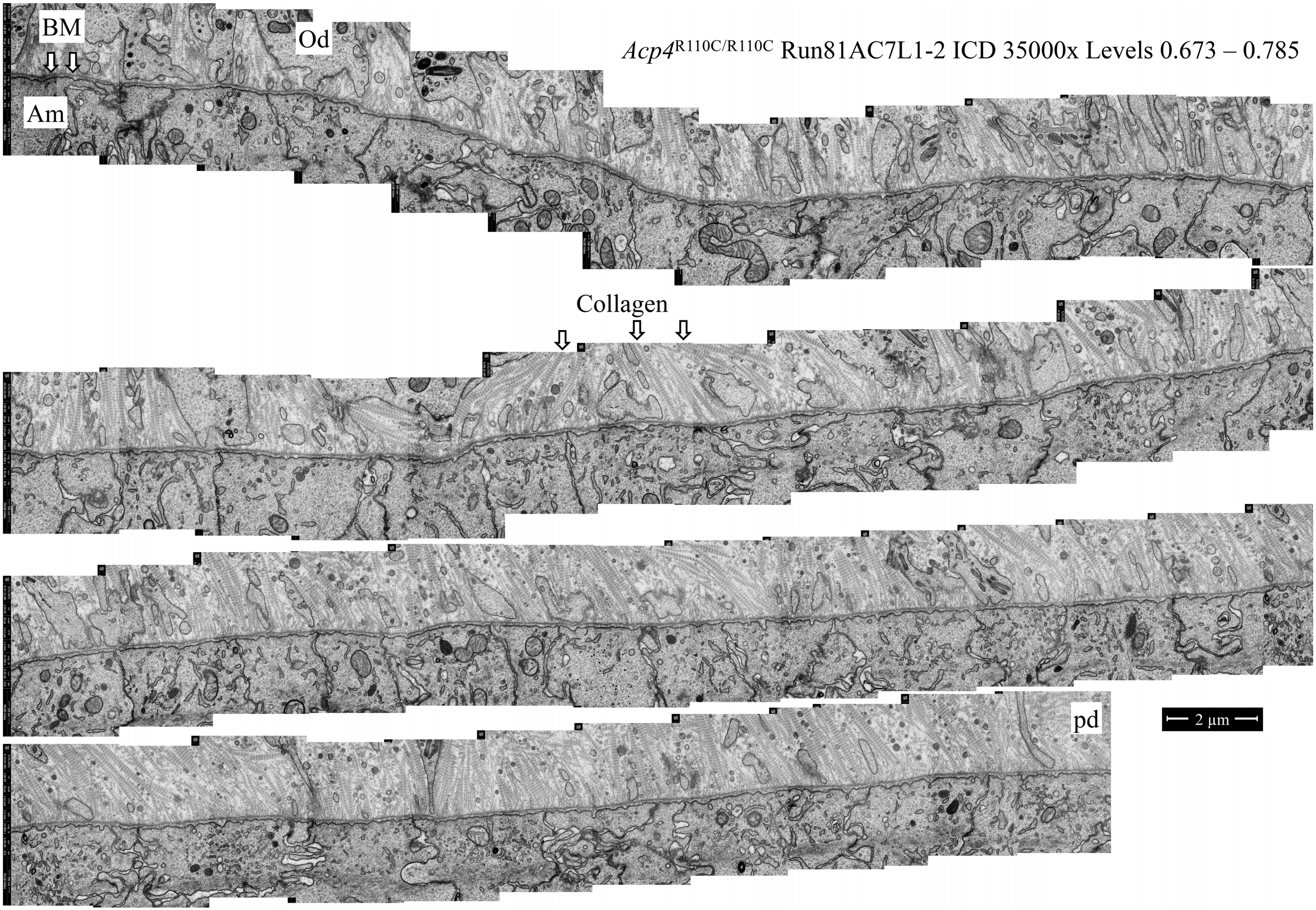

Figure S23B. *Acp4*[R110C/R110C] incisor longitudinal segment covering the epithelial-mesenchymal interface at levels 0.673–0.785 magnified x35000x. The segment starts near the apical loop of the mandibular incisor and progresses incisally. Odontoblasts (Od) are depositing a predentin (pd) matrix containing progressively more abundant, oriented collagen fibers as they recede away from the underlying ameloblasts (Am), which are anchored firmly to a conspicuous basement membrane (BM).

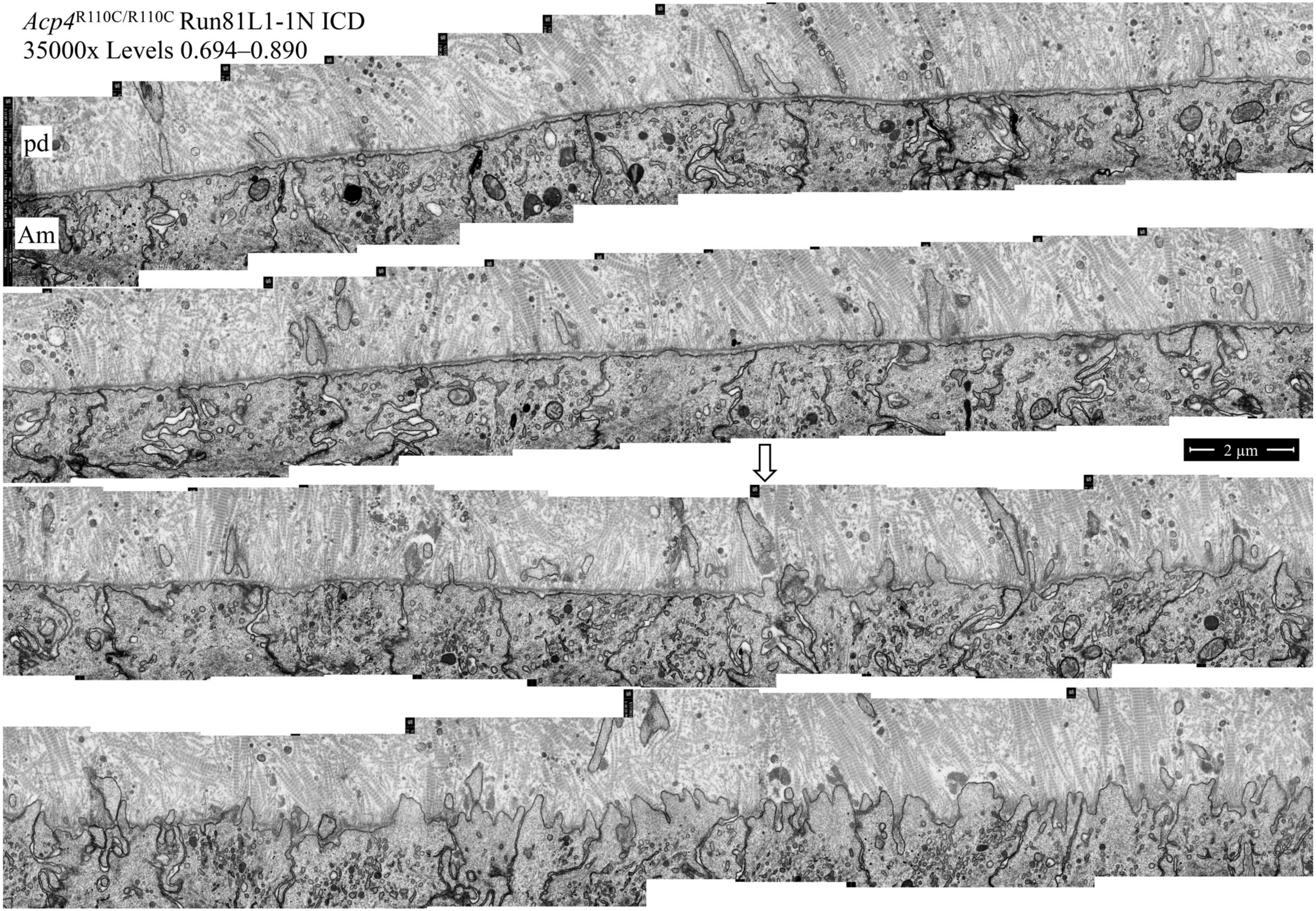

Figure S23C. *Acp4*$^{R110C/R110C}$ incisor longitudinal segment covering the epithelial-mesenchymal interface at levels 0.694–0.890 magnified x35000. The segment starts with an intact basement membrane (BM). The first breach of the basement membrane by an ameloblast process is marked by an arrow. The lamina densa of the BM accumulates along the ameloblast distal membrane between the processes, apparently drawing the still attached collagen fibers into a more intimate association with ameloblasts.

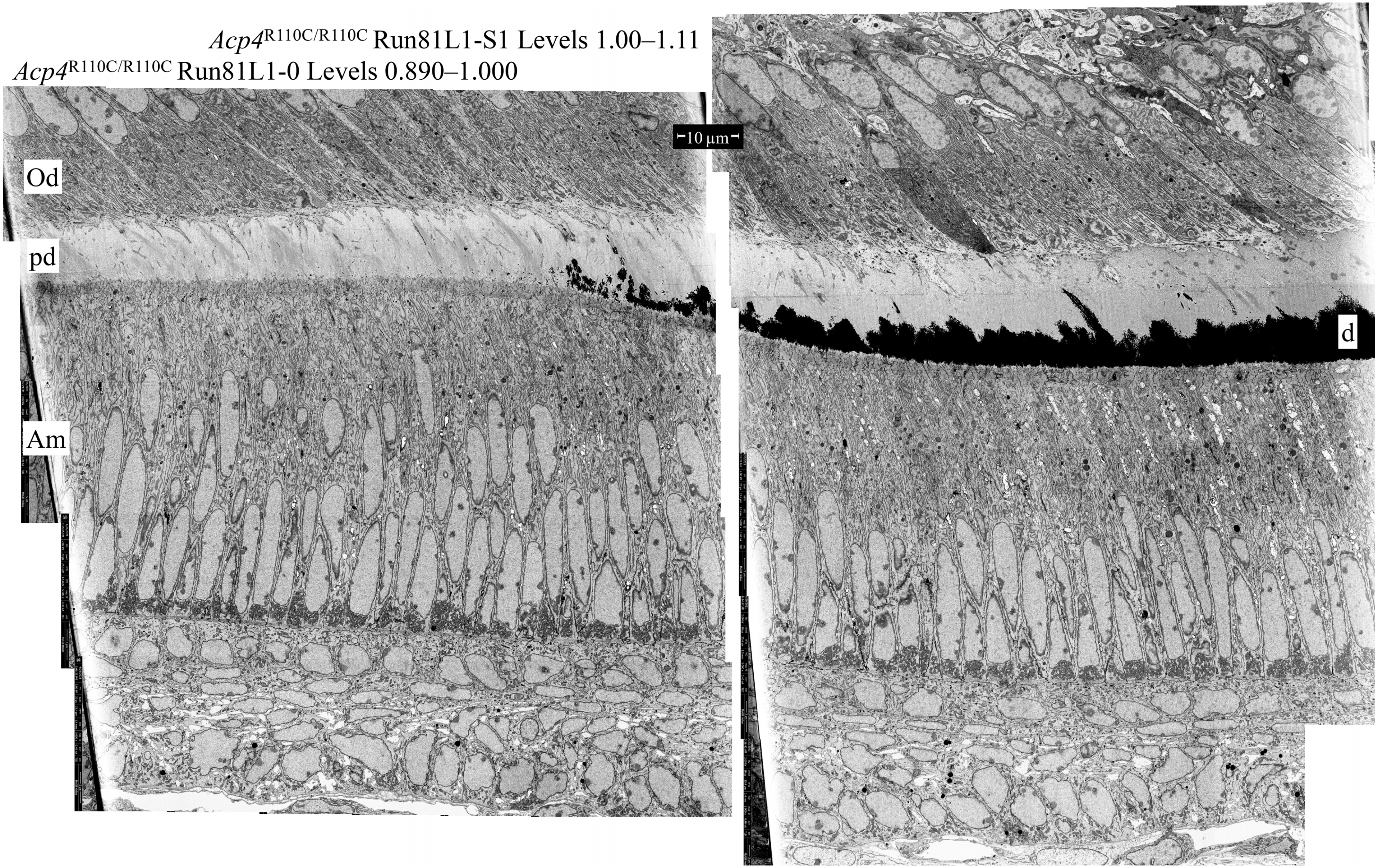

Figure S24A. *Acp4*$^{R110C/R110C}$ incisor longitudinal segments (levels 0.89–1.00; left) and further incisally (levels 1.00–1.110; right) covering the onset of dentin mineralization, which is initially observed as islands of mineral, often within collagen fibers, that expand and coalesce into a continuous mineral layer that quickly achieves a high mineral density and continues to expand along an irregular mineralization front on the odontoblast side of a predentin layer at the same rate that more predentin is added. In wild-type mice the onset of initial enamel mineral ribbon formation occurs at level 1.0 and is followed by formation of Tomes processes that establish rod/interrod organization (Fig. 9A-9C).

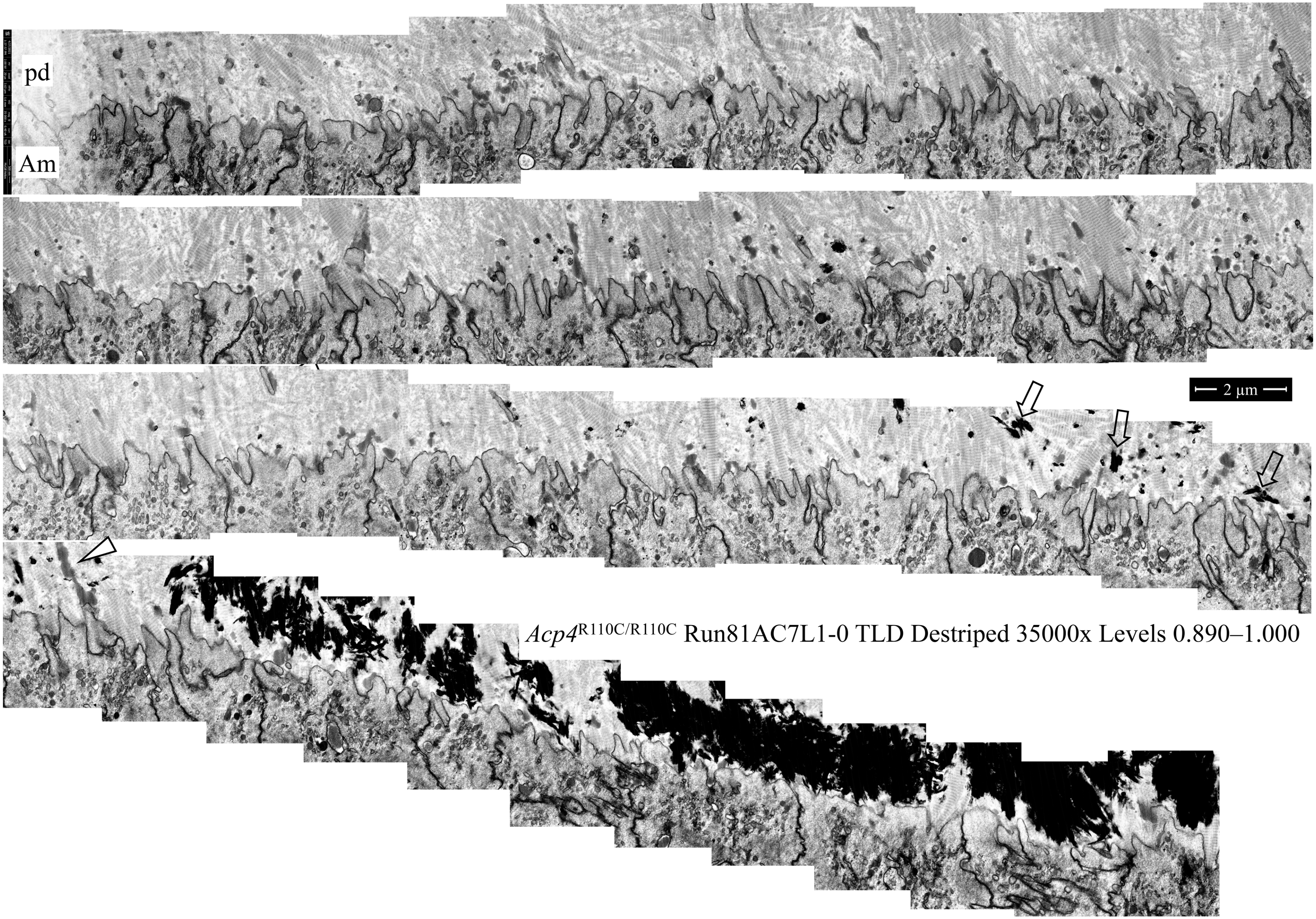

Figure S24B. *Acp4*$^{R110C/R110C}$ incisor longitudinal segment (levels 0.89-1.00 magnified x35000) covering the onset of dentin mineralization and its expansion into a continuous, highly mineralized tissue in close proximity to the ameloblast distal membrane. The triangle points to accumulations of amelogenin, often associated with odontoblastic processes. Arrows point to islands of mineral, often associated with collagen fibers. The panel ends at level 1.0, the typical onset of the first appearance of enamel mineral ribbons on the surface of dentin.

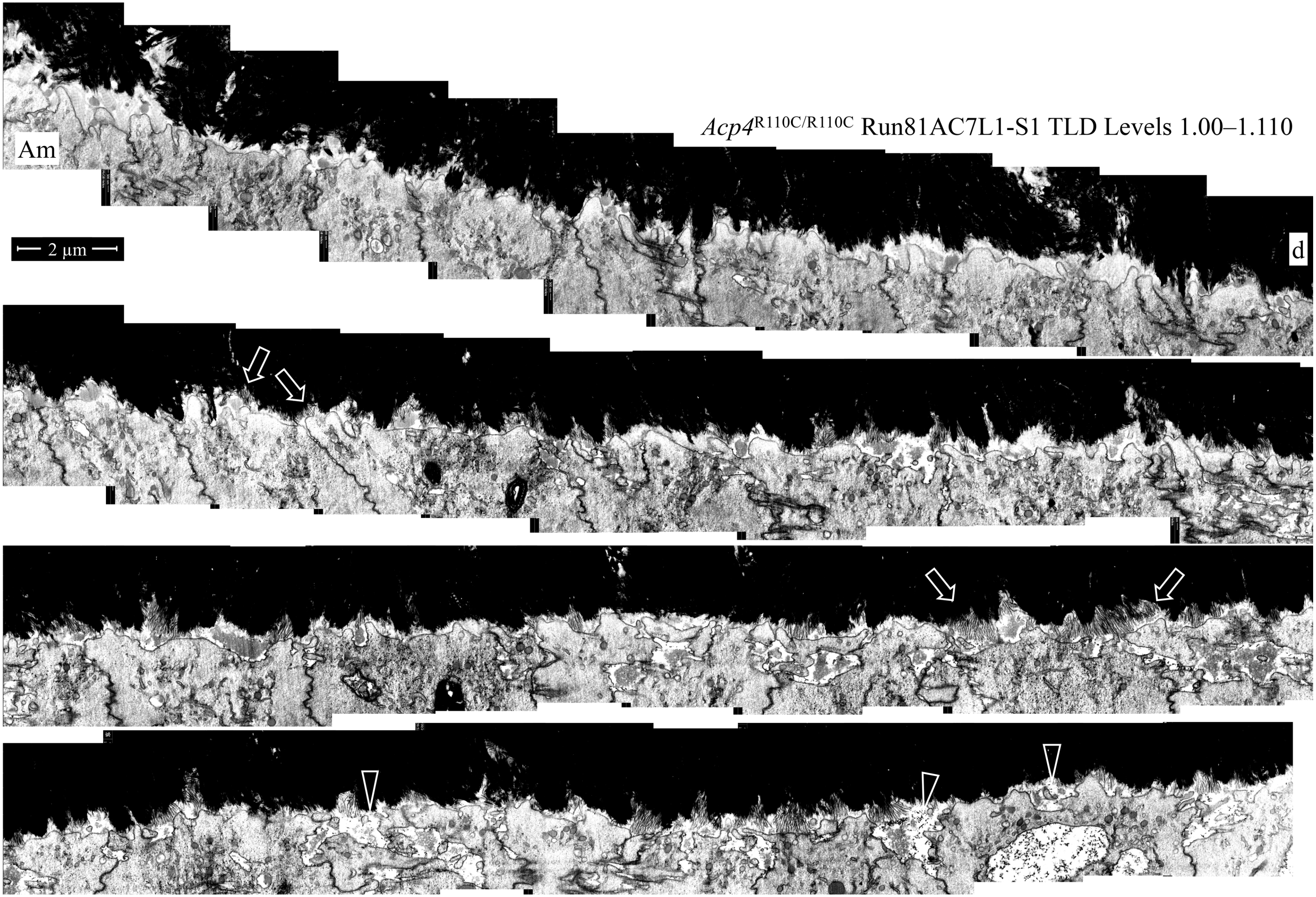

Figure S24C. *Acp4*$^{R110C/R110C}$ incisor longitudinal segment (levels 1.00-1.11 magnified x35000) covering the onset of enamelformation, which is delayed and does not progress normally in *Acp4*$^{R110C/R110C}$ mice. Arrows point to islands of mineral ribbons growing on mineralized dentin. Triangles point to extracellular fluid and debris not observed in wild-type mice. Our original hypothesis that ACP4 is a lysosomal phosphatase and loss of its function would cause a lysosomal storage disease are not consistent with these observations.
