# Supplementary material for: Enamel defects in Acp4R110C/R110C mice and human ACP4 mutations
Source: Sci Rep. 2022 Oct 1;12:16477. doi: 10.1038/s41598-022-20684-9 (PMC9526733; doi:10.1038/s41598-022-20684-9)
Supplement: Supplementary file 3 — Supplementary Information 3. [file 41598_2022_20684_MOESM3_ESM.pdf]

Enamel Defects in *Acp4*$^{R110C/R110C}$ Mice and Human *ACP4* Mutations

Tian Liang[1], Shih-Kai Wang[2,3], Charles Smith[1,4], Hong Zhang[1], Yuanyuan Hu[1], Figen Seymen[5], Mine Koruyucu[6], Yelda Kasimoglu[6], Jung-Wook Kim[7,8], Chuhua Zhang[1], Thomas L. Saunders[9], James P. Simmer[1]*, and Jan C-C. Hu[1]

*There were equal contributions from Tian Liang and Shih-Kai Wang and both should be considered to be first authors.

Affiliations

[1]Department of Biologic and Materials Sciences, University of Michigan School of Dentistry, 1210 Eisenhower Place, Ann Arbor, MI 48108, USA.

[2]Department of Dentistry, National Taiwan University School of Dentistry, No. 1, Changde St., Zhongzheng Dist., Taipei City 100, Taiwan.

[3]Department of Pediatric Dentistry, National Taiwan University Children's Hospital, No. 8, Zhongshan S. Rd., Zhongzheng Dist., Taipei City 100, Taiwan.

[4]Department of Anatomy & Cell Biology, Faculty of Medicine & Health Sciences, McGill University, Montreal, QC, Canada

[5]Department of Pedodontics, Faculty of Dentistry, Altinbas University, Istanbul, 34147, Turkey.

[6]Department of Pedodontics, Faculty of Dentistry, Istanbul University, Istanbul, 34116, Turkey

[7]Department of Molecular Genetics & Dental Research Institute, School of Dentistry, Seoul National University, Seoul 03080, Republic of Korea.

[8]Department of Pediatric Dentistry & Dental Research Institute, School of Dentistry, Seoul National University, Seoul 03080, Republic of Korea.

[9]Department of Internal Medicine, Division of Molecular, Medicine and Genetics, University of Michigan Medical School, Ann Arbor, MI 48109, USA.

Contents of Supplemental Data File 3

Figure S25A. *Acp4*$^{R110C/R110C}$ incisor longitudinal segments (levels 1.11–1.218; left) and further incisally (levels 1.218–1.331; right) covering what is normally a period of rapid expansion of the enamel layer by the elongation of rod and interrod enamel mineral ribbons (see Fig. 9).

Figure S25B. *Acp4*$^{R110C/R110C}$ incisor longitudinal segment (levels 1.110–1.218 magnified x35000) showing a location that would normally be covered by an expanding enamel layer. In wild-type mice the distance from the dentin surface (DEJ) to the surface of the growing enamel (enamel thickness) would be, at the top left, 6.6 μm and, at the lower right, 13 μm thick.

Figure S25C. *Acp4*$^{R110C/R110C}$ incisor longitudinal segment (levels 1.218–1.331 magnified x35000) showing a location that in the wild-type incisor would be covered by an expanding enamel layer that at the top left would be 13 μm thick (from the DEJ to the enamel surface) and 20 μm thick at the lower right.

Figure S26A. *Acp4*$^{R110C/R110C}$ incisor longitudinal segments (level 1.222–1.334; left) and further incisally (level 1.584–1.696; right).

Figure S26B. *Acp4*$^{R110C/R110C}$ incisor longitudinal segment (level 1.222–1.334) is from a different *Acp4*$^{R110C/R110C}$ incisor and closely matches the region covered in Fig. S24c.

Figure S26C. *Acp4*$^{R110C/R110C}$ incisor longitudinal segment (level 1.472–1.584) more incisally to cover a location that in the wild-type incisor where the enamel would be between 28.3 to 35 μm thick.

Figure S27A. *Acp4*$^{R110C/R110C}$ incisor longitudinal segments (level 1.584–1.696; left) and further incisally (level 1.722–1.834; right). The 5000x montage on the left is from an *Acp4*$^{R110C/R110C}$ level that in a wild-type mouse would 28.3 μm thick enamel on the left and 41.8 μm on the right.

Figure S27B. *Acp4*$^{R110C/R110C}$ incisor longitudinal segment showing bright images at level 1.584–1.696 magnified x10000x (top), and 20000x (bottom).

Figure S27C. *Acp4*$^{R110C/R110C}$ incisor longitudinal segment showing bright images at level 1.722–1.834 magnified x10000x (top), and 20000x (bottom).

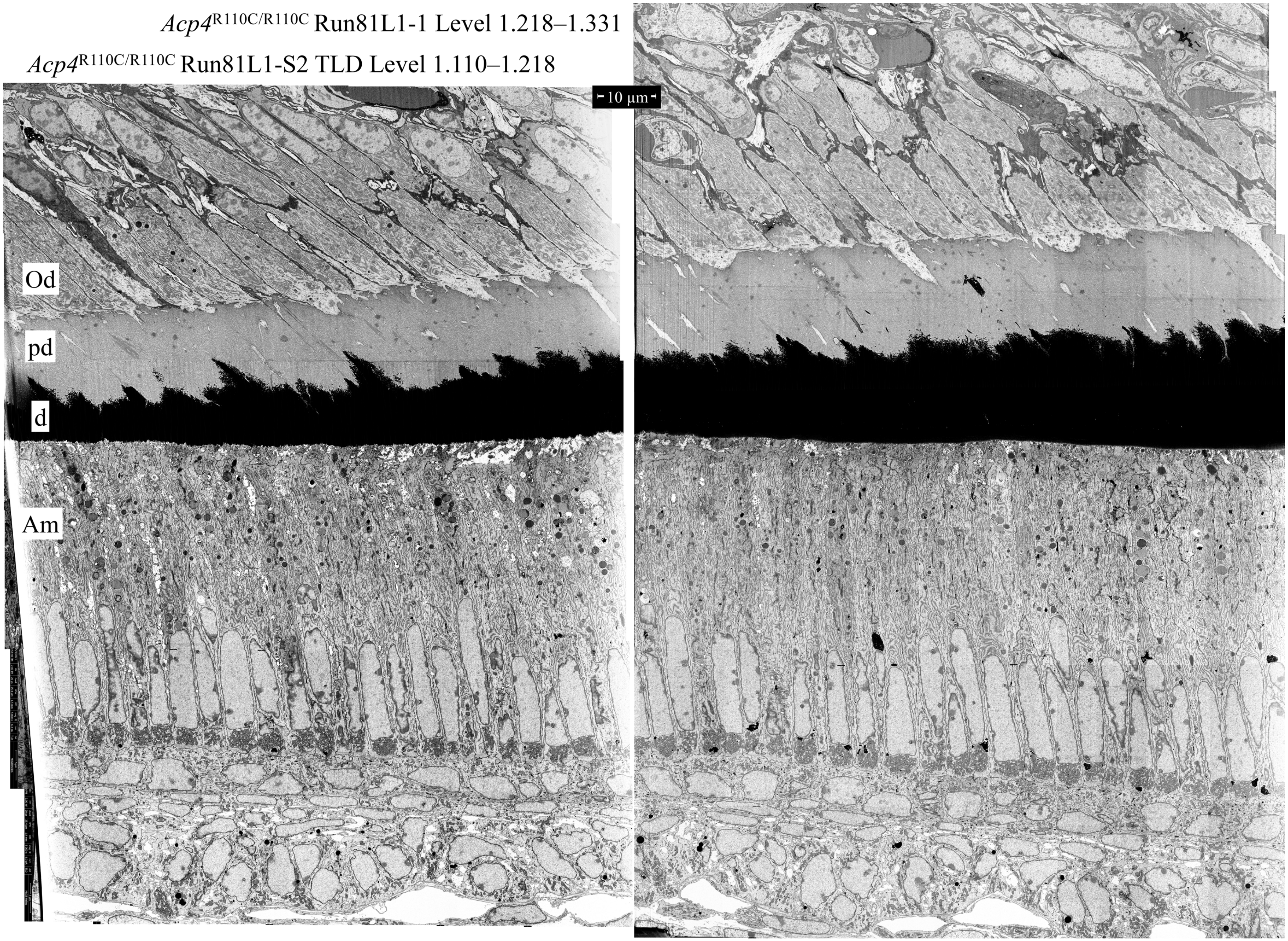

Figure S25A. *Acp4*$^{R110C/R110C}$ incisor longitudinal segments (levels 1.11–1.218; left) and further incisally (levels 1.218–1.331; right) covering what is normally a period of rapid expansion of the enamel layer by the elongation of rod and interrod enamel mineral ribbons (see Fig. 9). The loss of function of ACP4 causes major changes at the distal ends of ameloblasts such that they are unable to support normal appositional growth of the enamel layer and subsequent development of normal functional Tomes processes.

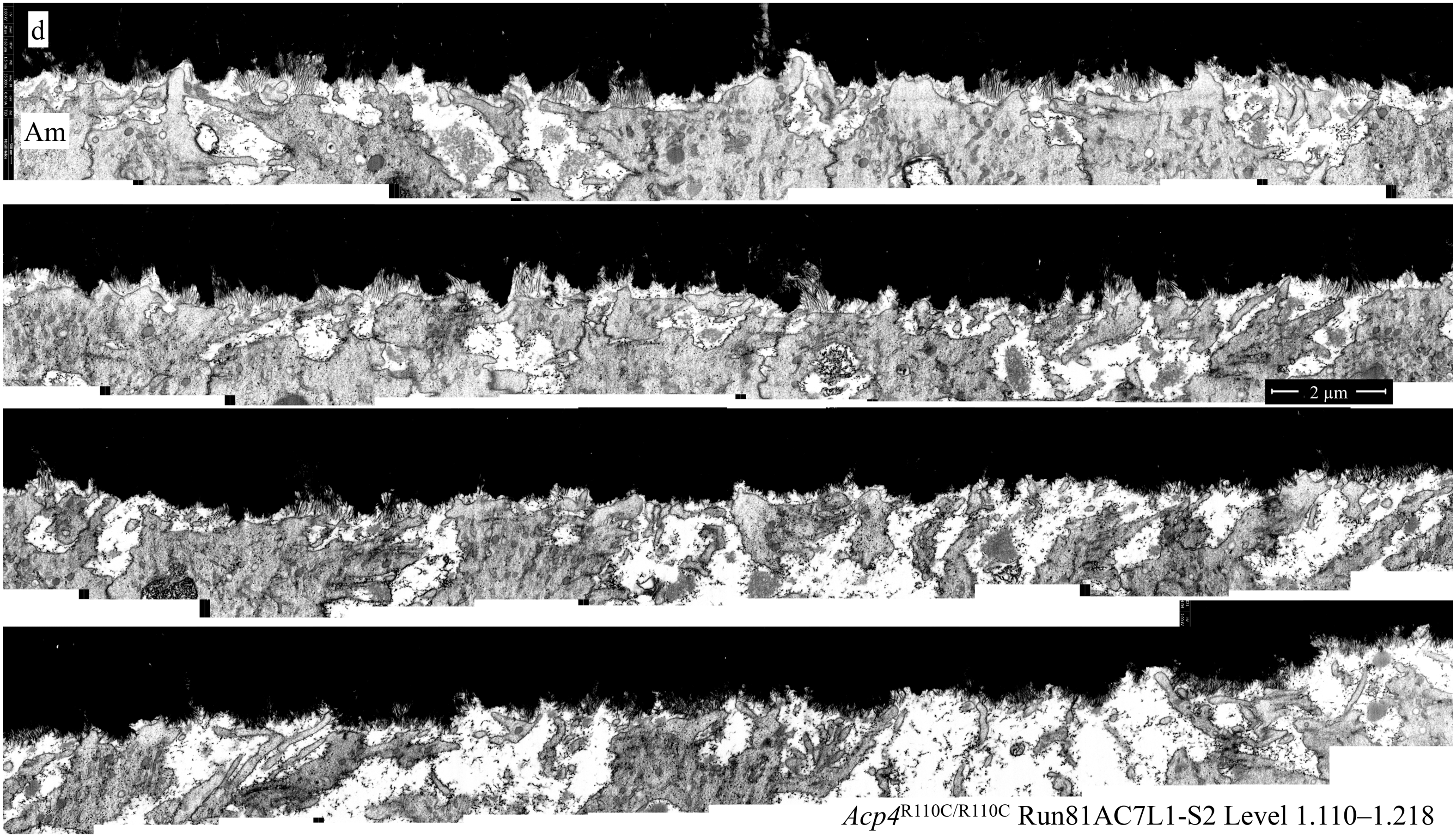

Figure S25B. *Acp4*$^{R110C/R110C}$ incisor longitudinal segment (levels 1.110–1.218 magnified x35000) showing a location that would normally be covered by an expanding enamel layer. In wild-type mice the distance from the dentin surface (DEJ) to the surface of the growing enamel (enamel thickness) would be, at the top left, 6.6 μm and, at the lower right, 13 μm thick. Instead, in the absence of ACP4 phosphatase activity, there are only spiky patches of short mineral ribbons on the surface of dentin and clear, and likely fluid-filled, spaces containing debris covering the highly mineralized dentin surface. Although the ameloblasts themselves retain their shape and polarity, the extracellular matrix is aplastic and Tomes processes fail to develop.

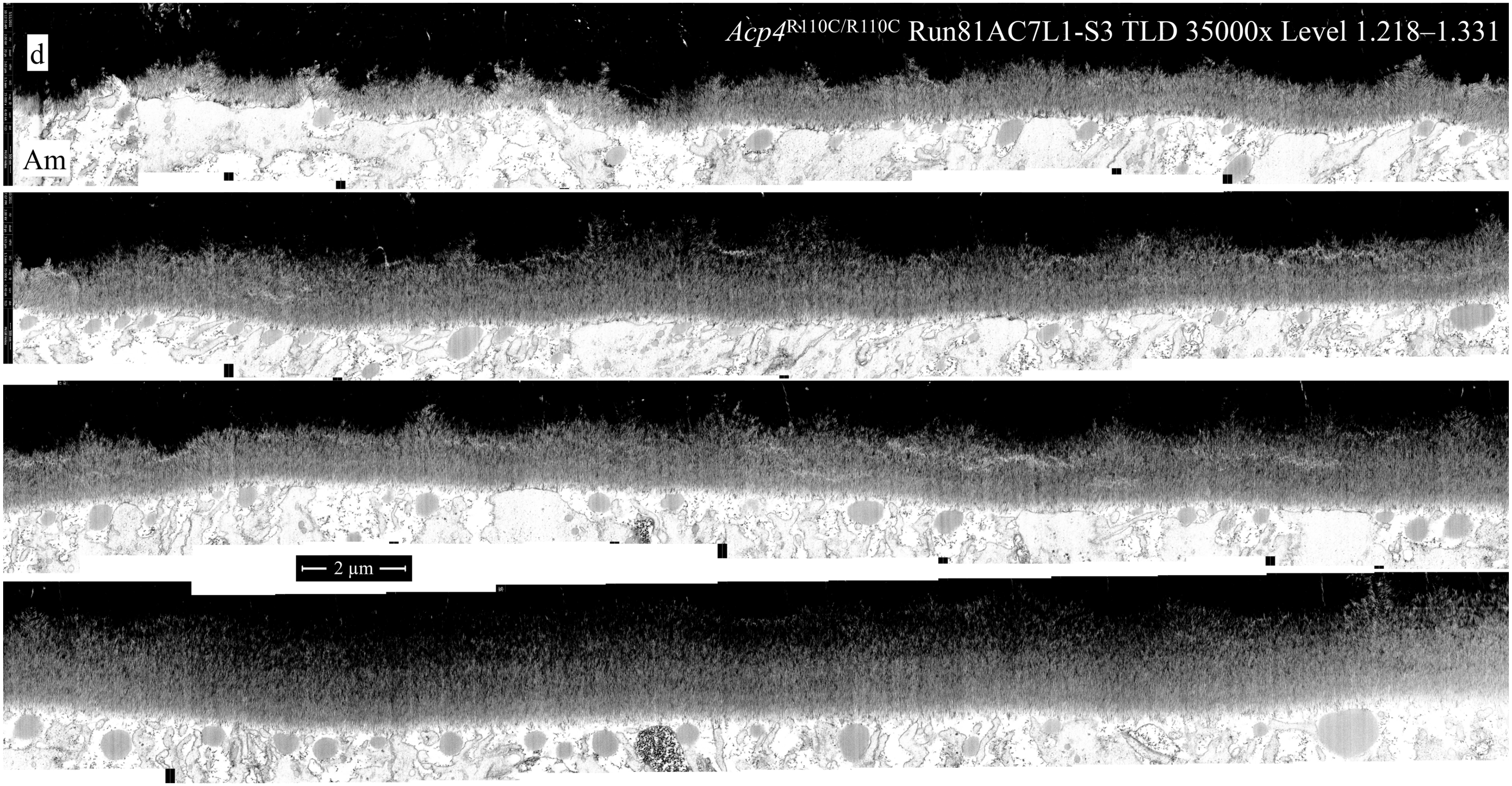

Figure S25C. *Acp4*$^{R110C/R110C}$ incisor longitudinal segment (levels 1.218–1.331 magnified x35000) showing a location that in the wild-type incisor would be covered by an expanding enamel layer that at the top left would be 13 µm thick (from the DEJ to the enamel surface) and 20 µm thick at the lower right. Here, in the absence of ACP4 activity, the hypoplastic mineral showing lines oriented from the dentin surface toward the overlying ameloblast seem to lengthen in layers that mature (become highly mineralized) prematurely, making it increasingly difficult to discern where dentin ends, and enamel begins. These images were taken with greater brightness to discern the aplastic enamel layer, while losing contrast in the cellular layer. The enamel layer in this segment expands from 0.2 µm at the upper left to about 1.5 µm at the lower right. A new feature in this segment is the accumulation of protein droplets superficial to the mineral that are presumed to be comprised mostly of amelogenin within the clear, debris-containing extracellular fluid.

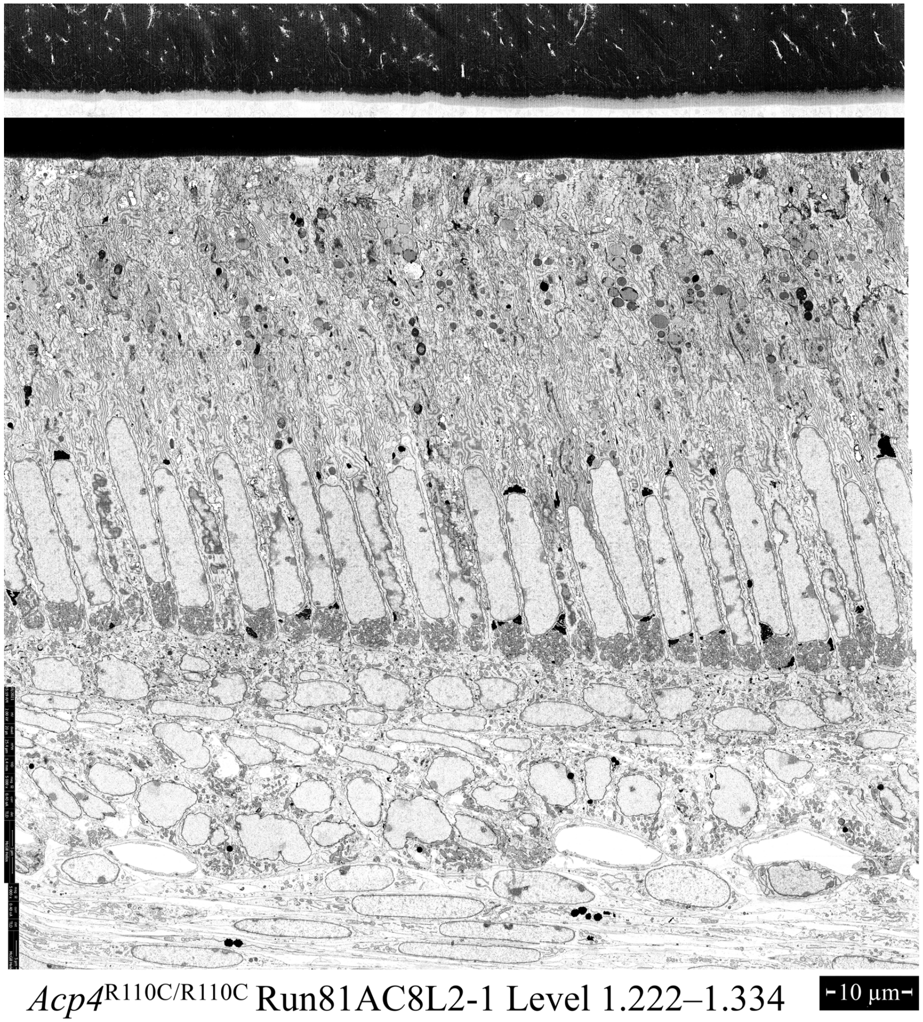

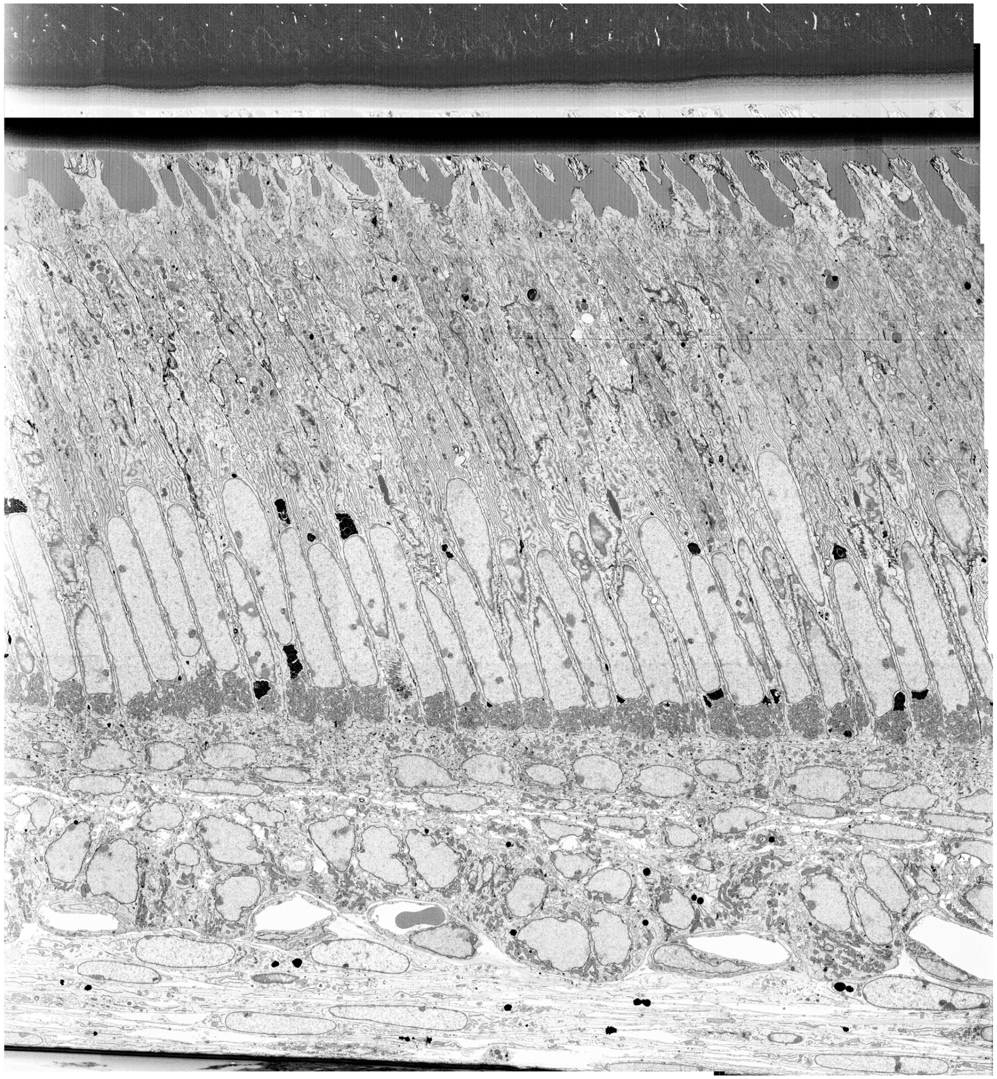

Acp4$^{R110C/R110C}$ Run81AC8L2-1 Level 1.222–1.334

Acp4$^{R110C/R110C}$ Run81AC8L2-2 Level 1.472–1.584

Figure S26A. *Acp4*$^{R110C/R110C}$ incisor longitudinal segments (level 1.222–1.334; left) and further incisally (level 1.584–1.696; right). These images are from a different incisor than the ones shown previously. The montage on the left captures a location comparable to one already shown from the first incisor (at 5000x in Fig. S14a right, and at 35000x in Fig. S14c). This level is characterized by the accumulation of small globs of gray-colored material in the matrix. At normal exposures the *Acp4*$^{R110C/R110C}$ aplastic enamel became prematurely as dense as dentin and became indistinguishable from it. Because of this, from this point forward we show the brighter images of the mineral layer on top and above the normally exposed (black) mineral layer, to allow estimates of the thickness of the aplastic enamel layer to be carried out.

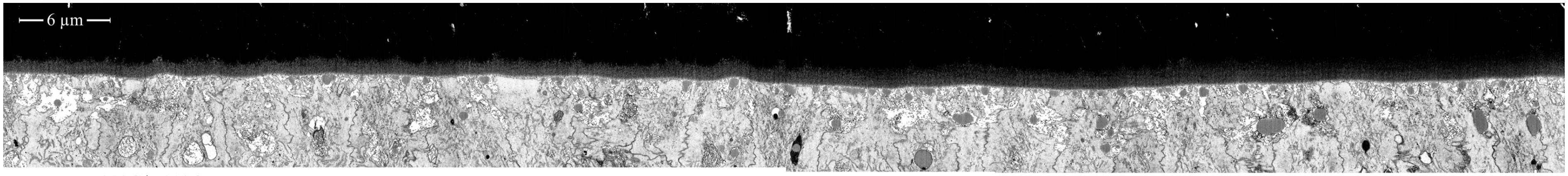

Acp4$^{R110C/R110C}$ TLD Montage 10000x DS Levels 1.222–1.334

Acp4$^{R110C/R110C}$ Run81AC8L2-1 TLD Montage 20000x

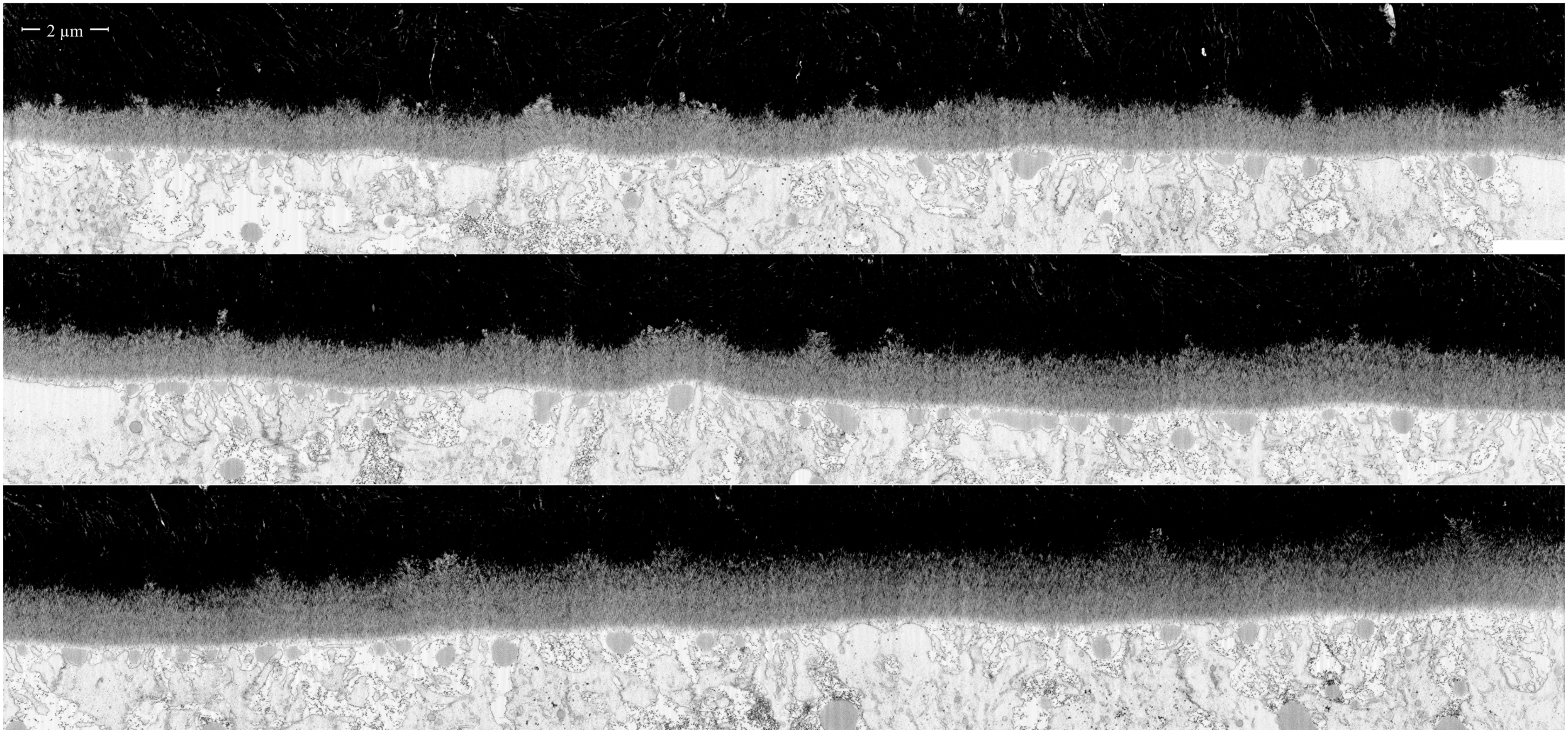

Figure S26B. *Acp4*$^{R110C/R110C}$ incisor longitudinal segment (level 1.222–1.334) is from a different *Acp4*$^{R110C/R110C}$ incisor and closely matches the region covered in Fig. S24c. The panel on top is magnified x10000 and imaged at normal exposure, which best shows cellular detail. Note that the enamel layer is thin (only reaching a thickness of ~1 µm in this segment), but is almost as mineralized as dentin. In a wild-type incisor at this location the enamel layer would be 13 to 20 µm thick and could be readily distinguished from dentin. Below is the x20000 montage of the same segment taken at brighter exposure. As in Fig. S24c showing a different incisor at the same location, the aplastic enamel layer seems to expand outwards in layers (note the horizontal lines in the aplastic enamel) that become highly mineralized prematurely, blurring the boundary between dentin and enamel. There is also a similar accumulation of protein droplets superficial to the mineral.

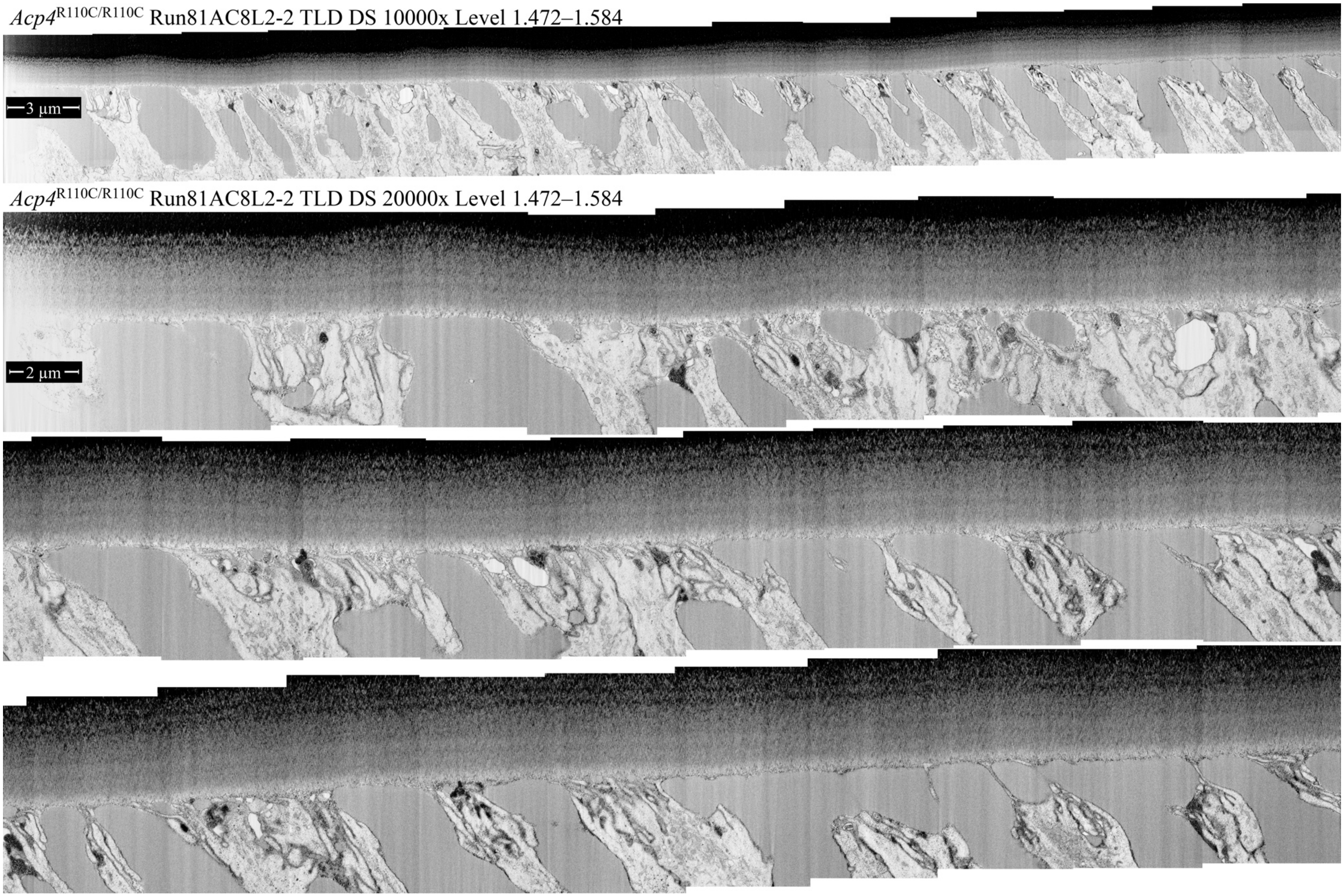

Figure S26C. *Acp4*$^{R110C/R110C}$ incisor longitudinal segment (level 1.472–1.584) more incisally to cover a location that in the wild-type incisor where the enamel would be between 28.3 to 35 µm thick. All images here were taken at increased brightness. In these reverse contrast FIB-bSEM images, areas having greater mineral content are the darkest. The panel on top is magnified x10000, the bottom at x20000. Horizontal lines in the enamel of varying mineral density are evident. The aplastic enamel is maturing from the DEJ toward the surface. Protein accumulations superficial to the mineral have expanded and merged and appear to be stressing the ameloblast attachment to the mineral surface.

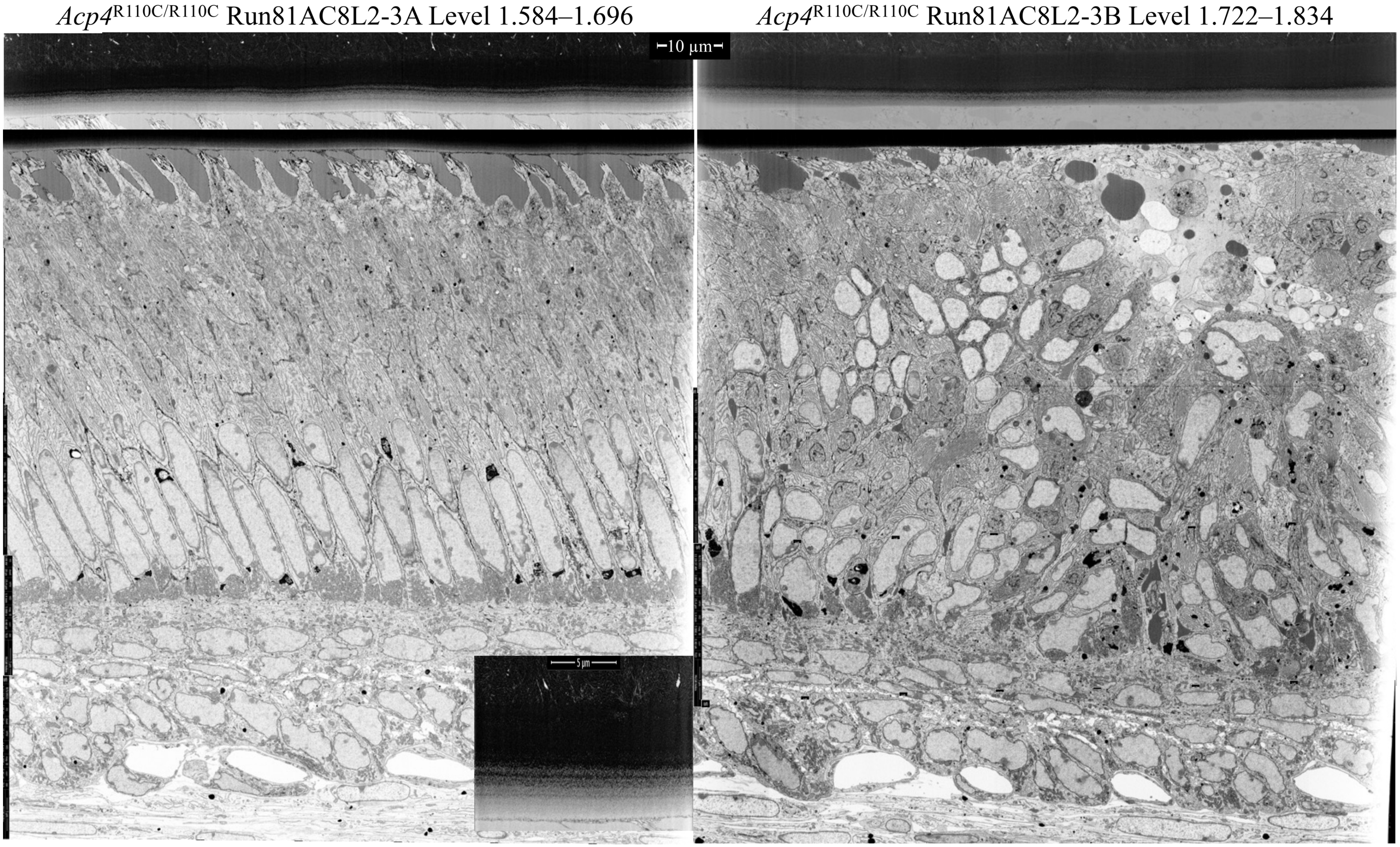

Figure S27A. *Acp4*$^{R110C/R110C}$ incisor longitudinal segments (level 1.584–1.696; left) and further incisally (level 1.722–1.834; right). The 5000x montage on the left is from an *Acp4*$^{R110C/R110C}$ level that in a wild-type mouse would 28.3 µm thick enamel on the left and 41.8 µm on the right. Images are taken at high brightness along the top to assess the thickness of the enamel. The inset on the lower right of the left panel is an enlargement of the over-exposed enamel layer shown on the top right. The dark layer at the top of the inset with white areas of low density is dentin. Below this is the highly mineralized deep layer of enamel slightly over 4 µm thick with multiple layers of less mineralized enamel accumulated on its surface. The bottom of the scale marks the enamel surface. The total thickness of the enamel is about 8 µm. The extracellular matrix contains abundant extracellular protein between the ameloblasts and the enamel surface. The 5000x montage on the right shows that by Level 1.8 the ameloblast layer has degenerated and is no longer a polarized sheet of cells.

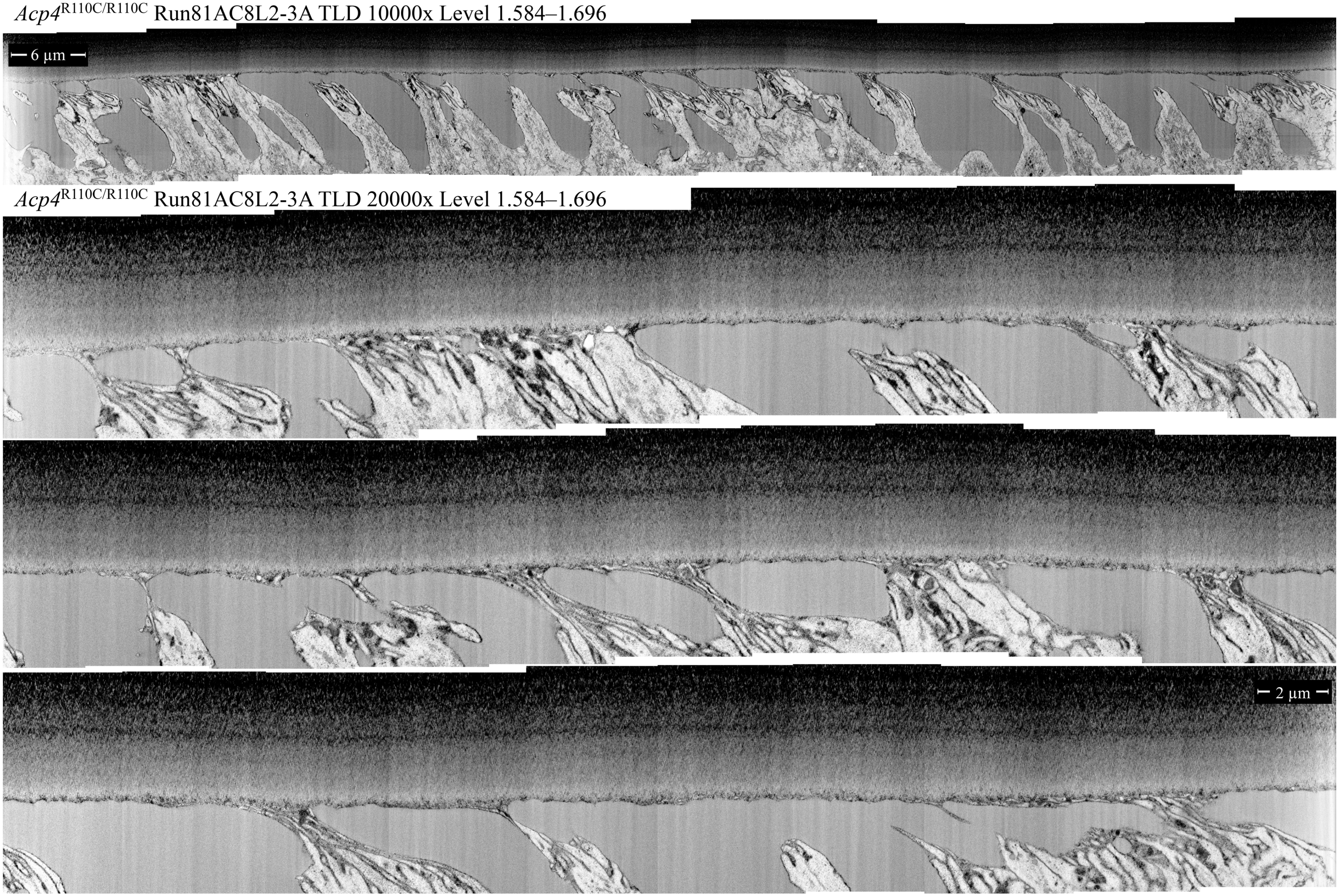

Figure S27B. *Acp4*$^{R110C/R110C}$ incisor longitudinal segment showing bright images at level 1.584–1.696 magnified x10000x (top), and 20000x (bottom). The enamel in this segment is only ~8 µm thick. In wild-type mice the enamel thickness would be ~42 µm. Part of the extracellular material covering the enamel surface shown here likely mineralizes to become some of the "ectopic" mineral nodules that that are seen in routine bSEM images (e.g., Fig. 7).

Acp4[R110C/R110C] Run81AC8L2-3B 10000x Levels 1.722–1.834

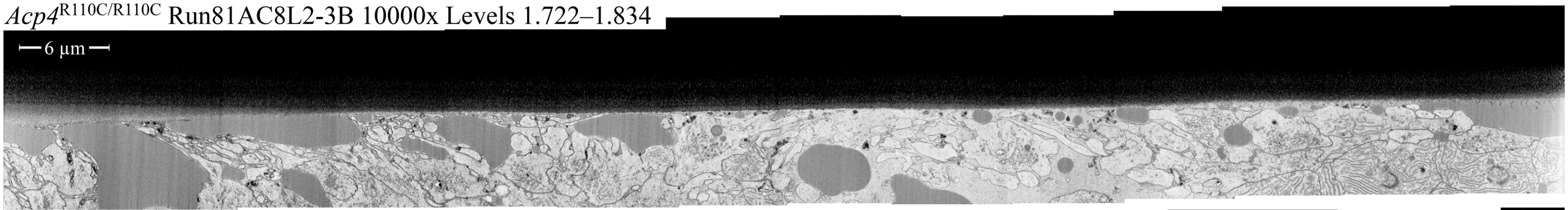

Acp4[R110C/R110C] Run81AC8L2-3B 20000x Levels 1.722–1.834

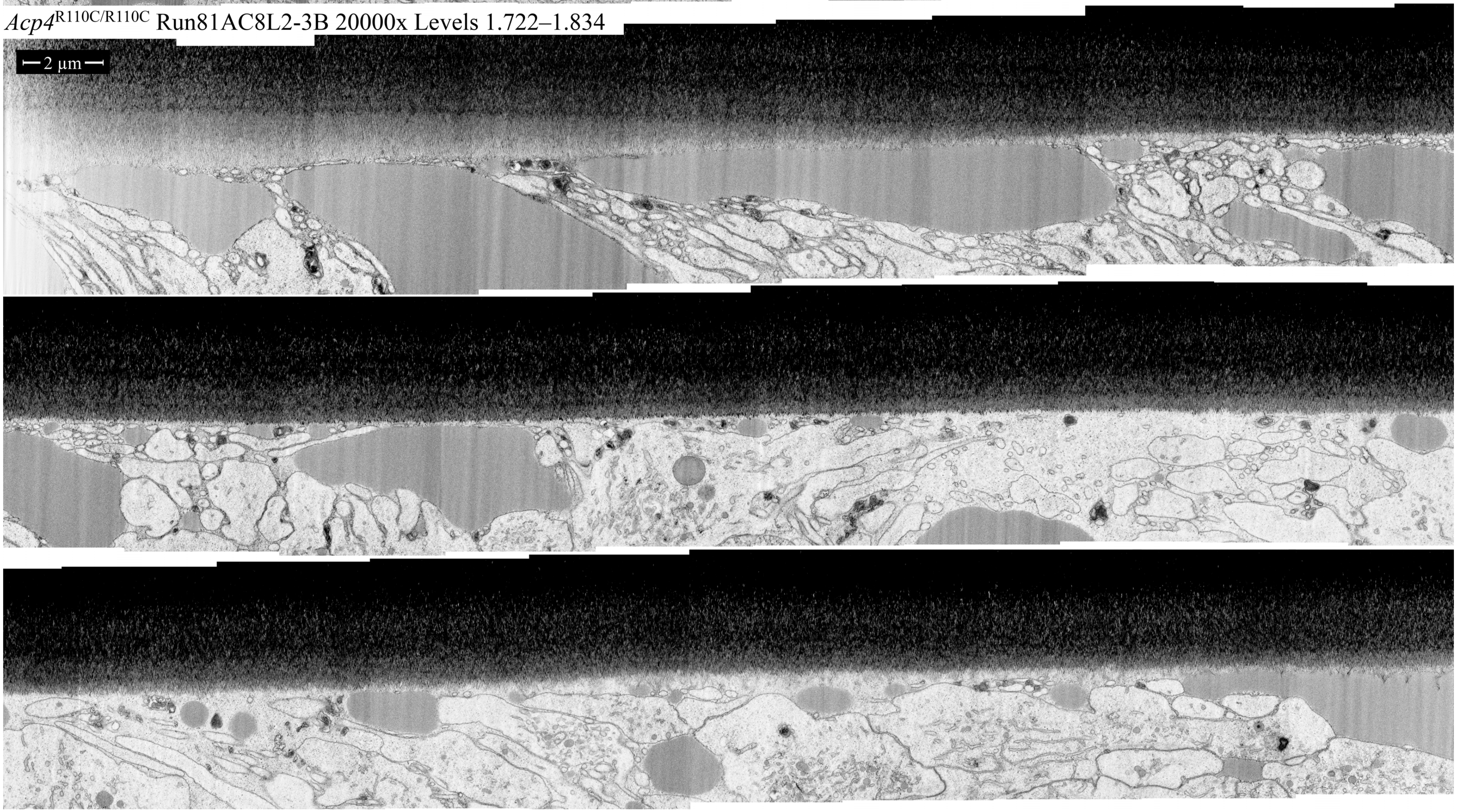

Figure S27C. *Acp4*[R110C/R110C] incisor longitudinal segment showing bright images at level 1.722–1.834 magnified x10000x (top), and 20000x (bottom). In wild-type mice the enamel thickness at the lower right would be ~50 µm. The ameloblast layer is now disorganized and pathological. The aplastic enamel layer will no longer expand in thickness, but instead generates ectopic mineral in extracellular spaces within the soft tissue.
